# Disruption of common ocular developmental pathways in patient-derived optic vesicle models of microphthalmia

**DOI:** 10.1016/j.stemcr.2024.05.001

**Published:** 2024-05-30

**Authors:** Jonathan Eintracht, Nicholas Owen, Philippa Harding, Mariya Moosajee

**Affiliations:** 1 UCL Institute of Ophthalmology, London EC1V 9EL, UK; 2 Moorfields Eye Hospital NHS Foundation Trust, London EC1V 9EL, UK; 3 Francis Crick Institute, London NW1 1AT, UK

**Keywords:** microphthalmia, extracellular matrix, eye morphogenesis, hiPSC-derived optic vesicles, PAX6, apoptosis, proliferation, Z-IETD-FMK, ocular genetics

## Abstract

Genetic perturbations influencing early eye development can result in microphthalmia, anophthalmia, and coloboma (MAC). Over 100 genes are associated with MAC, but little is known about common disease mechanisms. In this study, we generated induced pluripotent stem cell (iPSC)-derived optic vesicles (OVs) from two unrelated microphthalmia patients and healthy controls. At day 20, 35, and 50, microphthalmia patient OV diameters were significantly smaller, recapitulating the “small eye” phenotype. RNA sequencing (RNA-seq) analysis revealed upregulation of apoptosis-initiating and extracellular matrix (ECM) genes at day 20 and 35. Western blot and immunohistochemistry revealed increased expression of lumican, nidogen, and collagen type IV, suggesting ECM overproduction. Increased apoptosis was observed in microphthalmia OVs with reduced phospho-histone 3 (pH3+) cells confirming decreased cell proliferation at day 35. Pharmacological inhibition of caspase-8 activity with Z-IETD-FMK decreased apoptosis in one patient model, highlighting a potential therapeutic approach. These data reveal shared pathophysiological mechanisms contributing to a microphthalmia phenotype.

## Highlights

• Microphthalmia patient-specific optic vesicles recapitulate the “small eye” phenotype
• Global upregulation of extracellular matrix in microphthalmia
• Aberrant apoptosis and cell proliferation contribute to reduced optic vesicle size
• Caspase-8 inhibition reduced apoptosis in one microphthalmia patient model

## Teaser

Moosajee and colleagues present possible shared developmental disruptions causing microphthalmia through the generation of human induced pluripotent stem cell-derived optic vesicles. Patient vesicles had reduced diameter, recapitulating the small eye phenotype. RNA and protein analysis revealed global upregulation and altered expression patterns of extracellular matrix proteins, as well as increased apoptosis and reduced cell proliferation in microphthalmia, suggesting common disease pathways in microphthalmia.

## Introduction

Eye morphogenesis is tightly regulated by highly conserved gene regulatory networks (GRNs) of transcription factors such as *PAX6*, *MITF*, *RAX*, and *SOX2* that drive eye-field formation in the anterior neural plate (Eintracht et al., 2020a; Mic et al., 2004; Steinfeld et al., 2013).

Disruptions to these GRNs can result in the clinical spectrum of structural eye malformations including microphthalmia, anophthalmia, and ocular coloboma (collectively termed MAC) (Eintracht et al., 2020a). Microphthalmia is defined as a small, underdeveloped eye (axial length of <19 mm at 1 year of age or <21 mm in adulthood); anophthalmia is the absence of an eye; and ocular coloboma is a failure of optic fissure fusion, resulting in a persistent inferonasal tissue defect within the eye (Eintracht et al., 2020a). There can be significant overlap with a mixed clinical phenotype, but they can also appear in isolation or with other systemic features (Eintracht et al., 2020b). Despite over 100 genes being associated with MAC, a recent study suggested only 33% of patients receive a molecular diagnosis while other studies estimate a lower positive molecular diagnosis rate at between 20% and 30% (Harding et al., 2022; Basharat et al., 2023; Plaisancié et al., 2019). The majority of solved cases arise from variants in eye-field transcription factors (EFTFs) such as *SOX2*, *OTX2*, and *PAX6* (Eintracht et al., 2020b; Williamson and FitzPatrick, 2014). Other pathogenic variants affect pathways including retinoic acid, bone morphogenetic protein (BMP), and transforming growth factor β (TGF-β) signaling that have key roles in oculogenesis (Williamson and FitzPatrick, 2014; Deml et al., 2014). Although the disruptive effects of specific genes on early eye development have been extensively characterized, there remains no comparative studies describing shared or common disease pathways causing ocular malformation independent of genotype (Gamm et al., 2019; Capowski et al., 2016; Phillips et al., 2014; den Hollander et al., 2010; French et al., 2013; Williamson et al., 2020; Wu et al., 2003).

The correct spatiotemporal expression of cell cycle and proliferation genes is critical for eye growth (Asaoka et al., 2014; Green et al., 2003; Yamaguchi et al., 2005). Pathogenic variants that directly influence the expression of such genes may alter the dynamics of cell division and death in the developing eye, resulting in microphthalmia (Phillips et al., 2014; French et al., 2013; den Hollander et al., 2010; Xu et al., 2007; Kim et al., 2016). For instance, apoptosis was increased by an *Msx2* variant that activated the caspase-3/8 pathway and by the loss of *Rbm* that abolished Birc2 inhibition of anti-apoptotic pathways in mouse *Msx2* models of microphthalmia (Yu et al., 2018). Additionally, upregulation of some pro-apoptotic and cell cycle regulatory genes has been described in *in vitro* microphthalmia studies showing reduced optic vesicle (OV) size in microphthalmia patient-derived models (Capowski et al., 2016; Phillips et al., 2014).

Alongside the intrinsic molecular cues that guide ocular development, extrinsic cues generate both the biophysical conditions driving optic cup morphogenesis and the extracellular signals to create a unique microenvironment for the developing eye (Bryan et al., 2020; Casey et al., 2021; Serjanov et al., 2018). Contact between the surface ectoderm and the neuroectodermal OV is mediated through the extracellular matrix (ECM) secreted by both tissues (Lee e t al., 2021). The ECM has a critical role in eye development due to the physical limits placed on lateral OV growth that drives optic cup invagination (Oltean et al., 2016). Retinal progenitor proliferation and differentiation are also modulated by the ECM via cues that alter cell cycle dynamics (Serjanov et al., 2018).

Disruptions to laminin sub-units α, β, and γ have resulted in ocular malformations via loss of basement membrane (BM) integrity, cell polarity, or focal adhesion assembly (Serjanov et al., 2018; Bryan et al., 2016; Guo et al., 2019). The ECM protein nidogen secreted by the BM has a critical role in zebrafish oculogenesis as its disruption impaired optic cup formation (Bryan et al., 2020; Carrara et al., 2019). Exogenous nidogen protein supplemented to *tfap2a;foxd3* double-mutant zebrafish that lacked neural crest cells, ablating the BM, led to partial rescue of defective optic cup invagination and optic fissure closure (Bryan et al., 2020). The ECM component lumican (*LUM*) has a role in axial length control as *Lum*^*−/−*^*/Fmod*^*−/−*^ double knockout mice and *lum*-morpholino zebrafish all had significantly elongated axial lengths compared to wild type (WT) at 8 weeks of age and 22 days post fertilization (dpf), respectively (Yeh et al., 2010; Song et al., 2016). Additionally, collagen type IV variants or expression level changes have been associated with ocular maldevelopment, while *COL4A1/4* regulates retinal pigment epithelium (RPE) growth and function *in vitro* (Deml et al., 2014; Eamegdool et al., 2020; Bai et al., 2009; Sijilmassi et al., 2021; Matías-Pérez et al., 2018). This suggests a critical role for the ECM in early ocular development, yet ECM dysfunction has yet to be described in microphthalmia.

In this study, we successfully identify shared morphological and molecular pathways between unrelated microphthalmia patients from different families, a bilateral microphthalmia patient who remained clinically unsolved following whole-genome sequencing and a unilateral microphthalmia patient with a *PAX6* heterozygous missense variant (c.372C>A) p.(Asn124Lys). Two common disease mechanisms were detected: (i) increased apoptosis, reduced proliferation, and cell cycle misregulation and (ii) overproduction of ECM components that may overly constrict eye growth resulting in microphthalmia. Our findings advance our understanding of GRNs controlling early eye development and the downstream effects of their disruption, while highlighting novel gene targets to improve future molecular diagnostics. This may also enhance our understanding of the large phenotypic heterogeneity associated with MAC and provide therapeutic targets for development to a larger cohort of patients without relying on a genetic diagnosis.

## Results

### RNA-seq analysis revealed global transcriptomic changes to early ocular developmental pathways

To investigate shared pathophysiology between unrelated microphthalmia patients, bulk RNA sequencing (RNA-seq) was performed to create transcriptomic profiles of both healthy and patient-derived OVs. High-quality RNA was extracted from a WT line, patient one (P1, unsolved genetic diagnosis following whole-genome sequencing and full targeted gene panel screen of structural eye disease genes [Patel et al., 2019]), and patient two (P2, with *PAX6*-heterozygous missense (c.372C>A) p.(Asn124Lys) variant, confirmed pathogenic for microphthalmia) (Méjécase et al., 2020; Harding et al., 2021). Further clinical details of both patients can be found in Table S1. Days 20 and 35 were chosen as time points corresponding to OV and cup formation, respectively, as expression of ocular markers at these time points correlated to these milestones (Eintracht et al., 2022).

Analysis was carried out on two clones per line and four replicates per condition per time point. High-quality reads were mapped to human genome GRCh38, annotation version 102. At day 20, principal component analysis (PCA) demonstrated P1 samples clustered together while P2 shared some overlap with WT (Figure S1). At day 35, PCA of the data showed clustering of P1 and P2 together distinct from WT, suggesting some shared variance between microphthalmia patients compared to WT (Figure S1). At both day 20 and 35, principal component Pearson r^2^ clinical correlates showed strong clustering of samples based on condition (Figure S1).

**Figure 1:**
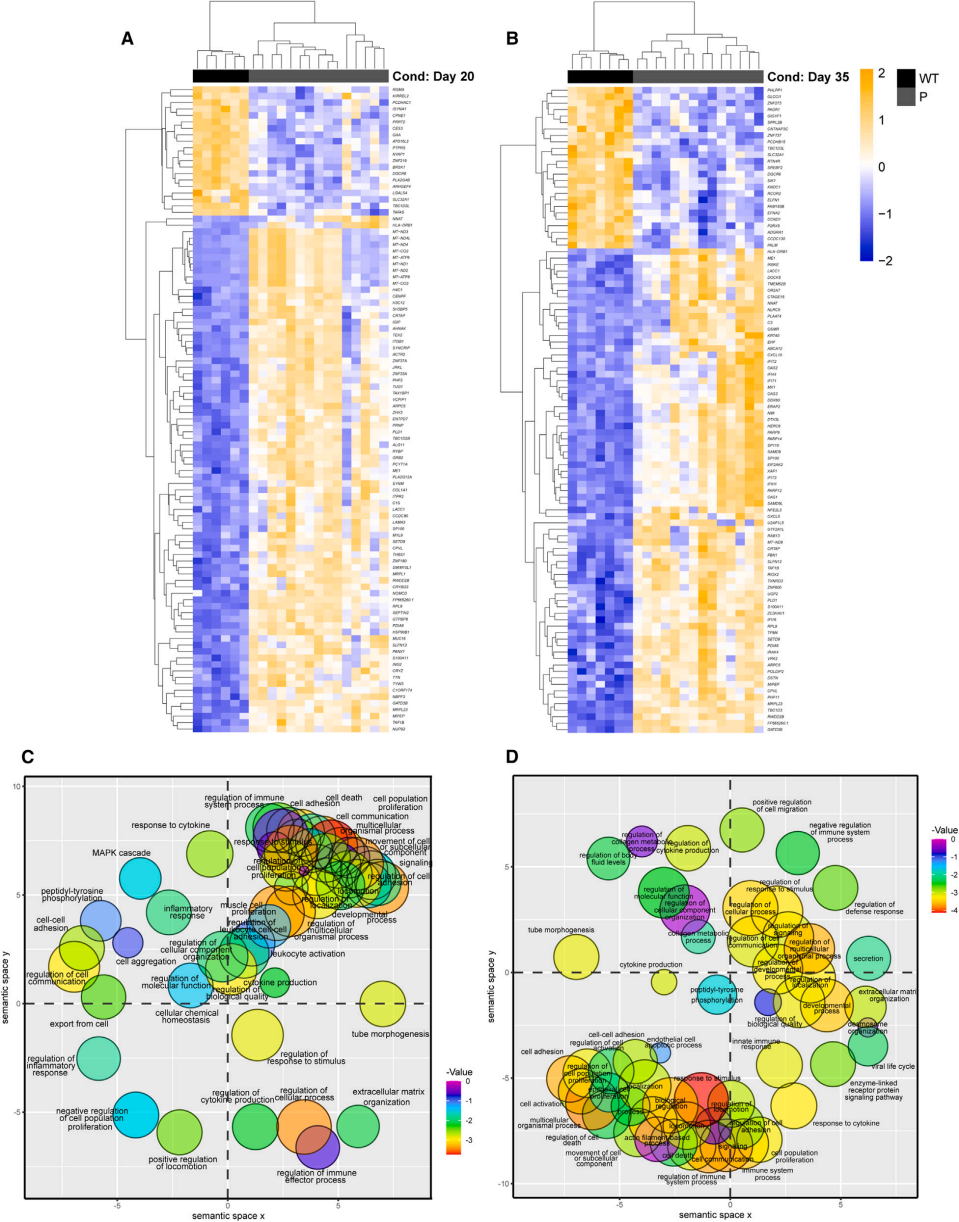
Transcriptomic profiling of microphthalmia optic vesicles (A) Heatmap of top 100 differentially expressed genes between wild-type (WT) and patient (P) optic vesicles at day 20. (B) Heatmap of top 100 differentially expressed genes between WT and patients at day 35. (C) Enriched gene ontology (biological process) terms between WT and P at day 20. (D) Enriched gene ontology (biological process) terms between WT and P at day 35. RNA-seq analysis was performed with *n* = 4 from two clones each per time point per condition.

Groupwise and pairwise comparisons of all possible combinations were employed to identify differentially expressed genes (DEGs). DEGs were identified with a log_2_fold change (LFC) ≥+1 or ≤ −1 and a false discovery rate adjusted *p* value <0.05. Filtered DEG outputs for each comparison are presented in supplemental information (Tables S2 and S3). Initially, both patients were compared to WT in a three-way groupwise comparison to reveal any shared developmental deviations in early ocular morphogenesis. Additionally, patient samples were grouped together for pairwise analysis of patient P vs. WT conditions. Subsequently, results were confirmed with pairwise comparisons of each patient compared to WT and validated with quantitative reverse-transcription PCR (Figure S2). DEGs were reported in reference to WT in each comparison. At day 20, the P vs. WT comparison identified 1,865 DEGs in microphthalmia patients; 1,329 upregulated and 536 downregulated (Table S2). At day 20, transcriptomic changes to ocular development were evident as EFTFs *RAX* (LFC −2.671, *p* < 0.00247), *PAX6* (LFC −1.191, *p* < 0.0308), *SIX6* (LFC −2.612, *p* < 0.0000154), *HES5* (LFC −2.03, *p* < 0.0119), and *LHX5* (LFC −2.420, *p* < 0.0000326) were downregulated while *GDF6* (LFC +1.674, *p* < 0.000028), part of the TGF-β family active in early development, and histone deacetylase *HDAC1* (LFC +1.239, *p* < 0.000117) that also plays a close role in progenitor cell proliferation and differentiation were upregulated (Figure 1A; Table S2). At the day 20 time point, upregulation of mitochondrial-related genes such as *MRPL3* (LFC +5.06, *p* < 6.06 × 10^−14^), *MT-ATP6* (LFC +4.65, *p* < 1.30 × 10^−9^), and *MT-ND2* (LFC +4.56, *p* < 8.01 × 10^−10^) was also observed (Figure 1A; Table S2). At day 35, the P vs. WT comparison identified 2,042 DEGs; 1,598 upregulated and 444 downregulated relative to WT (Table S3). At day 35, we measured downregulation of key ocular development genes such as *HES5* (LFC −1.651, *p* < 0.00177), *LHX1* (LFC −2.148, *p* < 0.0000254), and *LHX5* (LFC −2.117, *p* < 0.0000008) and upregulation of *MITF* (LFC +1.212, *p* < 0.000420) and *BMP4* (LFC +1.116, *p* < 0.00329) (Figure 1B; Table S3).

Unbiased hierarchical cluster analysis on significant DEG events at each time point identified gene co-regulation or functionally related DEGs at the identical time point and these data were visualized through heatmaps (Figures 1A and 1B). Individual clades were assigned general groups following visual examination of expression patterns. Gene Ontology (GO) enrichment analysis was performed on clades passing through data filters. At day 20, DEGs were enriched in many early developmental processes such as system development (GO:0048731), animal organ development (GO:0048513), and anatomical structure morphogenesis (GO:0009653) (Figure 1C; Table S4). Other enriched GO terms were globally affecting cell function such as multicellular organismal process (GO:0032501), biological adhesion (GO:0022610), signaling (GO:0023502), and cell differentiation (GO:0030154).

At day 35, enriched GO terms were still related to early ocular development such as response to retinoic acid (GO:0032526), tissue development (GO:0009888), and regulation of anatomical structure morphogenesis (GO:0022603) (Figure 1D; Table S5). Other enriched GO terms included response to stimulus (GO:0050896), cell adhesion (GO:0007155), and cell surface receptor signaling pathway (GO:0007166).

### RNA-seq analysis highlighted global upregulation of ECM-associated genes

**Figure 2:**
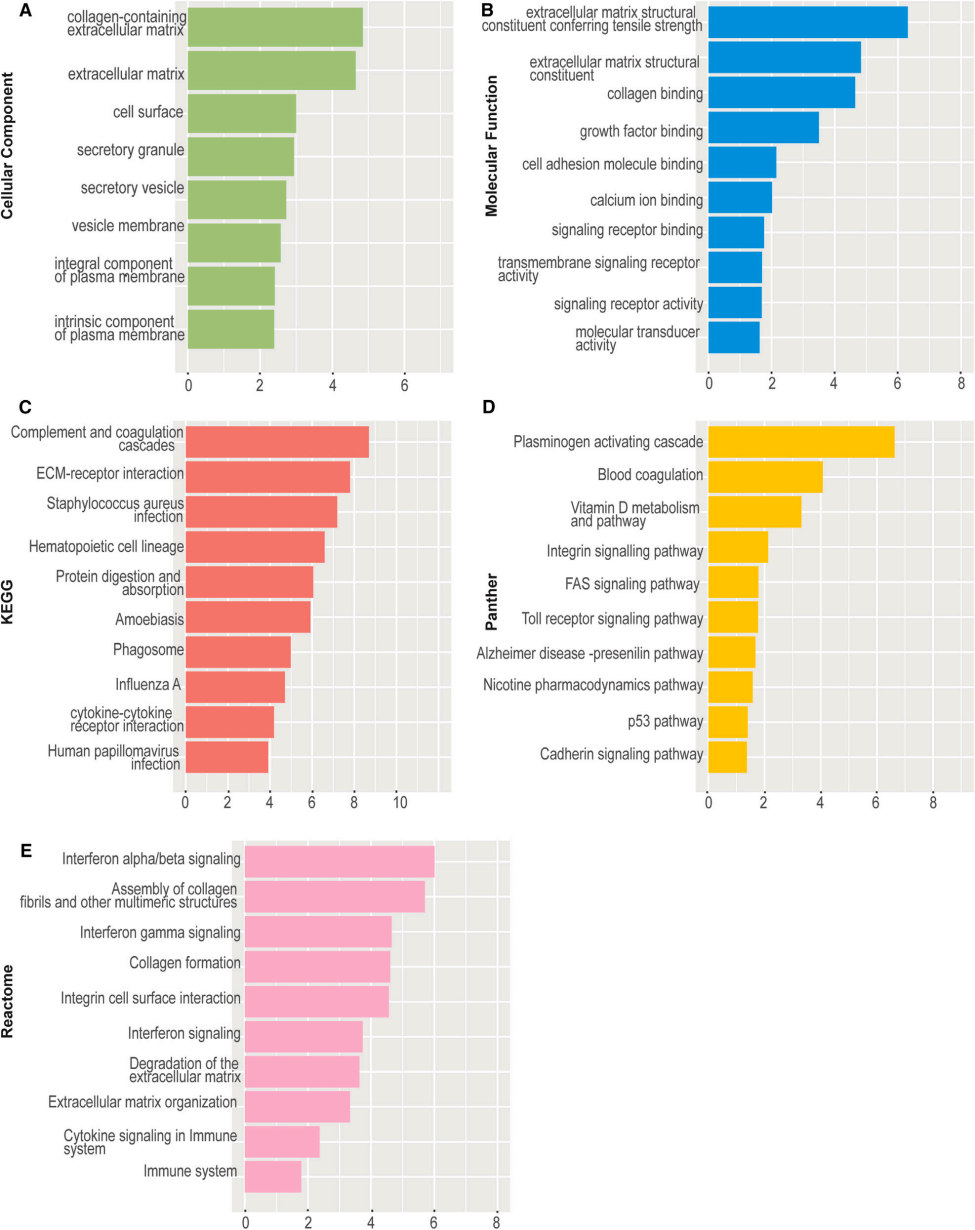
Gene Ontology (GO) term enrichment analysis of WT vs. P (patient-derived) optic vesicles at day 35 (A) The highest enriched cellular compartment GO terms in WT vs. P at day 35 generated using RNA-seq data. (B) The highest enriched molecular function GO terms in WT vs. P at day 35 generated using RNA-seq data. (C) The highest enriched KEGG GO terms in WT vs. P at day 35 generated using RNA-seq data. (D) The highest enriched Panther GO terms in WT vs. P at day 35 generated using RNA-seq data. (E) The highest enriched Reactome GO terms in WT vs. P at day 35 generated using RNA-seq data. RNA-seq analysis was performed with *n* = 4 from two clones each per time point per condition.

GO term enrichment analysis at day 20 showed upregulated genes were enriched for ECM modeling such as extracellular matrix organization (GO:0030198), extracellular structure organization (GO:0043062), and extracellular matrix (GO:0031012). At day 35, ECM-related gene ontologies were the most significantly enriched in microphthalmia patients (Figure 2; Tables S4 and S5). This included enrichment in GO: cellular compartment structures such as collagen-containing extracellular matrix (GO:0062023) (Figure 2A) and GO: molecular function terms including extracellular matrix structural constituent conferring tensile strength (GO:0030020), (Figure 2B). We also observed enrichment in Kyoto Encyclopedia of Genes and Genomes (KEGG) pathway-related terms including ECM-receptor interaction (KEGG:04512) (Figure 2C), Panther-related terms such as plasminogen activating cascade, integrin signaling pathway, and cadherin signaling pathway that act in the ECM region (Figure 2D), and for Reactome terms, this included assembly of collagen fibrils and other multimeric structures (REAC:R-HSA-2022090), collagen formation (REAC:R-HSA-1474290), and extracellular matrix organization (REAC:R-HSA-1474244) and ECM proteoglycans (REAC:R-HSA-3000178) (Figure 2E). Additionally, GO:BP term analysis revealed enrichment of integrin-related terms such as integrin-mediated signaling pathway (GO:0007229) and integrin binding (GO:0005178).

Subsequent analysis aimed to identify specific ECM-associated genes upregulated in the patient OVs. At time points day 20 and 35, upregulation of ECM-associated genes such as *LUM* ([day 20 LFC +1.88, *p* < 0.00280] [day 35 LFC +3.12, *p* < 3.70 × 10^−4^]), *COL4A4* (day 35 LFC +1.47, *p* < 3.70 × 10^−3^), *LAMB4* ([day 20 LFC +2.11, *p* < 3.69 × 10^−3^] [day 35 LFC +2.05, *p* < 4.00 × 10^−3^]), *DSC3* ([day 20 LFC +2.12, *p* < 3.99 × 10^−5^] [day 35 LFC +2.04, *p* < 2.41 × 10^−5^]), and *NID2* ([day 20 LFC +1.12, *p* < 1.00 × 10^−2^] [day 35 LFC +1.35, *p* < 1.51 × 10^−5^]) was measured (Tables S2 and S3).

### The extracellular matrix is enriched in microphthalmia patient OVs throughout early development

**Figure 3:**
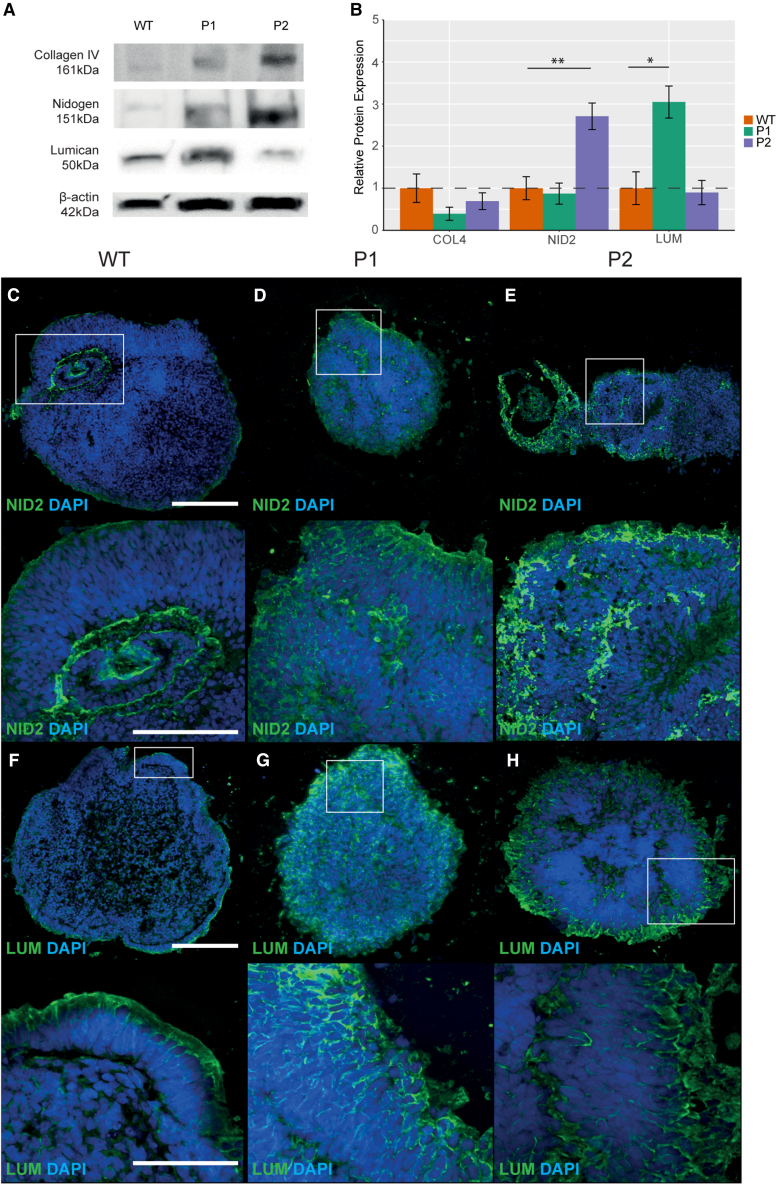
Expression of ECM proteins in day 20 optic vesicles (A) Western blot showing expression levels of COL4A1, NID2, and LUM in WT, P1 (patient 1), and P2 (patient 2) optic vesicles at day 20. β-Actin was used as a loading control for normalization of expression levels. (B) Quantification of COL4A1, NID2, and LUM expression levels from the western blot. (C–E) Expression patterns of NID2 in WT, P1, and P2 optic vesicles at day 20. (F–H) Expression patterns of LUM in WT, P1, and P2 optic vesicles at day 20. Experiments were performed with *n* = 3 from one clone per condition. *p* < 0.05 (^∗^), *p* < 0.01 (^∗∗^), *p* < 0.001 (^∗∗∗^). All results are expressed as mean ± SD.

To further understand ECM overproduction in microphthalmia, LUM, COL4A1, and NID2 were selected for further analysis due to their specific role in eye development and prior association with ocular maldevelopment (Bryan et al., 2020, Sijilmassi et al., 2021, Song et al., 2016, Yeh et al., 2010). We investigated whether LUM, COL4A1, and NID2 protein expression levels and patterns differed between WT and patient OVs. Western blot at day 20 highlighted significant upregulation of LUM 3.05-fold (*p* < 0.0132, *n* = 3) in P1 only, while nidogen was significantly upregulated in P2 only 2.99-fold (*p* < 2.21 × 10^−3^, *n* = 3) (Figures 3A and 3B). COL4A1 levels did not significantly differ between WTs and patients at day 20 (*p* < 3.76 × 10^−1^, *n* = 3; *p* < 9.01 × 10^−1^, *n* = 3) (Figures 3A and 3B). In WT, NID2 expression was confined to the basal layer of the OV; however, its expression was ubiquitous throughout patient OVs (Figures 3C–3E). LUM expression was confined to the apical aspect of both WT and patient OVs at day 20, yet expression intensity was higher in patients (Figures 3F–3H).

**Figure 4:**
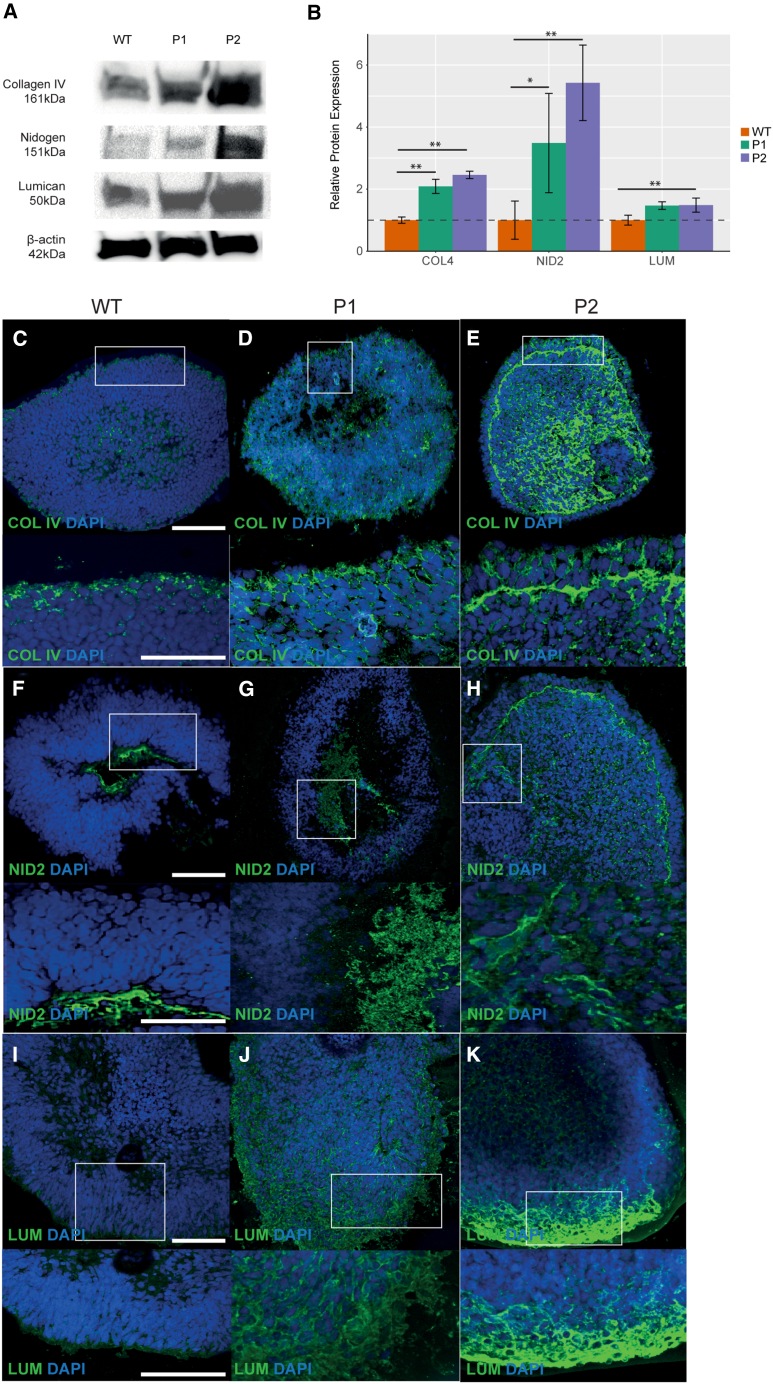
Expression of ECM proteins in day 35 optic vesicles (A) Western blot showing expression levels of COL4A1, NID2, and LUM in WT, P1, and P2 optic vesicles at day 35. β-Actin was used as a loading control for normalization of expression levels. (B) Quantification of COL4A1, NID2, and LUM expression levels from the western blot. (C–E) Expression patterns of COL4A1 in WT, P1, and P2 optic vesicles at day 35. (F–H) Expression patterns of NID2 in WT, P1, and P2 optic vesicles at day 35. (I–K) Expression patterns of LUM in WT, P1, and P2 optic vesicles at day 35. Experiments were performed with *n* = 3 from one clone per condition. *p* < 0.05 (^∗^), *p* < 0.01 (^∗∗^), *p* < 0.001 (^∗∗∗^). All results are expressed as mean ± SD.

At day 35, NID2 was significantly upregulated 4.06-fold in P1 (*p* < 0.0398, *n* = 3) and 5.4-fold in P2 (*p* < 0.0282, *n* = 3) (Figures 4A and 4B). LUM was upregulated 1.5-fold in P1 and P2, yet this was only significant in P2 (*p* < 0.0214 × 10^−2^) (Figures 4A and 4B). Similar to NID2, COL4A1 expression was significantly upregulated in both P1 and P2 OVs 2.1-fold (*p* < 0.0136, *n* = 3) and 3.2-fold (*p* < 0.0154, *n* = 3), respectively (Figures 4A and 4B). NID2 expression was again confined to the basal aspect of WT OVs (Figures 4C–4E). NID2 was detected at the basal aspect of P1 OVs, yet in P2, NID2 was detected at the basal aspect and throughout the inner cell layers of the OV (Figures 4C–4E). LUM expression was strongest at the apical aspect of WT OVs (Figures 4F–4H). In P1, LUM was observed at the apical aspect of the OV, although it was detected throughout P2 OVs (Figures 4F–4H). COL4A1 expression was observed at the basal and apical aspects of WT OVs yet in both P1 and P2, COL4A1 expression was ubiquitous (Figures 4I–4K).

### RNA-seq analysis revealed global upregulation of pro-apoptotic genes, changes to regulatory cell cycle, and proliferation-related genes

At day 20, pro-apoptotic genes such as *CARD16* (LFC +2.36, *p* < 0.0105), *CASP1* (LFC +5.53, *p* < 0.00000713), *CASP8* (LFC +1.65, *p* < 0.000391), and *XAF1* (LFC +2.33, *p* < 0.00000777) were upregulated. Frizzled receptors acting in the Wnt signaling pathway *FZD9* (LFC −1.28, *p* < 0.000121) (for which haploinsufficiency was previously associated with increased apoptosis in neural progenitor cells [Chailangkarn et al., 2016]), *FZD10* (LFC −2.34, *p* < 0.0185), and FZD9 interactor *APC2* (LFC −1.79, *p* < 0.000583) were downregulated. Wnt ligands and Wnt interactors were differentially expressed, suggesting major dysregulation of the Wnt signaling pathway associated with loss of frizzled receptor expression. Additionally, changes to cell cycle genes *CDKN2B* (LFC +2.10, *p* < 0.0000609), *CDKN2A* (LFC +2.68, *p* < 0.00119), and *CDK15* (LFC +1.55, *p* < 0.00457) were detected while TGF-β-related genes were also upregulated (Figure 1A; Table S2).

At day 35, more marked changes to expression levels of apoptosis genes were measured. Caspase recruiters *CARD6* (LFC +1.949, *p* < 0.000179), *CARD11* (LFC +1.89, *p* < 0.0000553), and *CARD16* (LFC +6.06, *p* < 0.0000654) were all significantly upregulated alongside caspases including *CASP1* (LFC +4.77, *p* < 0.000000954), *CASP4* (LFC +3.85, *p* < 0.000000106), and *CASP8* (LFC +2.46, *p* < 0.0000038). This global upregulation of pro-apoptotic genes was detected also in the tumor necrosis family (TNF) involved in the extrinsic apoptosis pathway (Figure 1B; Table S3).

Further upstream of the caspases in the apoptosis pathway, *XAF1* (LFC +5.26, *p* < 0.0001), *IRF1* (LFC +1.53, *p* < 0.000341), and *STAT1* (LFC +1.850, *p* < 0.000000118) and pro-apoptotic genes *BIRC3* (LFC +2.39, *p* < 0.00000695) and *BIK* (LFC +1.43, *p* < 0.00178) was upregulated in microphthalmia patients (Figure 1B; Table S3). At the day 20 time point, *FZD9* (LFC −1.22, *p* < 0.0000484) and *FZD10* (LFC −2.11, *p* < 0.00228) were downregulated. *APC2*, an FZD9 interactor, was also downregulated (LFC −1.61, *p* < 0.00000559). Upstream of the FZD9 receptor, Wnt ligands were differentially expressed, while Wnt inhibitory factor *WIF1* was also upregulated (LFC +1.05, *p* < 0.0125) (Figure 1B; Table S2).

DEGs involved in cell cycle regulation were also measured. Negative regulators of proliferation *CDKN2A* (LFC +2.10, *p* < 0.00042), *CDKN2B* (LFC +2.45, *p* < 0.000000426), and *JUNB* (LFC +1.69, *p* < 0.000177) were upregulated while cyclins *CCNP* (LFC −1.15, *p* < 0.00163) and *CCNA1* (LFC −1.28, *p* < 0.00435) were downregulated (Figure 1B; Table S2). The expression of cell cycle regulator *TMEM30B* (LFC +1.21, *p* < 0.0231) and FGF pathway genes regulating proliferation also differed between microphthalmia patients and WT (Figure 1B; Table S2).

The DEGs at day 35 were enriched for proliferation GO terms including positive regulation of cell population proliferation (GO:0042127) and negative regulation of cell population proliferation (GO:0008285) (Figure 1D; Table S5). However, clusters of genes with higher expression in microphthalmia patients were primarily enriched in apoptosis-related terms including cell death (GO:0008219), programmed cell death (GO:0012501), apoptotic signaling pathway (GO:0097190), and extrinsic apoptotic signaling pathway (GO:0097191) (Figure 1D; Table S5).

### Microphthalmia patient OVs exhibited reduced diameter and cell proliferation with increased apoptosis

**Figure 5:**
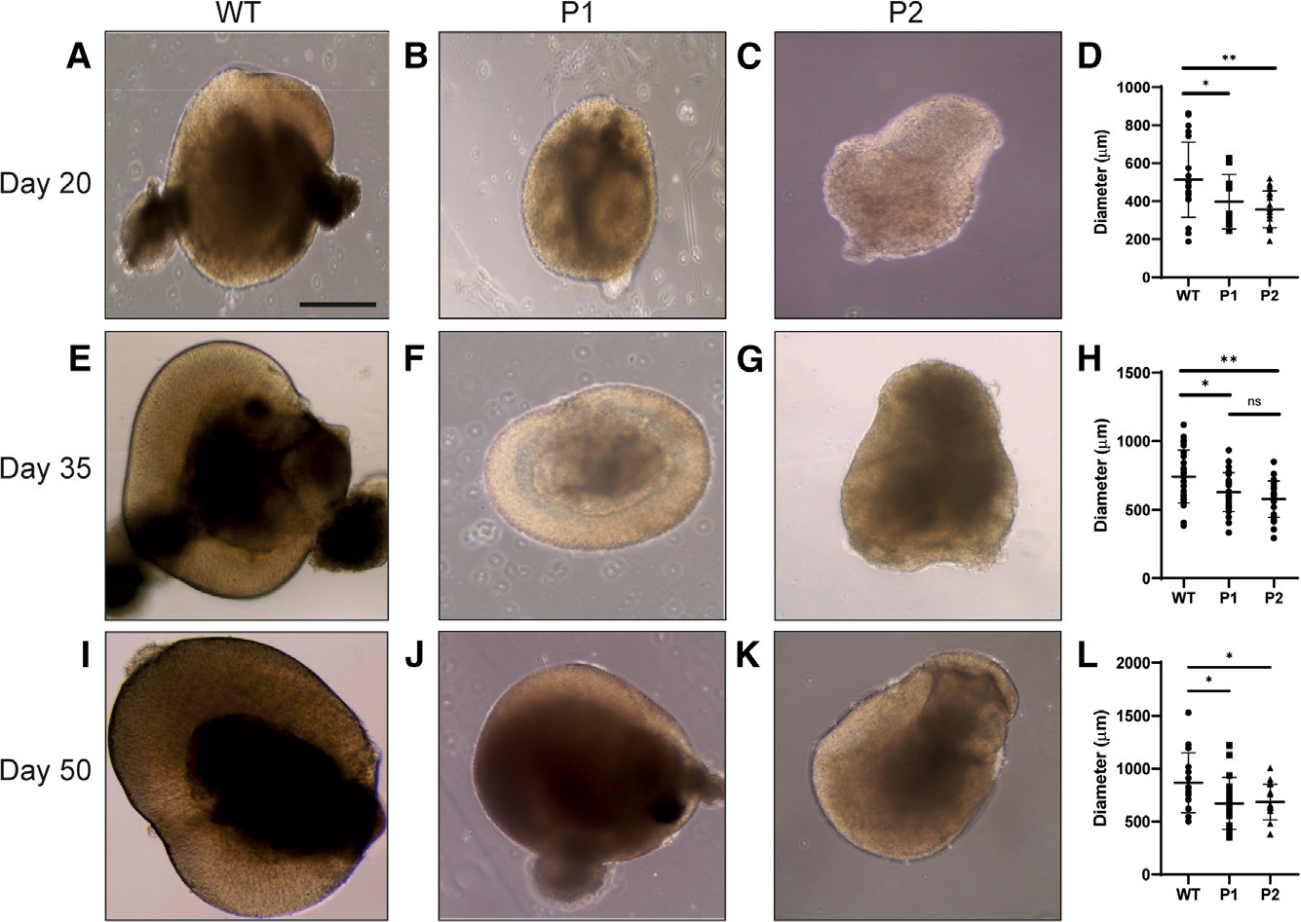
Diameters of WT, P1, and P2 optic vesicles (A–C) Light microscopy images of WT, P1, and P2 optic vesicles at day 20. (D) Diameters of WT, P1, and P2 optic vesicles at day 20. (E–G) Light microscopy images of WT, P1, and P2 optic vesicles at day 35. (H) Diameters of WT, P1, and P2 optic vesicles at day 35. (I–K) Light microscopy images of WT, P1, and P2 optic vesicles at day 50. (L) Diameters of WT, P1, and P2 optic vesicles at day 50. For all conditions, *n* = 20. *p* < 0.05 (^∗^), *p* < 0.01 (^∗∗^), *p* < 0.001 (^∗∗∗^). All results are expressed as mean ± SD.

The diameter of WT and patient OVs was measured at day 20, 35, and 50 to ascertain whether the “small eye” phenotype was recapitulated *in vitro*. At day 20, the mean diameter of WT OVs measured 513.10 ± 198.45 μm (*n* = 20) (Figures 5A–5D). This was significantly larger than both P1 at 397.64 ± 143.50 μm (*p* < 0.0423, *n* = 20) and P2 at 356.64 ± 96.82 μm (*p* < 0.0038, *n* = 20) (Figures 5A–5D). There were no significant differences between average diameters of P1 and P2 OVs (*p* < 0.301, *n* = 20) (Figure 5D).

At day 35, the mean diameter of WT OVs measured 741.65 ± 192.66 μm (*n* = 20) (Figure 5E). This was still significantly larger than both P1 at 629.04 ± 141.80 μm (*p* < 0.0205, *n* = 20) and P2 at 577.84 ± 132.62 μm (*p* < 0.000869, *n* = 20) (Figures 5E–5H). There were no significant differences between average diameters of P1 and P2 OVs (*p* < 0.185, *n* = 20) (Figure 5H).

At day 50, WT OVs had a mean diameter of 867.58 ± 282.71 μm that remained significantly larger than P1 at 671.66 ± 245.03 μm (*p* < 0.0444, *n* = 20) and P2 at 684.51 ± 166.41 μm (*p* < 0.0401, *n* = 20) (Figures 5I–5L). Similarly, the mean diameters of P1 and P2 OVs did not significantly differ (*p* < 0.858, *n* = 20) (Figure 5L). The mean diameters between day 35 and 50 WT OVs did not differ significantly (*p* < 0.0980, *n* = 20). OV diameters between day 35 and day 50 did not significantly differ in P1 (*p* < 0.469, *n* = 20), yet did significantly differ in P2 (*p* < 0.0270, *n* = 20).

**Figure 6:**
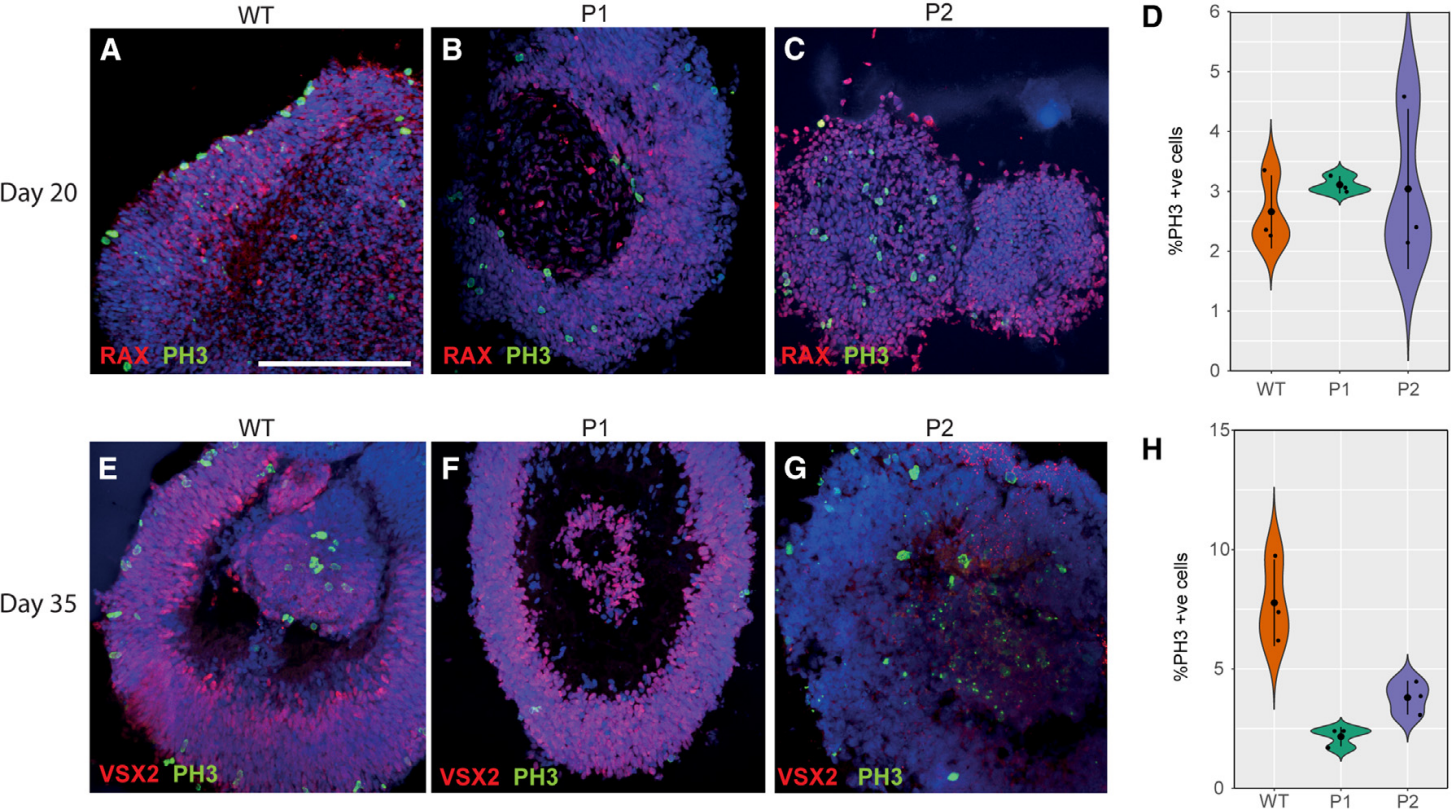
Reduced proliferation in microphthalmia optic vesicles (A–C) pH3+ cells detected in WT, P1, and P2 optic vesicles at day 20. (D) Quantification of pH3+ cells in WT, P1, and P2 optic vesicles at day 20. (E–G) pH3+ cells detected in WT, P1, and P2 optic vesicles at day 35. (H) Quantification of pH3+ cells in WT, P1, and P2 optic vesicles at day 35. Experiments were performed with *n* = 3 from two clones per condition. *p* < 0.05 (^∗^), *p* < 0.01 (^∗∗^), *p* < 0.001 (^∗∗∗^). All results are expressed as mean ± SD.

Reduced OV diameter in microphthalmia patients may be a consequence of both reduced cell proliferation and increased cell death as highlighted from the RNA-seq analysis. To measure cell proliferation, OVs were analyzed by immunostaining to detect the presence of phospho-histone 3 (pH3) in cells currently undergoing mitosis. At day 20, no significant differences were detected between pH3+ cells in WT at 2.66% ± 0.61% of cells compared to P1 at 3.11% ± 0.14% (*p <* 0.282, *n* = 3) and P2 at 3.04% ± 1.34% (*p* < 0.687, *n* = 3) (Figures 6A–6D). At day 35, the proportion of pH3+ cells was significantly reduced in both P1 at 2.17% ± 0.40% of cells (*p* < 0.0281, *n* = 3) and P2 at 3.80% ± 0.70% of cells (*p* < 0.0481, *n* = 3) compared to WT OVs at 7.77% ± 1.81% of cells (Figures 6E–6H).

**Figure 7:**
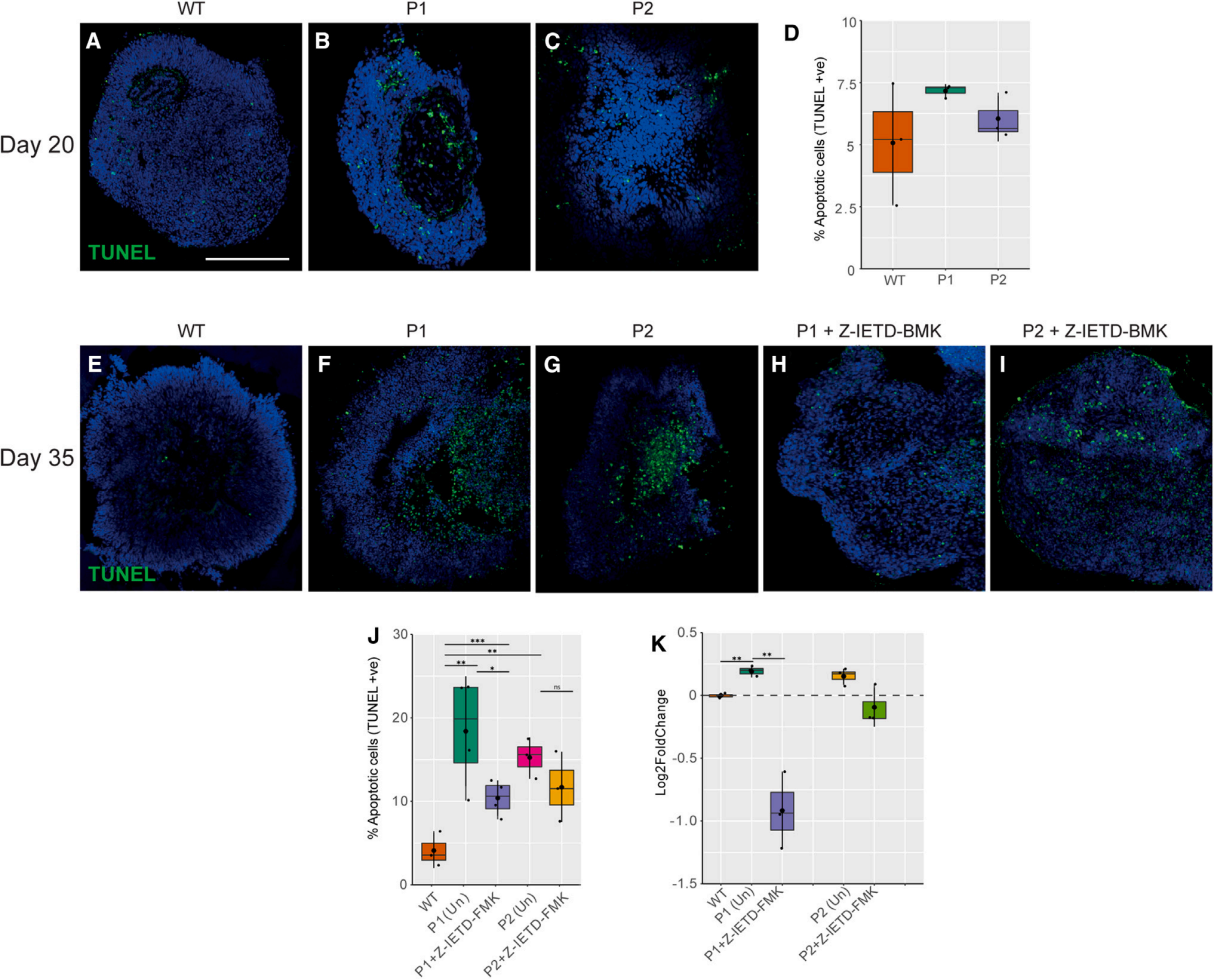
Apoptosis in microphthalmia optic vesicles (A–C) TUNEL+ cells detected in WT, P1, and P2 optic vesicles at day 20. (D) Quantification of apoptosis through TUNEL+ cells in WT, P1, and P2 optic vesicles at day 20. (E–G) TUNEL+ cells detected in WT, P1, and P2 optic vesicles at day 35. (H and I) TUNEL+ cells detected in Z-IETD-FMK-treated P1 and P2 optic vesicles at day 35. (J) Quantification of apoptosis through TUNEL+ cells in WT and treated and untreated P1 and P2 optic vesicles at day 35. (K) Quantification of caspase-8 activity in WT and treated and untreated P1 and P2 optic vesicles by firefly caspase-8 assay. Experiments were performed with *n* = 3 from two clones per condition. *p* < 0.05 (^∗^), *p* < 0.01 (^∗∗^), *p* < 0.001 (^∗∗∗^). All results are expressed as mean ± SD.

To measure cell death, apoptosis in OVs was quantified using the TUNEL assay. At day 20, 5.07% ± 2.45% of cells were TUNEL+ in WT OVs (Figure 7A). This increased, but not significantly, in P1 to 7.17% ± 0.27% apoptotic cells (*p* < 0.27, *n* = 3) and in P2 to 6.05% ± 0.91% (*p* < 0.553, *n* = 3) (Figures 7A–7D). However, by day 35, significant changes in apoptosis were detected in WT compared to microphthalmia OVs. The proportion of TUNEL+ cells was significantly higher in both P1 and P2 at 18.40% ± 6.58% (*p* < 0.00233, *n* = 3) and 15.24% ± 2.41% (*p* < 0.00707, *n* = 3), respectively, than 4.10% ± 2.10% of cells in WT OVs (Figures 7E–7I).

### Treatment with caspase-8 inhibitor Z-IETD-FMK reduced apoptosis in microphthalmic OVs

Due to increased apoptosis, the question of whether pharmacological modulation of apoptosis pathways could reduce cell death in patient OVs was examined. RNA-seq data highlighted significant upregulation of *CASP8*, an initiator of the caspase-mediated apoptosis pathway, which acted as a candidate therapeutic target. Apoptosis was reduced in microphthalmia vesicles treated with caspase-8 inhibitor Z-IETD-FMK, as the proportion of TUNEL+ cells in P1 significantly decreased to 10.39% ± 2.10% (*p* < 0.0000688, *n* = 3) and in P2 to 11.69% ± 4.18%, but this was not significant (*p* < 0.271, *n* = 3) (Figure 7J).

CASP8 activity was quantified using a colorimetric assay to quantify apoptosis in WT, treated and untreated patient OVs. CASP8 levels in untreated WTs were established as the baseline for normalization. CASP8 activity was significantly upregulated in P1 and P2 OVs compared to WT (1.14-fold [*p* < 0.00828, *n* = 3] and 1.11-fold [*p* < 0.040, *n* = 3], respectively) (Figure 7K). Treatment with caspase-8 inhibitor Z-IETD-FMK significantly reduced caspase-8 activity in P1 vesicles 0.52-fold compared to untreated vesicles (*p* < 0.0219, *n* = 3) (Figure 7K). Although treatment of P2 vesicles with Z-IETD-FMK reduced caspase-8 activity in P2 vesicles 0.94-fold, this was not found to be significant (*p* < 0.944, *n* = 3) (Figure 7K).

## Discussion

In this study, novel shared disease mechanisms which contribute to human microphthalmia using patient-derived induced pluripotent stem cell (iPSC) OVs were described. A greater understanding of these mechanisms is critical for developing potential therapies and identifying molecular markers that may improve diagnostic rates. The overproduction of ECM components, including LUM, NID2, and COL4A1 that encapsulate and infiltrate the OV, may potentially physically limit ocular growth and development, resulting in eye malformations such as microphthalmia. Dysregulation of early ocular, apoptosis-, and cell proliferation-associated genes resulted in OVs with significantly reduced diameters, increased cell death and reduced cell proliferation contributing to the “small eye” phenotype. Furthermore, treatment with caspase-8 inhibitor Z-IETD-FMK significantly attenuated apoptosis in one patient (P1), suggesting targeting cell death pathways as a potential prophylactic therapy.

### Pathogenic variants disrupt GRNs controlling early eye development

The reduced diameters of microphthalmia patient OVs in our study recapitulate the human phenotype. Further work is required to understand vesicle growth dynamics as vesicle diameter did not increase significantly between day 35 and 50. Previous studies of *VSX2*-associated microphthalmia also reported reduced diameters of patient OVs coupled with reduced cell proliferation (Phillips et al., 2014). Gamm et al. posited that changes to the expression of EFTFs such as VSX2 may influence cell proliferation and vesicle diameter as these changes were only observed following the onset of VSX2 expression at ∼ day 30 (Phillips et al., 2014). Similarly, we only observed changes to cell proliferation in our patient vesicles from day 35, strengthening the hypothesis that the onset of these defects coincides with changes to VSX2 expression driving neural retina (NR) formation in the optic cup. Both our data and previous studies show dysregulation of early ocular GRNs due to reduced expression of early ocular genes such as *RAX*, *HES5*, *SIX6*, and *LHX5*. In contrast, *LHX5* was downregulated in our microphthalmia patients but upregulated in *VSX2*-associated microphthalmia (Phillips et al., 2014). Animal studies showed that loss of *LHX1* and *LHX5* impaired OV formation as cells acquired a pigment-like fate rather than NR (Inoue et al., 2013). VSX2 was also shown to bind to an intergenic region adjacent to *LHX5* to regulate its expression in early eye development but not to *LHX1* (Capowski et al., 2016). LHX1 and LHX5 may act as cell fate-determining transcription factors in a larger GRN that is critical in NR formation (Inoue et al., 2013). Our data showed significant loss of *LHX5* expression at both day 20 and 35 but of *LHX1* only at day 35. This is in accordance with previous data that showed LHX5 induces *LHX1* expression, which is required for OV patterning from the forebrain (Inoue et al., 2013). This regulatory relationship where *LHX1* becomes transcriptionally active downstream to LHX5 activity has also been observed in cortical development (Miquelajáuregui et al., 2010). Synthesizing our data and previous work, we can suggest a purported role for LHX5 in early development that when disrupted, may contribute to ocular malformation as clinically observed in our patients. Although the role of VSX2 in optic cup morphogenesis has been widely reported, this study may further support the essential role of LHX5 and LHX1 in human early eye development as VSX2 does not act independently to determine the NR fate of progenitors (Gamm et al., 2019). Alternatively, the pathways involved in early ocular development may be modulated in a mutation-dependent manner which culminates in a common downstream disease pathway. *MITF* expression is upregulated in both datasets, further strengthening the notion that the acquisition of a more RPE-like rather than NR cell fate is a common abnormality in microphthalmia patients and multiple genes may act in this pathway (Phillips et al., 2014).

Although not directly related to early ocular GRNs, mitochondrial disease caused by variants in *HCCS*, *COX7B*, and *NDUFB11* has also been previously associated with MAC cases (Indrieri and Franco, 2021; Wimplinger et al., 2007; Eintracht et al., 2020b). Anomalous mitochondrial gene expression in microphthalmia OVs may indicate mitochondrial dysfunction contributes to ocular malformation. Previous studies have suggested mitochondrial dysfunction may underlie some developmental disorders due to mitochondrial involvement in early neuroectodermal differentiation (Zhu et al., 2016; Indrieri and Franco, 2021). This study introduces mitochondrial changes as a possible shared disease mechanism in MAC but further research is required to investigate mitochondrial dysfunction as an underlying shared etiology.

While the disruption of GRNs involved in early eye development has been well characterized, the downstream changes to cellular function that underlie disease pathophysiology are still largely unknown (Eintracht et al., 2020a). GO term enrichment analysis revealed global changes in cell processes such as signal transduction, cell adhesion, cell-cell communication, and differentiation, but further work is required to broaden our understanding of how genetic mutations disrupt these cellular functions in microphthalmia.

### Upregulation of ECM components may overly constrict optic cup morphogenesis resulting in ocular malformation

Critical ECM components were upregulated in microphthalmia vesicles at day 20 and 35. The signaling and mechanical properties of the ECM that guide optic cup morphogenesis have been widely described in early eye development (Bryan et al., 2016, 2020; Eamegdool et al., 2020; Oltean et al., 2016; Casey et al., 2021). Research suggests *LUM* variants and polymorphisms are potentially associated with human myopia, playing a role in binding collagen fibrils and constricting their diameter to regulate scleral size (Lyu et al., 2021; Amjadi et al., 2013; Wang et al., 2017). As collagen fibrils have been shown to regulate scleral development, LUM action may control eye globe size during development (Coudrillier et al., 2015). In both *Lum*^*−/−*^ zebrafish (antisense oligonucleotide morpholino gene knockdown) and stable transgenic mouse myopia models, the loss of ECM components increased axial length and resulted in myopia. The morpholinos were injected into fertilized eggs and a phenotype was first observed at 22 hours post fertilization (hpf) and still observed by 6 dpf (Yeh et al., 2010). By 6 dpf, the morpholino would not be effective in the zebrafish, suggesting that initial knockdown of LUM may have a more long-term impact, for example through autoregulation. Alternatively, the loss of LUM at early developmental stages may disrupt the spatiotemporal GRNs in the early eye, where the loss of a gene at its critical spatiotemporal expression window is irrecoverable and results in irreversible ocular malformation.

Involvement of the retinal basement membrane (RBM) in regulating eye size has been well documented in previous studies, diverting our attention to the composition of RBM ECM proteins (Halfter et al., 2006, 2015). Although only detected in human fetal tissue from 9 weeks post conception, it has been identified in 24 hpf zebrafish and E4 stage chicks, suggesting involvement in early eye development (Balasubramani et al., 2010; Bryan et al., 2020). NID2 has a critical role in the formation of the RBM, a structure comprising layers of ECM that anchor the developing neuroepithelium of the NR to drive ocular development by the action of physical forces (Miosge et al., 2001; Bryan et al., 2020). As a major component of the RBM, NID2 was also found to have the most interactions with other BM ECM proteins, binding collagen type IV, and laminin to create subcomplexes that aggregate to form the BM critical for eye morphogenesis (Balasubramani et al., 2010). Previous studies showed that the loss of NID2 significantly impaired optic cup morphogenesis as it likely regulates cell movement and RPE flattening in the developing optic cup (Bryan et al., 2020). Loss of nidogen has also shown to cause coloboma in *foxd3;tfap2a* mutants by disrupting epithelial cell migration during morphogenesis (Yoon et al., 2020; Bryan et al., 2020). Furthermore, mutations in *NID1*, an important paralog of *NID2*, have also been directly associated with ocular malformations, suggesting an essential role for these proteins in eye development (Trejo-Reveles et al., 2023). Mass spectrometry also revealed novel interactions between nidogen and LUM in the brain ECM, where altered expression levels of both proteins disrupted brain ECM composition (Rodrigues-Amorim et al., 2022). It is possible that similar interactions exist in the developing eye, such that dysregulated ECM homeostasis results in disrupted ECM organization and function.

COL4A1 and COL4A4 are other components of the RBM that provide tensile strength and play a critical role in oculogenesis (Halfter et al., 2015; Eamegdool et al., 2020). Studies into fetal eye development reported upregulated collagen type IV expression and disrupted expression patterns in microphthalmia eyes, in accordance with our data (Sijilmassi et al., 2021). While associated with human MAC phenotypes, there is a lack of data describing the role of collagen in eye morphogenesis and its interactions (Deml et al., 2014; Matías-Pérez et al., 2018). This study aims to shed further light on the role of the ECM with an emphasis on collagen, LUM, and nidogen in particular. It is suggested that a denser ECM contributes to coloboma as its failed displacement obstructs optic fissure fusion (Chan et al., 2020). This cannot be tested in OVs as they do not contain optic fissure; hence, further work is required in alternative disease models, such as the chick or zebrafish, to characterize the interaction and cooperation of ECM organization and function in the developing eye. Indeed, *nid1* expression was detected in both the chick and zebrafish optic fissure margin (OFM) (Trejo-Reveles et al., 2023; Knickmeyer et al., 2018). The conserved expression in the OFM across species and the coloboma phenotype observed in *nid1* morphant zebrafish suggest an essential role for nidogen in optic fissure closure and further investigation of human orthologs *NID1* and *NID2* as novel candidate coloboma genes (Knickmeyer et al., 2018; Trejo-Reveles et al., 2023).

The upregulation of LUM and other ECM proteins such as NID2 and COL4A1 in microphthalmia patient OVs in contrast to the loss of Lumican in animal models of increased axial length may be indicative of an inversely proportional relationship between the ECM and axial length (Eiraku et al., 2012). Oltean et al. (2016) demonstrated that the ECM is critical to optic cup invagination as it provides mechanical forces that constrain the OV, initiating the invagination process (Oltean et al., 2016). *LUM* has been associated with conferring resistance and structure to the ECM through collagen organization, suggesting overexpression may generate excessive tensile force on the developing eye (Rodrigues-Amorim et al., 2022). Our data highlight the upregulation of multiple key ECM components; the overexpression of RBM proteins such as NID2 and COL4A1 may create large subcomplexes that aggregate to create an abnormally large BM in microphthalmic patients. This hypothesis is supported from zebrafish models that showed mechanical cues provided by the ECM are critical for organogenesis, suggesting molecular signals and genetic gradients alone are not sufficient to drive development (Monnot et al., 2022).

This study is the first to map the expression patterns of NID2, LUM, and COL4A1 in stem cell-derived OVs and show their association in microphthalmia. The *in vitro* localization of ECM components identified in this study largely mirror their *in vivo* localization, such as NID2 expression was localized to the basal aspect of the OV, similar to basal deposits of nidogen detected in the developing zebrafish neuroretina and RPE or the more ubiquitous expression of COL4A1 in the mouse eye similar to the patterns we observed *in vitro* (Bryan et al., 2020; Sijilmassi et al., 2021). However, due to the complex microenvironment of the developing OV *in vivo* and its interactions with surrounding tissues, the expression patterns of these ECM proteins *in vitro* will not identically mimic those *in vivo*. Further work is required to assess the role of these ECM proteins in both expanded patient cohorts and *in vivo* models of microphthalmia.

### Cell proliferation and apoptosis may contribute to the microphthalmia phenotype

Genomic instability through pathogenic variants can contribute to changes in the cell cycle, inhibiting proliferation and increasing apoptosis (Matos-Rodrigues and Martins, 2021; Matos-Rodrigues et al., 2020; French et al., 2013). Overexpression of *Cdkn2b* in *Xenopus* microphthalmia models correlated with reduced cell proliferation due to cell cycle misregulation (Aldiri et al., 2013), and upregulation of *CDKN2A* and *CDKN2B* reduces proliferation in the developing retina (Dimaras et al., 2008). These genes are negative cell cycle regulators that transition cells from the G1 to S phase and are effectors of the TGF-β pathway-mediated cell cycle inhibition that reduces proliferation (Hannon and Beach, 1994; Chaum et al., 2015; Teotia et al., 2017; Xia et al., 2021; Soto et al., 2005). At day 20 and 35, *CDKN2A* and *CDKN2B* were upregulated in both microphthalmia patients, as were TGF-β-related genes at day 20. Although cell proliferation levels did not significantly differ between WT and patient vesicles at day 20, transcriptional irregularities may precede such changes, as *CDKN2A* and *CDKN2B* both target CDK genes that regulate transcription factors active in the G1/S phase transition (Xia et al., 2021). These targets induce positive and negative feedback loops that are temporally spaced to facilitate correct progression of the cell cycle, suggesting a possible delay in effect (Eser et al., 2011; Georgi et al., 2002). Interestingly, reduced expression of *Cdkn2a* in TASP1-deficient mice significantly decreased cranial and ocular malformations, including microphthalmia, suggesting inhibition of these genes may be required for correct retinal progenitor proliferation to drive ocular morphogenesis (Takeda et al., 2015). Our data showed *SIX6* was downregulated alongside other critical ocular genes such as *RAX*, *HES1*, and *PAX6* that temporally coincided with *CDKN2A* and *CDKN2B* upregulation, suggesting these genes may be regulated by similar GRNs that control early ocular gene expression (Gamm et al., 2019; Phillips et al., 2014; Matos-Rodrigues and Martins, 2021).

Apart from regulating cell proliferation, *CDKN2A* and *CDKN2B* also promote p53-dependent apoptosis and cell-cycle arrest in the retina and RPE previously associated with animal models of microphthalmia (Takeda et al., 2015; Chaum et al., 2015; Xia et al., 2021; Hannon and Beach, 1994; Rodrigues et al., 2013). In accordance, our RNA-seq data showed enrichment of the p53 pathway in the microphthalmia patient vesicles. Apoptosis has been described in animal models of microphthalmia, but not in human *in vitro* systems (Wu et al., 2003; Hettmann et al., 2000; Dash et al., 2020; Cheng et al., 2018; Capowski et al., 2016; Phillips et al., 2014). We identified global upregulation of upstream regulators of apoptosis, such as *XAF1*, *IRF1*, and *FZD9*, and caspase-recruiters inducing a caspase cascade, including CASP8, which drives apoptosis (Jeong et al., 2018)*.* FZD9 is a receptor for Wnt ligands in Wnt signaling and its haploinsufficiency has been previously associated with increased apoptosis in neural progenitor cells (Chailangkarn and Muotri, 2017; Chailangkarn et al., 2016). Our data show reduced expression of *FZD9* and *FZD10* and further Wnt pathway misregulation, suggesting Wnt abnormalities may contribute to the increased apoptosis observed in the retinal progenitor cells. Abnormal Wnt signaling has also been identified in other cell death pathways such as necrosis or ferroptosis and the TNF death receptor pathway (Li et al., 2019; Wang et al., 2022; Hiyama et al., 2013), where dysfunctional signaling observed in patient OVs may promote cell death through multiple pathways. Wnt signaling has been shown to be critical to early eye development and is involved in the regulation of cellular functions such as apoptosis, proliferation, differentiation, and tissue specification during morphogenesis (Fuhrmann, 2008; Cheng et al., 2018; Li et al., 2016).

This study is the first to describe upregulated caspase cascades associated with microphthalmia in humans although a purported role has been previously hypothesized (Wimplinger et al., 2007). Caspase effectors of apoptosis were not upregulated in microphthalmia patients in our study. It is possible that apoptosis is effected through other signaling pathways activated by CASP8 such as the TNF death receptor pathway as reflected in our transcriptomics data (Tummers and Green, 2017). Further evidence indicates this is a common pathway in microphthalmia from the *VSX2*-associated microphthalmia dataset. Although no caspases were upregulated in their dataset, Phillips et al. reported upregulation at day 30 of *TNFRSF19*, *TNFRSF21*, and *TNFRSF10D*, all effectors of the activation of the CASP8-TNF death receptor apoptosis pathway (Phillips et al., 2014; Sakamaki and Satou, 2009). Some discrepancies between datasets may exist as our data measure gene expression levels at day 35 while Phillips et al. measured them at day 30. Our data combined with previous studies of microphthalmia may suggest apoptosis modulated through CASP8 and the TNF death receptor is a shared pathway contributing to ocular malformation, as alternative cell death pathways are likely involved in developmental eye disorders (Gregory-Evans et al., 2011).

### Therapeutic interventions reduce apoptosis in microphthalmia patient OVs

This study highlighted the therapeutic potential of inhibiting aberrant cell death. The caspase inhibitor Z-IETD-FMK showed significant reduction in apoptosis in P1; however, future work should involve a larger sample size. Pharmacological inhibition of apoptosis pathways has previously been shown to be effective in zebrafish models *gup* and *pax2.1* models of coloboma (Gregory-Evans et al., 2011). However, the effects on phenotype between these models differed greatly, as the coloboma phenotype was only corrected in the *gup* model (Gregory-Evans et al., 2011). Expanding on this work, Lusk and Kwan (2022) found that *pax2a*, but not *pax2b*, influences cell survival in the optic fissure and *pax2a* mutants displayed increased apoptosis and coloboma (Lusk and Kwan, 2022). Inhibition of apoptosis with anti-apoptotic factor *Bcl-xl* RNA did partially rescue the coloboma phenotype in these mutant zebrafish (Lusk and Kwan, 2022). These studies show apoptosis has a clear role in developmental eye disorders, particularly the MAC phenotype, which can also be targeted for rescue. In this study, apoptosis was only significantly reduced in P1 with a trend reduction in P2. The differing response to the drug may have been due to uptake in OVs as P1 vesicles displayed enhanced structural integrity, lamination, and self-organization of VSX2+ cells compared to P2. A previous study identified an enhanced photoreceptor-specific response to drugs modulating apoptosis, and this effect may be mirrored in retinal progenitor cells (Dorgau et al., 2022). It is also possible that the more severe microphthalmia phenotype (and underlying genetic basis) of P2 requires a higher dose of Z-IETD-FMK to significantly influence apoptosis pathways.

However, it is unlikely to be an effective treatment for patients due to the need for *in utero* administration at a very early stage and the challenge of detecting MAC phenotypes at this critical time point (Smets et al., 2006; Lindsay et al., 2016; Mysore et al., 2019). Curcumin, a natural polyphenol with antiapoptotic activity, has been suggested as a prophylactic therapy for mothers at increased risk i.e., with a positive family history, but further work is required to assess its safety and efficacy in humans. For those who have a genetic diagnosis, family planning advice should be sought as options including preimplantation genetic diagnosis may be applicable (Treff et al., 2020). For microphthalmia, depending on phenotypic severity, a post-natal therapeutic approach which permits continued growth and maturation of the eye with preservation or improvement in residual vision may be more amenable.

### Limitations of the study

As ECM genes are upregulated *in vitro* in the absence of the microenvironment of the developing eye and the extrinsic cues that induce structural changes in the OV such as those arising from the surface ectoderm, there is possible overlap between ECM regulation and the GRNs controlling early eye development. This also may explain why ECM overproduction is common between microphthalmia patients with differing genotypes. This hypothesis is supported from previous studies into *VSX2-*associated microphthalmia. In both our patients and *VSX2*-associated microphthalmia, we observed common upregulation of ECM components such as collagen and laminin family proteins, fibronectin, and hyaluronan and proteoglycan-link proteins that play significant roles in eye development (Phillips et al., 2014; Casey et al., 2021; Turksen et al., 1985). The temporal discrepancies in upregulation of ECM genes between microphthalmia patients may suggest mutation-specific downstream effects within a common molecular architecture. Our data support this hypothesis as LUM expression was significantly upregulated at day 20 in P1 but at day 35 in P2. Further work is required to investigate ECM production in microphthalmia patients with variants affecting different cellular processes or that represent nodes in different GRNs to fully understand the common nature of ECM overproduction in microphthalmia. However, it is also possible that the upregulation of MITF observed in microphthalmia patient OVs may promote ECM production due to an imbalance between the presumptive NR and RPE (Eamegdool et al., 2020). Therefore, changes to ECM production may be a secondary event of ocular malformation, not a primary cause driving aberrant eye morphogenesis. This study is limited as it has not investigated the spatial and temporal changes to the ECM *in vivo* to provide further evidence to either hypothesis.

Retinal differentiation of human iPSCs (hiPSCs) is highly variable in efficiency between individual cell lines and differentiation protocols (Capowski et al., 2019; Cowan et al., 2020). Sample sizes are often small due to the intense financial and labor costs in generating retinal organoids as well as inherent inter- and intra-sample variability (Afanasyeva et al., 2021). While some markers may predict retinal differentiation efficiency such as *Meis1* (Wang et al., 2018), a complete picture of the differentiation capacity of each clone of a hiPSC line is only possible following intensive clonal screenings that are time, labor, and cost intensive (Cowan et al., 2020). It is possible that not all variability between hiPSC lines, clones, and differentiation rounds can be controlled for and may influence the data.

The genetic background of each generated cell line will influence its differentiation capacity, whether through heterogeneous gene expression patterns or epigenetic “memory” regulating gene expression (Kim et al., 2010; Lewis and Kats, 2021). Indeed, modeling of human disease using patient-specific organoids revealed large transcriptional heterogeneity between patients resulting in diverse transcriptomic profiles that may alter cell fate decisions *in vitro* (Krieger et al., 2021; Chamling et al., 2021). The creation of isogenic lines eliminates these concerns as all hiPSC lines are generated on the same background, enabling any abnormalities to be specifically associated with the pathogenic variant. The strength of the conclusions made by this study is limited by the lack of isogenic lines. Furthermore, this study would be improved by investigating these shared mechanisms in a larger cohort of genotyped microphthalmia patients.

## Experimental procedures

### Resource availability

#### Lead contact

All relevant data supporting the key findings of this study are available within the article and its supplemental information files. Further information and requests can be made to the corresponding author, Mariya Moosajee (m.moosajee@ucl.ac.uk).

#### Materials availability

There were no new reagents generated for this study.

#### Data and code availability

All RNA sequencing data generated in this study have been deposited in the NCBI’s Gene Expression Omnibus database (GEO Series Accession Number: GSE265940).

### Methods

#### Ethics and consent

This study complied with all tenets of the Declaration of Helsinki, This study falls under ethics 11/LO/243 NRES study of congenital eye disease under the National Research Ethics Service from Moorfields Eye Hospital NHS Foundation Trust. Written informed consent for publication of the participants’ details and/or their images was obtained from the participants.

#### hiPSC derivation

iPSCs were derived from human dermal fibroblasts obtained from WTs and patients, with informed consent, and characterized for pluripotency markers and absence of chromosomal anomalies, as described in detail in Méjécase et al. (2020), Harding et al. (2021), Méjécase et al. (2020), and Harding et al. (2021).

#### OV differentiation

Embryoid bodies (EBs) were formed as previously described (Eintracht et al., 2022), and differentiation was performed as outlined (Mellough et al., 2015; Chichagova et al., 2019). 48 h after EB formation (day 2), EBs were plated by gentle pipetting into 60 mm TC-treated culture dishes. Cells were cultured in neural induction media, (DMEM/F12, knockout serum residue [KOSR], 2% B27, 1x non-essential amino acids (NEAA), 1% P/S, 1xGlutaMAX, and 5 ng/mL IGF-1) with decreasing KOSR concentrations: 20% from day 2–7, 15% from day 7–11, and 10% from day 11–18. From day 18, cells were cultured in retinal differentiation media (DMEM/F12, 10% FBS, 2% B27, 1xNEAA, 1xGlutaMAX, 1% P/S, 5 ng/mL IGF-1, 0.1 mM taurine, 40 ng/mL triiodothyronine, and 0.5 μM retinoic acid).

#### Statistics

Statistical analysis was performed using Excel and Prism (Microsoft, USA; GraphPad, USA). A two-tailed unpaired Student’s t test was used for comparison studies for diameter, pH3, and TUNEL quantitation; however, a two-tailed paired Student’s t test was used for comparison studies between conditions for western blotting as individual rounds of differentiation are compared. A *p* value of <0.05 was considered statistically significant. Significance levels were set when *p* < 0.05 (^∗^), *p* < 0.01 (^∗∗^), *p* < 0.001 (^∗∗∗^). All results are expressed as mean ± SD unless specified. All experiments were performed with *n* = 3 replicates grown at separate times in separate dishes.

A sample size of *n* = 3 was chosen as each sample is time, labor, and cost intensive to generate and cannot be equated to cell lines used for high-throughput experiments which are simpler and cheaper to maintain. This is commonly seen in the literature as shown in other studies investigating ocular development and disease, with some only using up to three clonal hiPSC lines per condition (Parfitt et al., 2016).

## Supplemental information

Document S1. Figures S1 and S2, Tables S1, S6, and S7, and supplemental experimental procedures

Table S2. Differentially expressed genes between the P v WT conditions at day 20

Table S3. Differentially expressed genes between the P v WT conditions at day 35

Table S4. Enriched gene ontology terms between the P v WT comparison at day 20

Table S5. Enriched gene ontology terms between the P v WT comparison at day 35

Document S2. Article plus supplemental information
