# Supplementary material for: Disruption of common ocular developmental pathways in patient-derived optic vesicle models of microphthalmia
Source: Stem Cell Reports. 2024 May 30;19(6):839–58. doi: 10.1016/j.stemcr.2024.05.001 (PMC11390689; doi:10.1016/j.stemcr.2024.05.001)
Supplement: Document S1. Figures S1 and S2, Tables S1, S6, and S7, and supplemental experimental procedures [file mmc1.pdf]

Stem Cell Reports, Volume *19*

Supplemental Information

Disruption of common ocular developmental pathways in patient-derived optic vesicle models of microphthalmia

Jonathan Eintracht, Nicholas Owen, Philippa Harding, and Mariya Moosajee

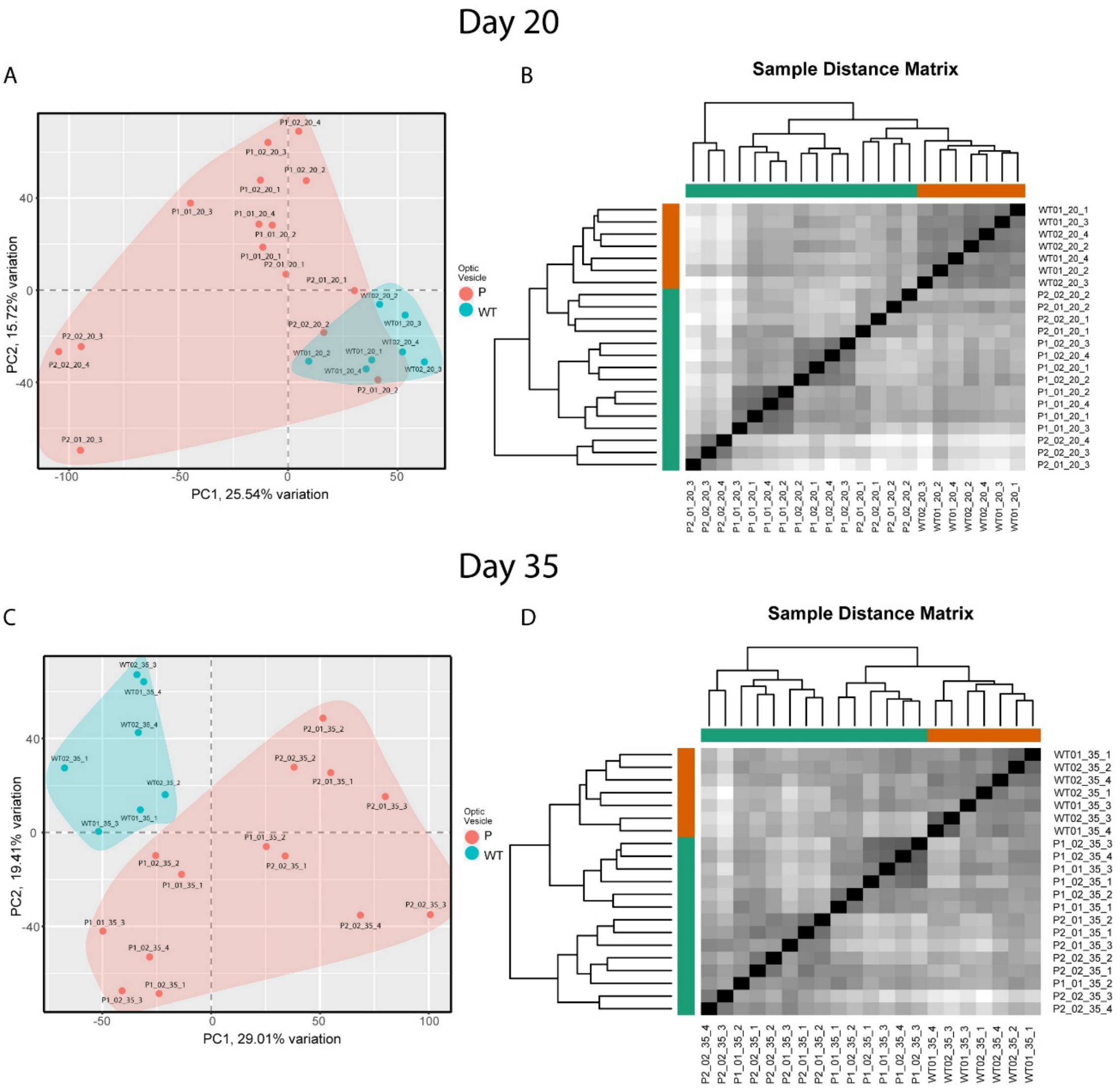

Supplementary Figure 1: Principal Component Analysis (PCA) and Sample Distance Matrix (SDM) analysis of P v WT vesicles at day 20 and day 35. (a-b) PCA and SDM showing separation of both P1 and P2 samples from wild type controls at day 20. Duplicate clones from each patient and wild type iPSC line are represented as either clone 01 or clone 02. (c-d) PCA and SDM showing separation of both P1 and P2 samples from wild type controls at day 35. Duplicate clones from each patient and wild type iPSC line are represented as either clone 01 or clone 02.

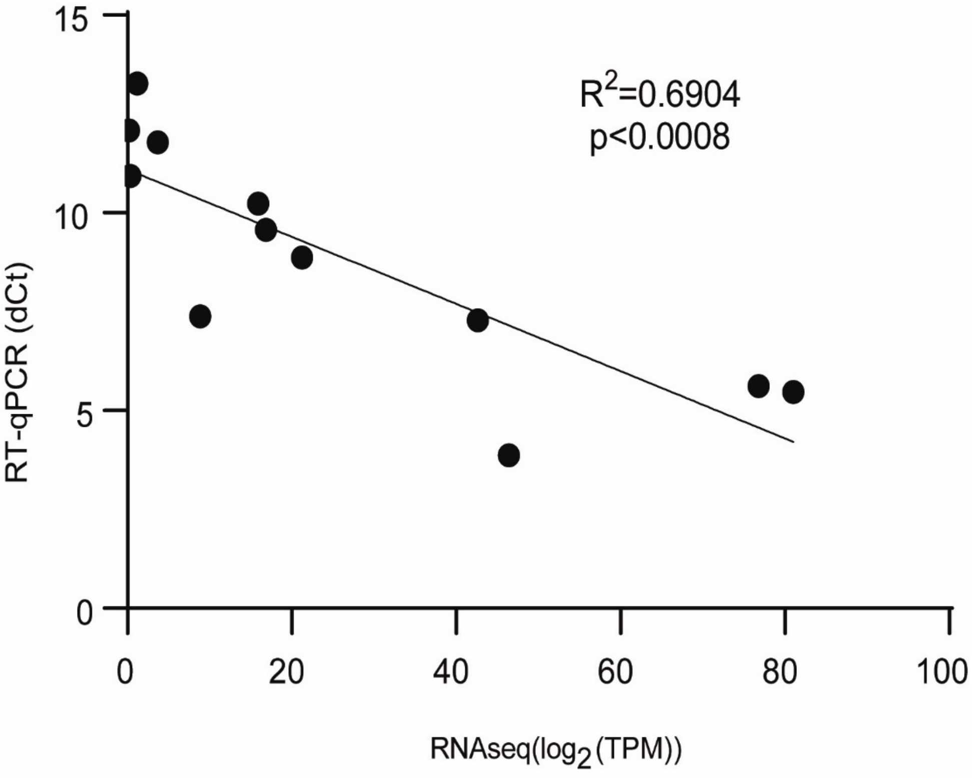

Supplementary Figure 2: Validation of robust control RNA-seq data with TPM data correlating to RT-qPCR gene expression. Scatterplot of optic vesicle sample RNA-seq normalised count data (TPM – transcripts per million) against normalised RT-qPCR ΔC_t values for 6 key ocular development transcription factors (*VSX2, MITF, RAX, PAX6, SOX2, SIX6*) at day 20 and 35 of differentiation with significant correlation (Spearman correlation R^2=0.6904, *p* value < 0.0008)

Table S1: Clinically observed patient phenotypes

	**Ocular Phenotype**	**Non-ocular phenotype**
**Patient 1 (P1)<br>16 year old male**	▪ Bilateral microphthalmia with axial length right eye 19.87 mm and left eye 20.03 mm<br>▪ Bilateral microcornea<br>▪ Bilateral iris and chorioretinal coloboma involving the macular and optic disc<br>▪ Best corrected visual acuity (BCVA) right eye no light perception and left eye 1.00 LogMAR.	N/A
**Patient 2 (P2)<br>35 year old female**	▪ Unilateral left microphthalmia with axial length right eye 23.1 mm and left eye 20.5 mm<br>▪ Right eye exhibited a complex phenotype of partial iris hypoplasia, microcornea, aphakic (following cataract surgery) with chorioretinal coloboma involving the optic disc<br>▪ Left eye microphthalmia with microcornea, cataract, no posterior view<br>▪ Best corrected visual acuity (BCVA) right eye 1.00 LogMAR and left eye no perception to light.	Type II diabetes mellitus<br>Obesity

Table S6 – Primer Sequences

Primer	Sequence (5'-3')	Product size (bp)
OCT4<br>RT-qPCR	F - CCCCAGGGCCCCATTTTGGTACC<br>R - ACCTCAGTTTGAATGCATGGGAGAGC	143
SOX2<br>RT-qPCR	F - TTCACATGTCCCA GCACTACCAGA<br>R - TCACATGTGTGAG AGGGGCAGTGTGC	80
LIN28<br>RT-qPCR	F - AGCCATATGGTAG CCTCATGTCCGC<br>R - TCAATTCTGTGCCT CCGGGAGCAGGG TAGG	129
c-MYC<br>RT-qPCR	F - GCGAACCCAAGAC CCAGGCCTGCTCC<br>R - CAGGGGGTCTGCT CGCACCGTGATG	143
GAPDH<br>RT-qPCR	F - ACAGTTGCCATGT AGACC<br>R - TTTTTGGTTGAGCA CAGG	95
OCT4 Plasmid<br>RT-qPCR	F - CATTCAAACTGAG GTAAGGG<br>R - TAGCGTAAAAGGA GCAACATAG	124
SOX2 Plasmid<br>RT-qPCR	F - TTCACATGTCCCA GCACTACCAGA<br>R - TTTGTTTGACAGGA GCGACAAT	111
c-MYC Plasmid<br>RT-qPCR	F - GGCTGAGAAGAGG ATGGCTAC<br>R - TTTGTTTGACAGGA GCGACAAT	122
LIN28 Plasmid<br>RT-qPCR	F - AGCCATATGGTAG CCTCATGTCCGC<br>R - TAGCGTAAAAGGA GCAACATAG	251
KLF4 Plasmid RT-<br>qPCR	F - CCACCTCGCCTTA CACATGAAGA<br>R - TAGCGTAAAAGGA GCAACATAG	156
EBNA Plasmid<br>RT-qPCR	F - ATCAGGGCCAAGA CATAGAGATG<br>R - GCCAATGCAACTT GGACGTT	61
PAX6<br>RT-qPCR	F - GGCCGAACAGACA CAGCCCTCAC<br>R - ATCATAACTCCGC CCATTCACC	165
OTX2<br>RT-qPCR	F - TAAAAATTGCTAGA GCAGCC<br>R - CATGGGAGGTTAG AAAAAGTC	248
RAX<br>RT-qPCR	F - AGGCGGAAAAATA GAGTTTG<br>R - TACCCCAATATTCA CTCCTC	139
LHX2<br>RT-qPCR	F - TTTTCTAATGACTC GCAACC<br>R - TTAGTTAGTTGCTC AAAGCC	108
VSX2<br>RT-qPCR	F - GGCGACACAGGAC AATCTTTA<br>R - TTCCGGCAGCTCC GTTTTC	122
MITF<br>RT-qPCR	F - CAGTACCTTTCTAC CACTTTAG<br>R - CCTCTTTTTCACAG TTGGAG	163
LUM<br>RT-qPCR	F – AAGGATTCAAACCATTTGCC<br>R -TCAATTTCAGCTCATCACAG	199

NR2F1<br>RT-qPCR	F - CCGGCGTGAATTATCCCGTA<br>R - GCTCTTTTTGTTGTGCCGGT	96
CASP8<br>RT-qPCR	F - CTACAGGGTCATGCTCTATC<br>R - ATTTGGAGATTTCCTCTTGC	92
LHX5<br>RT-qPCR	F - TTTCACCTCAACTGTTTCAC<br>R - TACAGGATGACACTGAGTTG	157

Table S7 – Primary and secondary antibodies

Primary Antibodies						
**Antibody**	**Species**	**Supplier**	**Catalogue #**	**RRID**	**Dilution**	**Application**
OCT4	Mouse	Santa Cruz Biotechnology	sc-5279	AB_628051	1:100	IF
SSEA3	Rat	Millipore	MAB4303	AB_177628	1:50	IF
AFP	Mouse	Santa Cruz Biotechnology	sc-51506	AB_626514	1:300	IF
Vimentin	Mouse	Santa Cruz Biotechnology	sc-6260	AB_628437	1:250	IF
PAX6	Rabbit	Covance	PRB-278P	AB_291612	1:100	IF
VSX2 (CHX10)	Mouse	Santa Cruz Biotechnology	sc-365519	AB_10842442	1:200 (IF), 1:1000(WB)	IF/WB
PH3	Rabbit	Cell Signaling Technology	9701	AB_1549592	1:500	IF
COL IV	Rabbit	Abcam	ab6586	AB_305584	1:100 (IF), 1:1000(WB)	IF/WB
LUM	Rabbit	Abcam	ab168348	AB_2920864	1:100 (IF), 1:1000(WB)	IF/WB
NID2	Rabbit	Abcam	ab14513	AB_301292	1:200 (IF), 1:1000(WB)	IF/WB
β-actin	Mouse	Sigma-Aldrich	A2228	AB_476697	1:5000	WB

Secondary Antibodies					
**Antibody**	**Species**	**Supplier**	**Catalogue #**	**RRID**	**Dilution**
Alexa Fluor® 488	Goat anti-Rat	Thermo Fisher Scientific	A-11006	AB_2534074	1:800
Alexa Fluor® 488	Goat anti-Rabbit	Thermo Fisher Scientific	A32731	AB_2633280	1:800
Alexa Fluor® 488	Donkey anti-Goat	Thermo Fisher Scientific	A-11055	AB_2534102	1:800

Alexa Fluor® 647	Donkey anti-Goat	Thermo Fisher Scientific	A-21447	AB_2535864	1:800
Alexa Fluor® 647	Goat anti-Mouse	Thermo Fisher Scientific	A-21235	AB_2535804	1:800
Polyclonal Goat Anti-Mouse Immunoglobulin antibody	Goat Anti-Mouse	Dako, Denmark	P0447	AB_2617137	1:10 000
Polyclonal Goat Anti-Rabbit Immunoglobulin antibody	Goat Anti-Rabbit	Dako, Denmark	P0448	AB_2617138	1:10 000

Supplementary Experimental Procedures

hiPSC derivation and culture

In brief, fibroblasts were derived from skin biopsies after overnight incubation and cultured 1×10^6 fibroblast cells were electroporated (1600 V, 20ms, 3 pulses) with 1 µg each of four episomal plasmids (pCXLE-hSK (Addgene ID# 27078), pCXLE-hUL (Addgene ID# 27080), pCXLE-hOCT3/4-shp53-F (Addgene ID# 27077) and pCXWB-EBNA1 (Addgene ID# 37624)) using the Neon Transfection System (Parfitt et al., 2016). Transfected cells were plated on 0.1% gelatin-coated 100 mm dishes in fibroblast media with 0.5 mM sodium butyrate (Sigma-Aldrich, cat#B5587). After seven days, cells were dissociated with TrypLE Express and 200,000 cells plated into each well of a Matrigel-coated (Corning, USA, cat#354377) 6-well plate in mTeSR Plus (STEMCELL Technologies, Canada, cat#1000276). Rudimentary iPSC colonies were excised from these plates and cultured in isolation. iPSCs were maintained in mTeSR Plus and passaged using ReleSR (STEMCELL Technologies, Canada, cat#05872).(Parfitt et al., 2016). Transfected cells were plated on 0.1% gelatin-coated 100 mm dishes in fibroblast media with 0.5 mM sodium butyrate (Sigma-Aldrich, cat#B5587). After seven days, cells were dissociated with TrypLE Express and 200,000 cells plated into each well of a Matrigel-coated (Corning, USA, cat#354377) 6-well plate in mTeSR Plus (STEMCELL Technologies, Canada, cat#1000276). Rudimentary iPSC colonies were excised from these plates and cultured in isolation. iPSCs were maintained in mTeSR Plus and passaged using ReleSR (STEMCELL Technologies, Canada, cat#05872).

Embryoid body formation

EBs were formed in Aggrewell™ plates, a plate where each well of a 24-well plate is comprised of 1200 microwells, as per manufacturer's instructions (STEMCELL Technologies, Canada, cat#34415). Briefly, iPSCs were washed with PBS and detached with Accumax (ThermoFisher Scientific, USA, cat#00-4666-56) to form a single cell suspension. After 5-8 minutes, mTeSR Plus media was added to each well. Cells were counted using Countess™ II Automated Cell Counter (ThermoFisher Scientific, USA). 3.6x106 cells per well were centrifuged and resuspended in 1mL mTeSR Plus with 10µM Y-27632 and added to one well of. the Aggrewell™ plate (3000 cells per microwell). Mixing with a pipette was required to ensure uniform distribution of cells. The plate was spun at 100g for three minutes and incubated at 37°C. After 24 hours, 1mL media was changed (day 1).

Optic vesicle differentiation

EBs were formed as previously described (Eintracht et al., 2022), and differentiation was performed as outlined (Mellough et al., 2015, Chichagova et al., 2019). 48 hours after EB formation (day 2), EBs were plated by gentle pipetting into 60mm TC-treated culture dishes (Appleton Woods, UK, cat#BF152) and cultured in Neural Induction Media (NIM), (DMEM/F12 (ThermoFisher Scientific, cat#31331028), 20% knock-out serum residue (KOSR) (ThermoFisher Scientific, cat#10828028, 2% B27

(ThermoFisher Scientific, USA, cat#17504001), 1xNon-Essential Amino Acids (NEAA; ThermoFisher Scientific, USA, cat#11140050), 1% P/S, 1xGlutamax (ThermoFisher Scientific, USA, cat#35050061) and 5ng/mL IGF-1 (Sigma-Aldrich, USA, cat#I3769). One well of an Aggrewell™ plate was transferred into six uncoated 60mm round dishes, resulting in a final density of approximately 200 EBs per 60mm culture dish, or 1200 EBs per $3.6x10^6$ cells.

Cells were cultured in NIM with decreasing KOSR concentrations, 20% from day 2-7, 15% from day 7-11 and 10% from day 11-18. From day 18, cells were cultured in Retinal Differentiation Media (DMEM/F12, 10% FBS, 2% B27, 1xNEAA, 1xGlutamax, 1% P/S, 5ng/mL IGF-1, 0.1mM taurine (Sigma-Aldrich, USA, cat#T8691), 40ng/mL triiodothyronine (Sigma-Aldrich, USA, cat#T6397) and 0.5µM retinoic acid (Sigma-Aldrich, USA, cat#R2625) added immediately before use. Cells were cultured in RDM until day 50. OV diameters were measured as described above.

Treatment with caspase-8 inhibitor Z-IETD-FMK

To prepare for cell culture applications, 1mg of caspase-8 inhibitor Z-IETD-FMK was reconstituted in 150µL DMSO to a concentration of stock concentration of 10mM. This was diluted to a working concentration of 40µM in cell culture media based on previous in vitro experiments (Shi et al., 2018, Monari et al., 2008, Yuan et al., 2018). OVs were treated with 40µM Z-IETD-FMK on day 33 and day 34, beginning 48 hours prior to harvest on day 35. The total concentration of DMSO in culture did not exceed 0.2% to prevent cytotoxic effects.

Optic vesicle diameter measurements

To measure OV diameters, the diameters of between 5-20 individual vesicles from brightfield images for each condition were measured using ImageJ (NIH, USA). Initially, diameters were measured in pixels using the ImageJ software by drawing a line across each vesicle and using the 'Measure' function. Lengths in pixels were converted to micrometres based on the manufacturers' data. For the 2x objective, the pixel size was 3.0854µm/pixel; for the 4x objective it was 1.5427µm/pixel and for the 10x objective it was 0.6172 µm/pixel.

RNA isolation, RT-qPCR and RNA-seq analysis

Each individual plate of OVs was collected at either day 0, day 20 or day 35 and RNA extraction was performed using the RNeasy Mini Kit (QIAGEN, Germany, cat#74104). cDNA was synthesized from 1µg using the SuperScript III First Strand cDNA synthesis kit (Invitrogen, USA, cat#18080093) according to manufacturer's instructions. RT-qPCR was performed using 2x SYBR Green Master Mix (ThermoFisher Scientific, USA, cat#4472908) as per manufacturer's instructions on the StepOne Real-Time PCR system (Applied Biosystems, ThermoFisher, UK). Primers used for the qRT-PCR at 200nM are listed below **(Table S6)** and were designed using the Primer-BLAST tool from the National Centre for Biotechnology Information. All transcript levels were measured in triplicate and normalised to

GAPDH, with undetermined C_t values in negative controls where no cDNA was present. The relative expression of each target gene compared to iPSCs at day 0 of differentiation was calculated using the comparative C_t or $2^{-\Delta\Delta Ct}$ method (Schmittgen and Livak, 2008). Statistical comparisons and figures were generated using GraphPad Prism (GraphPad Software, USA). The relative expression of each target gene compared to iPSCs at day 0 of differentiation was calculated using the comparative C_t or $2^{-\Delta\Delta Ct}$ method (Schmittgen and Livak, 2008). Statistical comparisons and figures were generated using GraphPad Prism (GraphPad Software, USA).

RNA-seq library construction was performed following the manufacturer's instructions using TruSeq stranded Total RNA with Ribo Zero Plus rRNA depletion (Illumina cat 20020599). Subsequent libraries were run on a bioanalyzer to verify size and concentration prior to sequencing on the NovaSeq 6000 platform at Novogen, UK. Raw sequence reads were assessed for quality issues and adapter sequences, or other sources of contamination using fastqc (https://www.bioinformatics.babraham.ac.uk/projects/fastqc/). Low quality sequences with PHRED quality scores below 6 were trimmed from reads using trim galore (v0.6.6, https://www.bioinformatics.babraham.ac.uk/projects/trim_galore/). Reads passing these quality filters were aligned using HISAT2 (v2.2.1) to the Ensembl reference genome build GRCh38 and annotation version 102 (obtained Feb 2021) (Kim et al., 2019). Gene count tables were generated for all samples using featurecounts from the subread package (v2.0.0), and differential expression analysis carried out with DESeq2 v1.34 (Love et al., 2014). Genes with an absolute log2 fold change of ≥1 and an FDR ≤ 0.05 were classified as differentially expressed. Further downstream analysis, including gene ontology (GO) over representation analysis, gene set enrichment analysis (GSEA), data visualisation and statistical tests were conducted using R for statistics v4.1.2. Initially P1 and 2 data were grouped for pairwise comparison with WT, subsequent analysis investigated all possible pairwise comparisons. Functional classifications of DEGs was analysed by GO ORA using gProfiler and by Gene Set Enrichment Analysis (GSEA) Bioconductor packages GAGE (Luo et al., 2009) and fgsea (Korotkevich et al., 2021) (Subramanian et al., 2005) using unfiltered ranked gene list. Molecular Signature Databases were obtained from MsigDB (v7.4), including pathways with curated gene sets from online pathway databases, biomedical literature and contributions from domain experts. This collection comprised of KEGG (http://www.genome.jp/kegg/pathway.html), Reactome (http://www.reactome.org/), Gene Ontologies (BP/MF/CC), Canonical Pathways, and WikiPathways.

Western Blotting

Samples were analysed by western blotting as previously described (Eintracht et al., 2021). Briefly, cells were washed with ice-cold PBS and total protein extract was prepared with RIPA buffer (ThermoFisher Scientific, MA, USA, cat#89900) at a ratio of $5x10^6$ cells/mL buffer and 1 x Halt™ protease and phosphatase inhibitor cocktail (ThermoFisher Scientific, MA, USA, cat#78440).

Proteins were resolved on 4–15% Mini-PROTEAN® TGX ™ gels (Bio-Rad Inc., CA, USA, cat#4561025) and transferred to an Immun-Blot™ PVDF membrane using a Trans-Blot® SD semi-dry transfer cell (Bio-Rad Inc., CA, USA). Membranes were blocked with 5% non-fat dry milk in 0.1% PBS/T for 1 h. A complete list of primary and secondary antibodies and appropriate dilutions can be found in **Table S7**. Blots were scanned using the ChemiDoc XRS ™ Imaging System (Bio-Rad Inc., CA, USA) and quantitatively analysed using the Fiji/ImageJ software (National Institutes of Health, MD, USA).

Embedding and cryoembedding of vesicles

OVs were fixed in 4% paraformaldehyde (Fisher Scientific, UK, cat# for 10-20 minutes at 4°C, washed three times with PBS and stored overnight at 4°C in 30% sucrose (Sigma-Aldrich, USA, cat#S0389). Vesicles were embedded individually in 1.5cm x 1.5cm x 0.5cm moulds (Fisher Scientific, UK, cat#11670990) containing 800µL 7.5% gelatin/10% sucrose (Sigma-Aldrich, USA cat#G2500) solution and left to solidify at 4°C overnight. Embedded vesicles were excised from their moulds and placed in OCT embedding media (Agar Scientific, UK, cat#AGR1180) prior to snap-freezing in -50°C 2-methylbutane using a small dewar in a fumehood (Sigma-Aldrich, USA, cat#M32631) for three minutes. Frozen blocks were stored at -80°C.

Immunohistochemistry

Vesicles were sectioned using the Leica CM 3050 S cryostat at a thickness of 10µm and slides were left at RT for 1-2 hrs. Slides were washed twice for five minutes in PBS+0.1%Tween®20 (PBS/T) (Sigma-Aldrich, USA, cat#P1379) and permeabilized in PBS/T+0.5% Triton-X (Fisher Scientific, UK, cat#10591461) for one hour at RT with slight agitation. Samples were washed with PBS/T for five minutes and then blocked for one hour at RT in PBS+0.2% gelatin+0.5% Triton-X. Samples were incubated with primary antibodies overnight at 4°C (primary antibodies and dilutions are listed below in **Table S7**). Samples were subsequently washed three times for ten minutes with PBS/T. Samples were then incubated with secondary antibodies for one hour at RT in the dark (secondary antibodies and dilutions are listed below in **Table S7**). Samples were washed again three times for five minutes with PBS/T and DAPI (Sigma Aldrich, UK, cat#D9542) and once for five minutes with PBS. Slides were dipped in 100% ethanol and left to dry at RT. Once dry, coverslips were mounted with ProLong™ Diamond Antifade Mountant (ThermoFisher Scientific, USA, cat#P36961) and left to set overnight at RT in the dark. Slides were imaged using the confocal microscopes ZEISS LSM 700 and LSM 710 (ZEISS Research, Germany) and figures were generated using ImageJ (NCBI, USA) and Adobe Illustrator (Adobe Inc, USA).

TUNEL Assay

Four or five OVs from each condition were fixed, cryoembedded and sectioned as described above. Apopotic cell death was detected using the ApopTag Fluorescein *In Situ* Apoptosis Detection Kit (Merck Life Sciences, UK, cat#S7110) as per manufacturer's instructions. Sections were washed with DAPI and sealed with ProLong™ Diamond Antifade Mountant as described above. Slides were imaged as described above and stacked images collated on ImageJ (NCBI, USA). Levels of apoptotic cell death were quantified using the cell counter plugin on ImageJ (NCBI, USA).

Caspase-8 Colorimetric Assay

OVs from all conditions were collected and snap frozen at day 35. Protein was extracted and caspase-8 activity was measured using the Caspase-8 Colorimetric Assay Kit (R&D Systems, USA, cat#K113-100), as per manufacturer's conditions. Absorbance was measured using the EMax Plate Reader (Molecular Devices, USA). Data analysis was performed by Microsoft Excel (Microsoft, USA) and figures generated using GraphPad Prism (GraphPad Software, USA).

References

CHICHAGOVA, V., DORGAU, B., FELEMBAN, M., GEORGIOU, M., ARMSTRONG, L. & LAKO, M. 2019. Differentiation of Retinal Organoids from Human Pluripotent Stem Cells. *Current Protocols in Stem Cell Biology,* 50**,** e95.

EINTRACHT, J., FORSYTHE, E., MAY-SIMERA, H. & MOOSAJEE, M. 2021. Translational readthrough of ciliopathy genes BBS2 and ALMS1 restores protein, ciliogenesis and function in patient fibroblasts. *EBioMedicine,* 70**,** 103515.

EINTRACHT, J., HARDING, P., CUNHA, D. L. & MOOSAJEE, M. 2022. Efficient embryoid-based method to improve generation of optic vesicles from human induced pluripotent stem cells. *F1000Research,* 11**,** 324.

KIM, D., PAGGI, J. M., PARK, C., BENNETT, C. & SALZBERG, S. L. 2019. Graph-based genome alignment and genotyping with HISAT2 and HISAT-genotype. *Nature biotechnology,* 37**,** 907-915.

KOROTKEVICH, G., SUKHOV, V., BUDIN, N., SHPAK, B., ARTYOMOV, M. N. & SERGUSHICHEV, A. 2021. Fast gene set enrichment analysis. *BioRxiv***,** 060012.

LOVE, M. I., HUBER, W. & ANDERS, S. 2014. Moderated estimation of fold change and dispersion for RNA-seq data with DESeq2. *Genome biology,* 15**,** 1-21.

LUO, W., FRIEDMAN, M. S., SHEDDEN, K., HANKENSON, K. D. & WOOLF, P. J. 2009. GAGE: generally applicable gene set enrichment for pathway analysis. *BMC bioinformatics,* 10**,** 1-17.

MELLOUGH, C. B., COLLIN, J., KHAZIM, M., WHITE, K., SERNAGOR, E., STEEL, D. H. W. & LAKO, M. 2015. IGF-1 Signaling Plays an Important Role in the Formation of Three-Dimensional Laminated Neural Retina and Other Ocular Structures From Human Embryonic Stem Cells. *Stem Cells (Dayton, Ohio),* 33**,** 2416-2430.

MONARI, C., PAGANELLI, F., BISTONI, F., KOZEL, T. R. & VECCHIARELLI, A. 2008. Capsular polysaccharide induction of apoptosis by intrinsic and extrinsic mechanisms. *Cellular microbiology,* 10**,** 2129-2137.

PARFITT, DAVID A., LANE, A., RAMSDEN, CONOR M., CARR, A.-JAYNE F., MUNRO, PETER M., JOVANOVIC, K., SCHWARZ, N., KANUGA, N., MUTHIAH, MANICKAM N., HULL, S., GALLO, J.-M., DA CRUZ, L., MOORE, ANTHONY T., HARDCASTLE, ALISON J., COFFEY, PETER J. &

CHEETHAM, MICHAEL E. 2016. Identification and Correction of Mechanisms Underlying Inherited Blindness in Human iPSC-Derived Optic Cups. *Cell Stem Cell,* 18**,** 769-781.

SCHMITTGEN, T. D. & LIVAK, K. J. 2008. Analyzing real-time PCR data by the comparative C T method. *Nature protocols,* 3**,** 1101.

SHI, G., JIA, P., CHEN, H., BAO, L., FENG, F. & TANG, H. 2018. Necroptosis occurs in osteoblasts during tumor necrosis factor-α stimulation and caspase-8 inhibition. *Brazilian Journal of Medical and Biological Research,* 52.

SUBRAMANIAN, A., TAMAYO, P., MOOTHA, V. K., MUKHERJEE, S., EBERT, B. L., GILLETTE, M. A., PAULOVICH, A., POMEROY, S. L., GOLUB, T. R. & LANDER, E. S. 2005. Gene set enrichment analysis: a knowledge-based approach for interpreting genome-wide expression profiles. *Proceedings of the National Academy of Sciences,* 102**,** 15545-15550.

YUAN, Y., ZHANG, Y., ZHAO, S., CHEN, J., YANG, J., WANG, T., ZOU, H., WANG, Y., GU, J., LIU, X., BIAN, J. & LIU, Z. 2018. Cadmium-induced apoptosis in neuronal cells is mediated by Fas/FasL-mediated mitochondrial apoptotic signaling pathway. *Scientific Reports,* 8**,** 8837.
