# Supplementary material for: Disruption of common ocular developmental pathways in patient-derived optic vesicle models of microphthalmia
Source: Stem Cell Reports. 2024 May 30;19(6):839–58. doi: 10.1016/j.stemcr.2024.05.001 (PMC11390689; doi:10.1016/j.stemcr.2024.05.001)
Supplement: Document S2. Article plus supplemental information [file mmc6.pdf]

Stem Cell Reports
Article
OPEN ACCESS

Disruption of common ocular developmental pathways in patient-derived optic vesicle models of microphthalmia

Jonathan Eintracht,[1] Nicholas Owen,[1] Philippa Harding,[1] and Mariya Moosajee[1,2,3,*]
[1]UCL Institute of Ophthalmology, London EC1V 9EL, UK
[2]Moorfields Eye Hospital NHS Foundation Trust, London EC1V 9EL, UK
[3]Francis Crick Institute, London NW1 1AT, UK
*Correspondence: m.moosajee@ucl.ac.uk
https://doi.org/10.1016/j.stemcr.2024.05.001

SUMMARY

Genetic perturbations influencing early eye development can result in microphthalmia, anophthalmia, and coloboma (MAC). Over 100 genes are associated with MAC, but little is known about common disease mechanisms. In this study, we generated induced pluripotent stem cell (iPSC)-derived optic vesicles (OVs) from two unrelated microphthalmia patients and healthy controls. At day 20, 35, and 50, microphthalmia patient OV diameters were significantly smaller, recapitulating the "small eye" phenotype. RNA sequencing (RNA-seq) analysis revealed upregulation of apoptosis-initiating and extracellular matrix (ECM) genes at day 20 and 35. Western blot and immunohistochemistry revealed increased expression of lumican, nidogen, and collagen type IV, suggesting ECM overproduction. Increased apoptosis was observed in microphthalmia OVs with reduced phospho-histone 3 (pH3+) cells confirming decreased cell proliferation at day 35. Pharmacological inhibition of caspase-8 activity with Z-IETD-FMK decreased apoptosis in one patient model, highlighting a potential therapeutic approach. These data reveal shared pathophysiological mechanisms contributing to a microphthalmia phenotype.

INTRODUCTION

Eye morphogenesis is tightly regulated by highly conserved gene regulatory networks (GRNs) of transcription factors such as *PAX6*, *MITF*, *RAX*, and *SOX2* that drive eye-field formation in the anterior neural plate (Eintracht et al., 2020a; Mic et al., 2004; Steinfeld et al., 2013).

Disruptions to these GRNs can result in the clinical spectrum of structural eye malformations including microphthalmia, anophthalmia, and ocular coloboma (collectively termed MAC) (Eintracht et al., 2020a). Microphthalmia is defined as a small, underdeveloped eye (axial length of <19 mm at 1 year of age or <21 mm in adulthood); anophthalmia is the absence of an eye; and ocular coloboma is a failure of optic fissure fusion, resulting in a persistent inferonasal tissue defect within the eye (Eintracht et al., 2020a). There can be significant overlap with a mixed clinical phenotype, but they can also appear in isolation or with other systemic features (Eintracht et al., 2020b). Despite over 100 genes being associated with MAC, a recent study suggested only 33% of patients receive a molecular diagnosis while other studies estimate a lower positive molecular diagnosis rate at between 20% and 30% (Harding et al., 2022; Basharat et al., 2023; Plaisancié et al., 2019). The majority of solved cases arise from variants in eye-field transcription factors (EFTFs) such as *SOX2*, *OTX2*, and *PAX6* (Eintracht et al., 2020b; Williamson and FitzPatrick, 2014). Other pathogenic variants affect pathways including retinoic acid, bone morphogenetic protein (BMP), and transforming growth factor β (TGF-β) signaling that have key roles in oculogenesis (Williamson and FitzPatrick, 2014; Deml et al., 2014). Although the disruptive effects of specific genes on early eye development have been extensively characterized, there remains no comparative studies describing shared or common disease pathways causing ocular malformation independent of genotype (Gamm et al., 2019; Capowski et al., 2016; Phillips et al., 2014; den Hollander et al., 2010; French et al., 2013; Williamson et al., 2020; Wu et al., 2003).

The correct spatiotemporal expression of cell cycle and proliferation genes is critical for eye growth (Asaoka et al., 2014; Green et al., 2003; Yamaguchi et al., 2005). Pathogenic variants that directly influence the expression of such genes may alter the dynamics of cell division and death in the developing eye, resulting in microphthalmia (Phillips et al., 2014; French et al., 2013; den Hollander et al., 2010; Xu et al., 2007; Kim et al., 2016). For instance, apoptosis was increased by an *Msx2* variant that activated the caspase-3/8 pathway and by the loss of *Rbm* that abolished Birc2 inhibition of anti-apoptotic pathways in mouse *Msx2* models of microphthalmia (Yu et al., 2018). Additionally, upregulation of some pro-apoptotic and cell cycle regulatory genes has been described in *in vitro* microphthalmia studies showing reduced optic vesicle (OV) size in microphthalmia patient-derived models (Capowski et al., 2016; Phillips et al., 2014).

Alongside the intrinsic molecular cues that guide ocular development, extrinsic cues generate both the biophysical conditions driving optic cup morphogenesis and the extracellular signals to create a unique microenvironment for the developing eye (Bryan et al., 2020; Casey et al., 2021; Serjanov et al., 2018). Contact between the surface

This is an open access article under the CC BY license (http://creativecommons.org/licenses/by/4.0/).

ectoderm and the neuroectodermal OV is mediated through the extracellular matrix (ECM) secreted by both tissues (Lee e t al., 2021). The ECM has a critical role in eye development due to the physical limits placed on lateral OV growth that drives optic cup invagination (Oltean et al., 2016). Retinal progenitor proliferation and differentiation are also modulated by the ECM via cues that alter cell cycle dynamics (Serjanov et al., 2018).

Disruptions to laminin sub-units α, β, and γ have resulted in ocular malformations via loss of basement membrane (BM) integrity, cell polarity, or focal adhesion assembly (Serjanov et al., 2018; Bryan et al., 2016; Guo et al., 2019). The ECM protein nidogen secreted by the BM has a critical role in zebrafish oculogenesis as its disruption impaired optic cup formation (Bryan et al., 2020; Carrara et al., 2019). Exogenous nidogen protein supplemented to *tfap2a;foxd3* double-mutant zebrafish that lacked neural crest cells, ablating the BM, led to partial rescue of defective optic cup invagination and optic fissure closure (Bryan et al., 2020). The ECM component lumican (*LUM*) has a role in axial length control as $Lum^{-/-}/Fmod^{-/-}$ double knockout mice and *lum*-morpholino zebrafish all had significantly elongated axial lengths compared to wild type (WT) at 8 weeks of age and 22 days post fertilization (dpf), respectively (Yeh et al., 2010; Song et al., 2016). Additionally, collagen type IV variants or expression level changes have been associated with ocular maldevelopment, while *COL4A1/4* regulates retinal pigment epithelium (RPE) growth and function *in vitro* (Deml et al., 2014; Eamegdool et al., 2020; Bai et al., 2009; Sijilmassi et al., 2021; Matías-Pérez et al., 2018). This suggests a critical role for the ECM in early ocular development, yet ECM dysfunction has yet to be described in microphthalmia.

In this study, we successfully identify shared morphological and molecular pathways between unrelated microphthalmia patients from different families, a bilateral microphthalmia patient who remained clinically unsolved following whole-genome sequencing and a unilateral microphthalmia patient with a *PAX6* heterozygous missense variant (c.372C>A) p.(Asn124Lys). Two common disease mechanisms were detected: (i) increased apoptosis, reduced proliferation, and cell cycle misregulation and (ii) overproduction of ECM components that may overly constrict eye growth resulting in microphthalmia. Our findings advance our understanding of GRNs controlling early eye development and the downstream effects of their disruption, while highlighting novel gene targets to improve future molecular diagnostics. This may also enhance our understanding of the large phenotypic heterogeneity associated with MAC and provide therapeutic targets for development to a larger cohort of patients without relying on a genetic diagnosis.

RESULTS

RNA-seq analysis revealed global transcriptomic changes to early ocular developmental pathways

To investigate shared pathophysiology between unrelated microphthalmia patients, bulk RNA sequencing (RNA-seq) was performed to create transcriptomic profiles of both healthy and patient-derived OVs. High-quality RNA was extracted from a WT line, patient one (P1, unsolved genetic diagnosis following whole-genome sequencing and full targeted gene panel screen of structural eye disease genes [Patel et al., 2019]), and patient two (P2, with *PAX6*-heterozygous missense (c.372C>A) p.(Asn124Lys) variant, confirmed pathogenic for microphthalmia) (Méjécase et al., 2020; Harding et al., 2021). Further clinical details of both patients can be found in Table S1. Days 20 and 35 were chosen as time points corresponding to OV and cup formation, respectively, as expression of ocular markers at these time points correlated to these milestones (Eintracht et al., 2022).

Analysis was carried out on two clones per line and four replicates per condition per time point. High-quality reads were mapped to human genome GRCh38, annotation version 102. At day 20, principal component analysis (PCA) demonstrated P1 samples clustered together while P2 shared some overlap with WT (Figure S1). At day 35, PCA of the data showed clustering of P1 and P2 together distinct from WT, suggesting some shared variance between microphthalmia patients compared to WT (Figure S1). At both day 20 and 35, principal component Pearson r^2 clinical correlates showed strong clustering of samples based on condition (Figure S1).

Groupwise and pairwise comparisons of all possible combinations were employed to identify differentially expressed genes (DEGs). DEGs were identified with a $\log_2$fold change (LFC) $\geq +1$ or ≤ -1 and a false discovery rate adjusted p value <0.05. Filtered DEG outputs for each comparison are presented in supplemental information (Tables S2 and S3). Initially, both patients were compared to WT in a three-way groupwise comparison to reveal any shared developmental deviations in early ocular morphogenesis. Additionally, patient samples were grouped together for pairwise analysis of patient P vs. WT conditions. Subsequently, results were confirmed with pairwise comparisons of each patient compared to WT and validated with quantitative reverse-transcription PCR (Figure S2). DEGs were reported in reference to WT in each comparison. At day 20, the P vs. WT comparison identified 1,865 DEGs in microphthalmia patients; 1,329 upregulated and 536 downregulated (Table S2). At day 20, transcriptomic changes to ocular development were evident as EFTFs

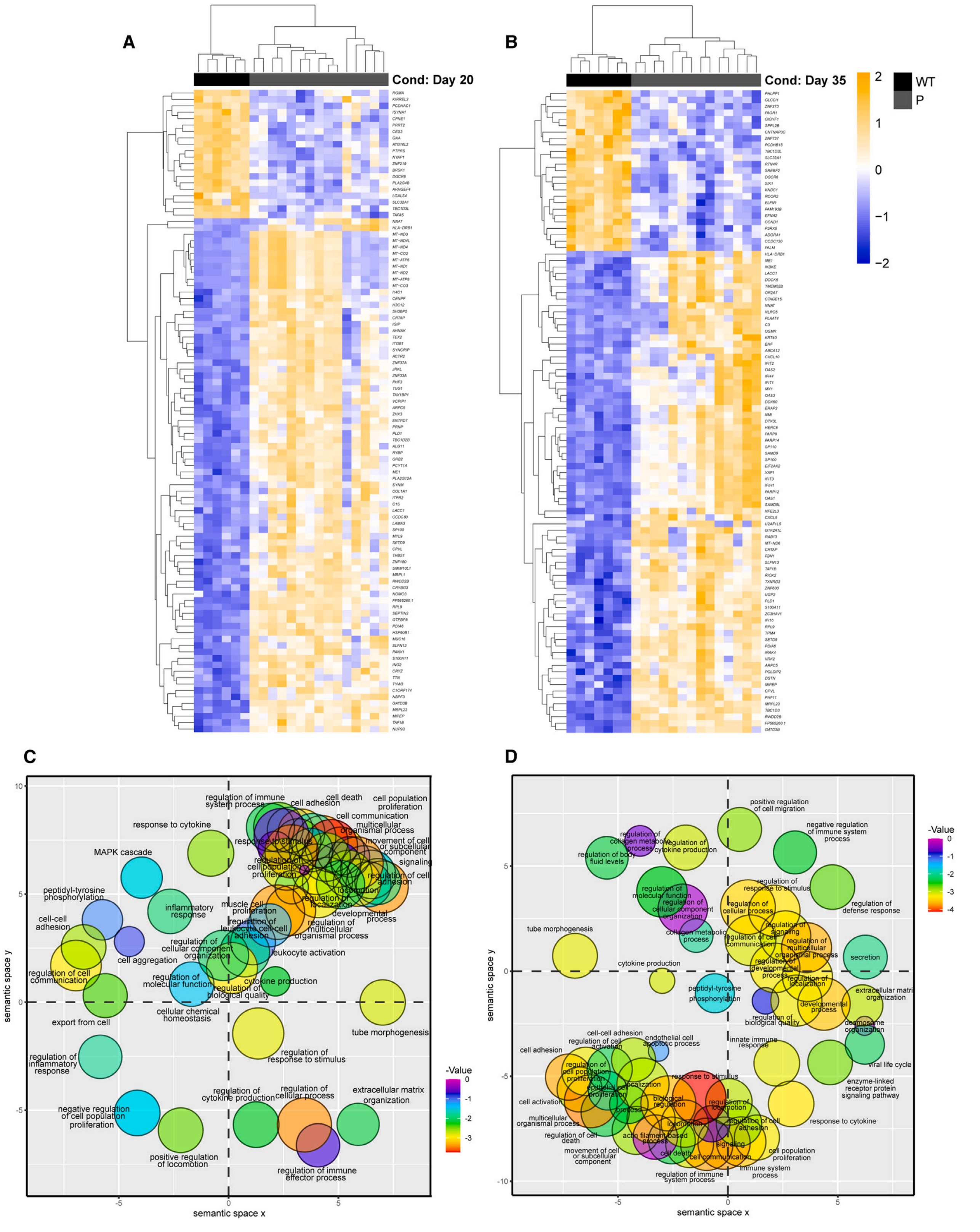

(legend on next page)

RAX (LFC −2.671, $p < 0.00247$), *PAX6* (LFC −1.191, $p < 0.0308$), *SIX6* (LFC −2.612, $p < 0.0000154$), *HES5* (LFC −2.03, $p < 0.0119$), and *LHX5* (LFC −2.420, $p < 0.0000326$) were downregulated while *GDF6* (LFC +1.674, $p < 0.000028$), part of the TGF-β family active in early development, and histone deacetylase *HDAC1* (LFC +1.239, $p < 0.000117$) that also plays a close role in progenitor cell proliferation and differentiation were upregulated (Figure 1A; Table S2). At the day 20 time point, upregulation of mitochondrial-related genes such as *MRPL3* (LFC +5.06, $p < 6.06 \times 10^{-14}$), *MT-ATP6* (LFC +4.65, $p < 1.30 \times 10^{-9}$), and *MT-ND2* (LFC +4.56, $p < 8.01 \times 10^{-10}$) was also observed (Figure 1A; Table S2). At day 35, the P vs. WT comparison identified 2,042 DEGs; 1,598 upregulated and 444 downregulated relative to WT (Table S3). At day 35, we measured downregulation of key ocular development genes such as *HES5* (LFC −1.651, $p < 0.00177$), *LHX1* (LFC −2.148, $p < 0.0000254$), and *LHX5* (LFC −2.117, $p < 0.0000008$) and upregulation of *MITF* (LFC +1.212, $p < 0.000420$) and *BMP4* (LFC +1.116, $p < 0.00329$) (Figure 1B; Table S3).

Unbiased hierarchical cluster analysis on significant DEG events at each time point identified gene co-regulation or functionally related DEGs at the identical time point and these data were visualized through heatmaps (Figures 1A and 1B). Individual clades were assigned general groups following visual examination of expression patterns. Gene Ontology (GO) enrichment analysis was performed on clades passing through data filters. At day 20, DEGs were enriched in many early developmental processes such as system development (GO:0048731), animal organ development (GO:0048513), and anatomical structure morphogenesis (GO:0009653) (Figure 1C; Table S4). Other enriched GO terms were globally affecting cell function such as multicellular organismal process (GO:0032501), biological adhesion (GO: 0022610), signaling (GO:0023502), and cell differentiation (GO:0030154).

At day 35, enriched GO terms were still related to early ocular development such as response to retinoic acid (GO:0032526), tissue development (GO:0009888), and regulation of anatomical structure morphogenesis (GO:0022603) (Figure 1D; Table S5). Other enriched GO terms included response to stimulus (GO:0050896), cell adhesion (GO:0007155), and cell surface receptor signaling pathway (GO:0007166).

RNA-seq analysis highlighted global upregulation of ECM-associated genes

GO term enrichment analysis at day 20 showed upregulated genes were enriched for ECM modeling such as extracellular matrix organization (GO:0030198), extracellular structure organization (GO:0043062), and extracellular matrix (GO:0031012). At day 35, ECM-related gene ontologies were the most significantly enriched in microphthalmia patients (Figure 2; Tables S4 and S5). This included enrichment in GO: cellular compartment structures such as collagen-containing extracellular matrix (GO:0062023) (Figure 2A) and GO: molecular function terms including extracellular matrix structural constituent conferring tensile strength (GO:0030020), (Figure 2B). We also observed enrichment in Kyoto Encyclopedia of Genes and Genomes (KEGG) pathway-related terms including ECM-receptor interaction (KEGG:04512) (Figure 2C), Panther-related terms such as plasminogen activating cascade, integrin signaling pathway, and cadherin signaling pathway that act in the ECM region (Figure 2D), and for Reactome terms, this included assembly of collagen fibrils and other multimeric structures (REAC:R-HSA-2022090), collagen formation (REAC:R-HSA-1474290), and extracellular matrix organization (REAC: R-HSA-1474244) and ECM proteoglycans (REAC:R-HSA-3000178) (Figure 2E). Additionally, GO:BP term analysis revealed enrichment of integrin-related terms such as integrin-mediated signaling pathway (GO:0007229) and integrin binding (GO:0005178).

Subsequent analysis aimed to identify specific ECM-associated genes upregulated in the patient OVs. At time points day 20 and 35, upregulation of ECM-associated genes such as *LUM* ([day 20 LFC +1.88, $p < 0.00280$] [day 35 LFC +3.12, $p < 3.70 \times 10^{-4}$]), *COL4A4* (day 35 LFC +1.47, $p < 3.70 \times 10^{-3}$), *LAMB4* ([day 20 LFC +2.11, $p < 3.69 \times 10^{-3}$] [day 35 LFC +2.05, $p < 4.00 \times 10^{-3}$]), *DSC3* ([day 20 LFC +2.12, $p < 3.99 \times 10^{-5}$] [day 35 LFC +2.04, $p < 2.41 \times 10^{-5}$]), and *NID2* ([day 20 LFC +1.12, $p < 1.00 \times 10^{-2}$] [day 35 LFC +1.35, $p < 1.51 \times 10^{-5}$]) was measured (Tables S2 and S3).

The extracellular matrix is enriched in microphthalmia patient OVs throughout early development

To further understand ECM overproduction in microphthalmia, LUM, COL4A1, and NID2 were selected for further analysis due to their specific role in eye development and

Figure 1. Transcriptomic profiling of microphthalmia optic vesicles
(A) Heatmap of top 100 differentially expressed genes between wild-type (WT) and patient (P) optic vesicles at day 20.
(B) Heatmap of top 100 differentially expressed genes between WT and patients at day 35.
(C) Enriched gene ontology (biological process) terms between WT and P at day 20.
(D) Enriched gene ontology (biological process) terms between WT and P at day 35. RNA-seq analysis was performed with $n = 4$ from two clones each per time point per condition.

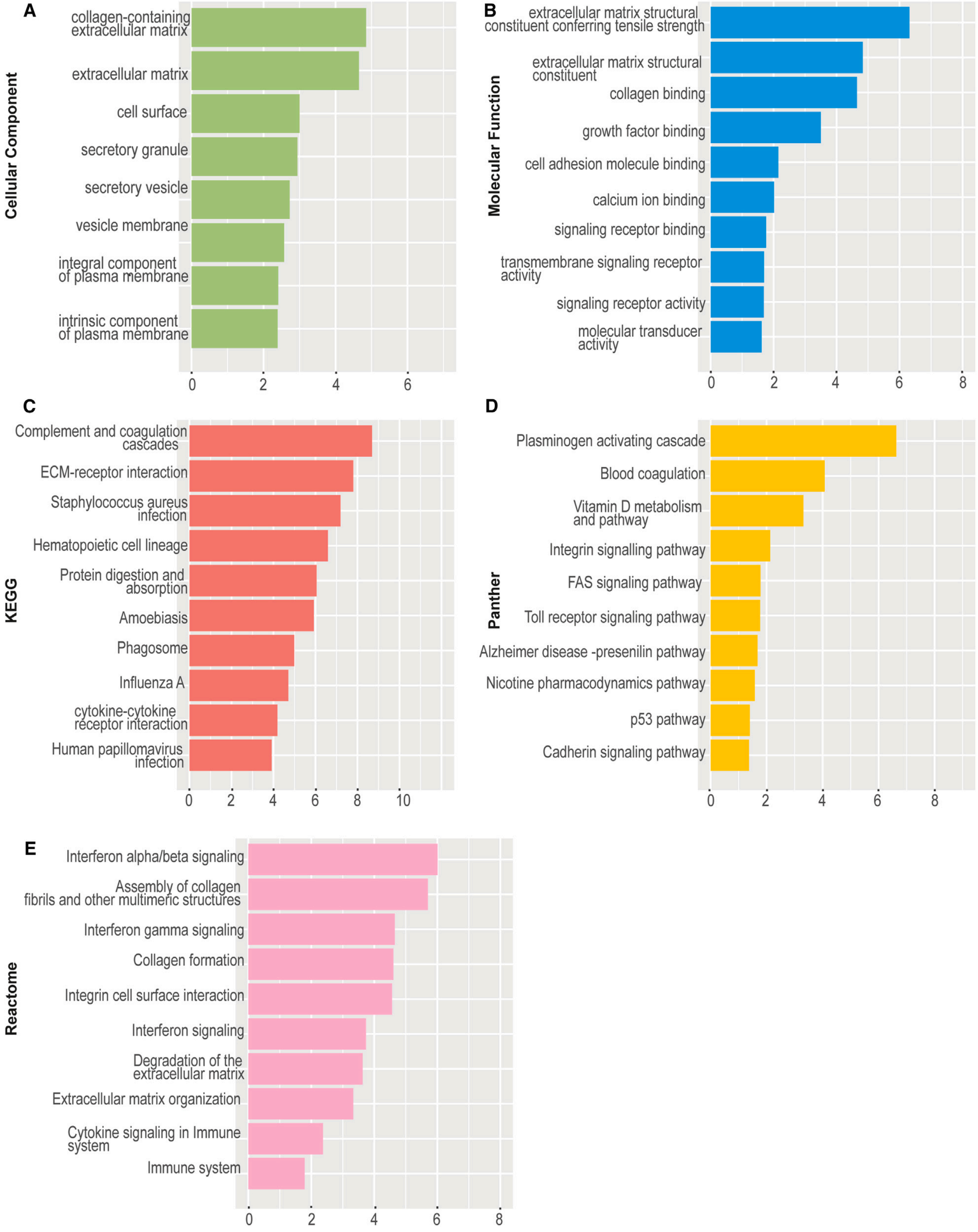

(legend on next page)

prior association with ocular maldevelopment (Bryan et al., 2020; Sijilmassi et al., 2021; Song et al., 2016; Yeh et al., 2010). We investigated whether LUM, COL4A1, and NID2 protein expression levels and patterns differed between WT and patient OVs. Western blot at day 20 highlighted significant upregulation of LUM 3.05-fold ($p < 0.0132$, $n = 3$) in P1 only, while nidogen was significantly upregulated in P2 only 2.99-fold ($p < 2.21 \times 10^{-3}$, $n = 3$) (Figures 3A and 3B). COL4A1 levels did not significantly differ between WTs and patients at day 20 ($p < 3.76 \times 10^{-1}$, $n = 3$; $p < 9.01 \times 10^{-1}$, $n = 3$) (Figures 3A and 3B). In WT, NID2 expression was confined to the basal layer of the OV; however, its expression was ubiquitous throughout patient OVs (Figures 3C–3E). LUM expression was confined to the apical aspect of both WT and patient OVs at day 20, yet expression intensity was higher in patients (Figures 3F–3H).

At day 35, NID2 was significantly upregulated 4.06-fold in P1 ($p < 0.0398$, $n = 3$) and 5.4-fold in P2 ($p < 0.0282$, $n = 3$) (Figures 4A and 4B). LUM was upregulated 1.5-fold in P1 and P2, yet this was only significant in P2 ($p < 0.0214 \times 10^{-2}$) (Figures 4A and 4B). Similar to NID2, COL4A1 expression was significantly upregulated in both P1 and P2 OVs 2.1-fold ($p < 0.0136$, $n = 3$) and 3.2-fold ($p < 0.0154$, $n = 3$), respectively (Figures 4A and 4B). NID2 expression was again confined to the basal aspect of WT OVs (Figures 4C–4E). NID2 was detected at the basal aspect of P1 OVs, yet in P2, NID2 was detected at the basal aspect and throughout the inner cell layers of the OV (Figures 4C–4E). LUM expression was strongest at the apical aspect of WT OVs (Figures 4F–4H). In P1, LUM was observed at the apical aspect of the OV, although it was detected throughout P2 OVs (Figures 4F–4H). COL4A1 expression was observed at the basal and apical aspects of WT OVs yet in both P1 and P2, COL4A1 expression was ubiquitous (Figures 4I–4K).

RNA-seq analysis revealed global upregulation of pro-apoptotic genes, changes to regulatory cell cycle, and proliferation-related genes

At day 20, pro-apoptotic genes such as *CARD16* (LFC +2.36, $p < 0.0105$), *CASP1* (LFC +5.53, $p < 0.00000713$), *CASP8* (LFC +1.65, $p < 0.000391$), and *XAF1* (LFC +2.33, $p < 0.00000777$) were upregulated. Frizzled receptors acting in the Wnt signaling pathway *FZD9* (LFC −1.28, $p < 0.000121$) (for which haploinsufficiency was previously associated with increased apoptosis in neural progenitor cells [Chailangkarn et al., 2016]), *FZD10* (LFC −2.34, $p < 0.0185$), and FZD9 interactor *APC2* (LFC −1.79, $p < 0.000583$) were downregulated. Wnt ligands and Wnt interactors were differentially expressed, suggesting major dysregulation of the Wnt signaling pathway associated with loss of frizzled receptor expression. Additionally, changes to cell cycle genes *CDKN2B* (LFC +2.10, $p < 0.0000609$), *CDKN2A* (LFC +2.68, $p < 0.00119$), and *CDK15* (LFC +1.55, $p < 0.00457$) were detected while TGF-β-related genes were also upregulated (Figure 1A; Table S2).

At day 35, more marked changes to expression levels of apoptosis genes were measured. Caspase recruiters *CARD6* (LFC +1.949, $p < 0.000179$), *CARD11* (LFC +1.89, $p < 0.0000553$), and *CARD16* (LFC +6.06, $p < 0.0000654$) were all significantly upregulated alongside caspases including *CASP1* (LFC +4.77, $p < 0.000000954$), *CASP4* (LFC +3.85, $p < 0.000000106$), and *CASP8* (LFC +2.46, $p < 0.0000038$). This global upregulation of pro-apoptotic genes was detected also in the tumor necrosis family (TNF) involved in the extrinsic apoptosis pathway (Figure 1B; Table S3).

Further upstream of the caspases in the apoptosis pathway, *XAF1* (LFC +5.26, $p < 0.0001$), *IRF1* (LFC +1.53, $p < 0.000341$), and *STAT1* (LFC +1.850, $p < 0.000000118$) and pro-apoptotic genes *BIRC3* (LFC +2.39, $p < 0.00000695$) and *BIK* (LFC +1.43, $p < 0.00178$) was upregulated in microphthalmia patients (Figure 1B; Table S3). At the day 20 time point, *FZD9* (LFC −1.22, $p < 0.0000484$) and *FZD10* (LFC −2.11, $p < 0.00228$) were downregulated. *APC2*, an FZD9 interactor, was also downregulated (LFC −1.61, $p < 0.00000559$). Upstream of the FZD9 receptor, Wnt ligands were differentially expressed, while Wnt inhibitory factor *WIF1* was also upregulated (LFC +1.05, $p < 0.0125$) (Figure 1B; Table S2).

DEGs involved in cell cycle regulation were also measured. Negative regulators of proliferation *CDKN2A* (LFC +2.10, $p < 0.00042$), *CDKN2B* (LFC +2.45, $p < 0.000000426$), and *JUNB* (LFC +1.69, $p < 0.000177$) were upregulated while cyclins *CCNP* (LFC −1.15, $p < 0.00163$) and *CCNA1* (LFC −1.28, $p < 0.00435$) were downregulated (Figure 1B; Table S2). The expression of cell cycle regulator *TMEM30B* (LFC +1.21, $p < 0.0231$) and FGF pathway genes regulating proliferation also differed between microphthalmia patients and WT (Figure 1B; Table S2).

Figure 2. Gene Ontology (GO) term enrichment analysis of WT vs. P (patient-derived) optic vesicles at day 35

(A) The highest enriched cellular compartment GO terms in WT vs. P at day 35 generated using RNA-seq data.

(B) The highest enriched molecular function GO terms in WT vs. P at day 35 generated using RNA-seq data.

(C) The highest enriched KEGG GO terms in WT vs. P at day 35 generated using RNA-seq data.

(D) The highest enriched Panther GO terms in WT vs. P at day 35 generated using RNA-seq data.

(E) The highest enriched Reactome GO terms in WT vs. P at day 35 generated using RNA-seq data. RNA-seq analysis was performed with $n = 4$ from two clones each per time point per condition.

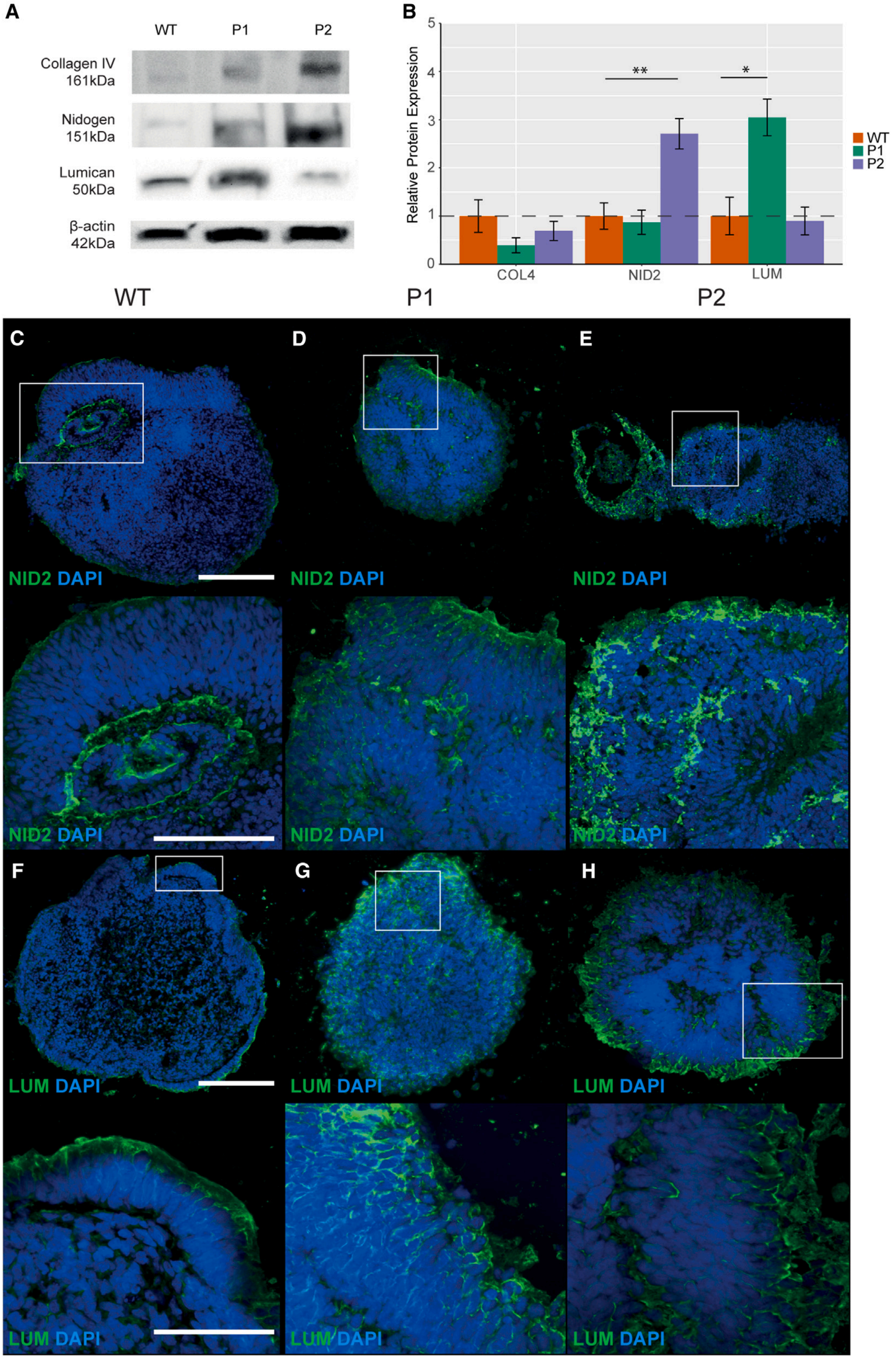

(legend on next page)

The DEGs at day 35 were enriched for proliferation GO terms including positive regulation of cell population proliferation (GO:0042127) and negative regulation of cell population proliferation (GO:0008285) (Figure 1D; Table S5). However, clusters of genes with higher expression in microphthalmia patients were primarily enriched in apoptosis-related terms including cell death (GO:0008219), programmed cell death (GO:0012501), apoptotic signaling pathway (GO:0097190), and extrinsic apoptotic signaling pathway (GO:0097191) (Figure 1D; Table S5).

Microphthalmia patient OVs exhibited reduced diameter and cell proliferation with increased apoptosis

The diameter of WT and patient OVs was measured at day 20, 35, and 50 to ascertain whether the "small eye" phenotype was recapitulated *in vitro*. At day 20, the mean diameter of WT OVs measured 513.10 ± 198.45 μm ($n = 20$) (Figures 5A–5D). This was significantly larger than both P1 at 397.64 ± 143.50 μm ($p < 0.0423$, $n = 20$) and P2 at 356.64 ± 96.82 μm ($p < 0.0038$, $n = 20$) (Figures 5A–5D). There were no significant differences between average diameters of P1 and P2 OVs ($p < 0.301$, $n = 20$) (Figure 5D).

At day 35, the mean diameter of WT OVs measured 741.65 ± 192.66 μm ($n = 20$) (Figure 5E). This was still significantly larger than both P1 at 629.04 ± 141.80 μm ($p < 0.0205$, $n = 20$) and P2 at 577.84 ± 132.62 μm ($p < 0.000869$, $n = 20$) (Figures 5E–5H). There were no significant differences between average diameters of P1 and P2 OVs ($p < 0.185$, $n = 20$) (Figure 5H).

At day 50, WT OVs had a mean diameter of 867.58 ± 282.71 μm that remained significantly larger than P1 at 671.66 ± 245.03 μm ($p < 0.0444$, $n = 20$) and P2 at 684.51 ± 166.41 μm ($p < 0.0401$, $n = 20$) (Figures 5I–5L). Similarly, the mean diameters of P1 and P2 OVs did not significantly differ ($p < 0.858$, $n = 20$) (Figure 5L). The mean diameters between day 35 and 50 WT OVs did not differ significantly ($p < 0.0980$, $n = 20$). OV diameters between day 35 and day 50 did not significantly differ in P1 ($p < 0.469$, $n = 20$), yet did significantly differ in P2 ($p < 0.0270$, $n = 20$).

Reduced OV diameter in microphthalmia patients may be a consequence of both reduced cell proliferation and increased cell death as highlighted from the RNA-seq analysis. To measure cell proliferation, OVs were analyzed by immunostaining to detect the presence of phospho-histone 3 (pH3) in cells currently undergoing mitosis. At day 20, no significant differences were detected between pH3+ cells in WT at 2.66% ± 0.61% of cells compared to P1 at 3.11% ± 0.14% ($p < 0.282$, $n = 3$) and P2 at 3.04% ± 1.34% ($p < 0.687$, $n = 3$) (Figures 6A–6D). At day 35, the proportion of pH3+ cells was significantly reduced in both P1 at 2.17% ± 0.40% of cells ($p < 0.0281$, $n = 3$) and P2 at 3.80% ± 0.70% of cells ($p < 0.0481$, $n = 3$) compared to WT OVs at 7.77% ± 1.81% of cells (Figures 6E–6H).

To measure cell death, apoptosis in OVs was quantified using the TUNEL assay. At day 20, 5.07% ± 2.45% of cells were TUNEL+ in WT OVs (Figure 7A). This increased, but not significantly, in P1 to 7.17% ± 0.27% apoptotic cells ($p < 0.27$, $n = 3$) and in P2 to 6.05% ± 0.91% ($p < 0.553$, $n = 3$) (Figures 7A–7D). However, by day 35, significant changes in apoptosis were detected in WT compared to microphthalmia OVs. The proportion of TUNEL+ cells was significantly higher in both P1 and P2 at 18.40% ± 6.58% ($p < 0.00233$, $n = 3$) and 15.24% ± 2.41% ($p < 0.00707$, $n = 3$), respectively, than 4.10% ± 2.10% of cells in WT OVs (Figures 7E–7I).

Treatment with caspase-8 inhibitor Z-IETD-FMK reduced apoptosis in microphthalmic OVs

Due to increased apoptosis, the question of whether pharmacological modulation of apoptosis pathways could reduce cell death in patient OVs was examined. RNA-seq data highlighted significant upregulation of *CASP8*, an initiator of the caspase-mediated apoptosis pathway, which acted as a candidate therapeutic target. Apoptosis was reduced in microphthalmia vesicles treated with caspase-8 inhibitor Z-IETD-FMK, as the proportion of TUNEL+ cells in P1 significantly decreased to 10.39% ± 2.10% ($p < 0.0000688$, $n = 3$) and in P2 to 11.69% ± 4.18%, but this was not significant ($p < 0.271$, $n = 3$) (Figure 7J).

CASP8 activity was quantified using a colorimetric assay to quantify apoptosis in WT, treated and untreated patient OVs. CASP8 levels in untreated WTs were established as the baseline for normalization. CASP8 activity was significantly upregulated in P1 and P2 OVs compared to WT (1.14-fold [$p < 0.00828$, $n = 3$] and 1.11-fold [$p < 0.040$, $n = 3$], respectively) (Figure 7K). Treatment with caspase-8 inhibitor Z-IETD-FMK significantly reduced caspase-8

Figure 3. Expression of ECM proteins in day 20 optic vesicles

(A) Western blot showing expression levels of COL4A1, NID2, and LUM in WT, P1 (patient 1), and P2 (patient 2) optic vesicles at day 20. β-Actin was used as a loading control for normalization of expression levels.

(B) Quantification of COL4A1, NID2, and LUM expression levels from the western blot.

(C–E) Expression patterns of NID2 in WT, P1, and P2 optic vesicles at day 20.

(F–H) Expression patterns of LUM in WT, P1, and P2 optic vesicles at day 20. Experiments were performed with $n = 3$ from one clone per condition. $p < 0.05$ (*), $p < 0.01$ (**), $p < 0.001$ (***). All results are expressed as mean ± SD.

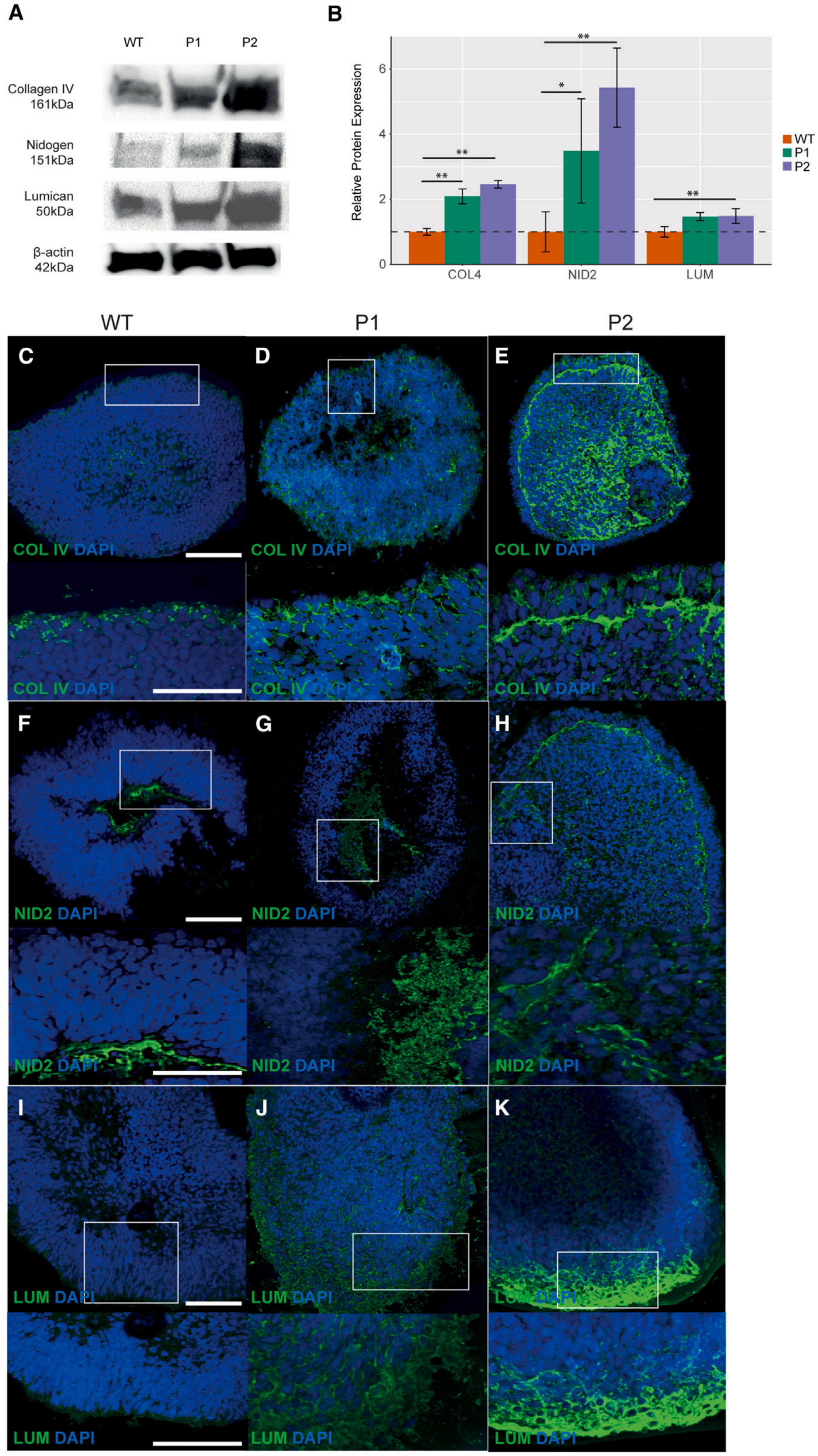

(legend on next page)

activity in P1 vesicles 0.52-fold compared to untreated vesicles ($p < 0.0219$, $n = 3$) (Figure 7K). Although treatment of P2 vesicles with Z-IETD-FMK reduced caspase-8 activity in P2 vesicles 0.94-fold, this was not found to be significant ($p < 0.944$, $n = 3$) (Figure 7K).

DISCUSSION

In this study, novel shared disease mechanisms which contribute to human microphthalmia using patient-derived induced pluripotent stem cell (iPSC) OVs were described. A greater understanding of these mechanisms is critical for developing potential therapies and identifying molecular markers that may improve diagnostic rates. The overproduction of ECM components, including LUM, NID2, and COL4A1 that encapsulate and infiltrate the OV, may potentially physically limit ocular growth and development, resulting in eye malformations such as microphthalmia. Dysregulation of early ocular, apoptosis-, and cell proliferation-associated genes resulted in OVs with significantly reduced diameters, increased cell death and reduced cell proliferation contributing to the "small eye" phenotype. Furthermore, treatment with caspase-8 inhibitor Z-IETD-FMK significantly attenuated apoptosis in one patient (P1), suggesting targeting cell death pathways as a potential prophylactic therapy.

Pathogenic variants disrupt GRNs controlling early eye development

The reduced diameters of microphthalmia patient OVs in our study recapitulate the human phenotype. Further work is required to understand vesicle growth dynamics as vesicle diameter did not increase significantly between day 35 and 50. Previous studies of *VSX2*-associated microphthalmia also reported reduced diameters of patient OVs coupled with reduced cell proliferation (Phillips et al., 2014). Gamm et al. posited that changes to the expression of EFTFs such as VSX2 may influence cell proliferation and vesicle diameter as these changes were only observed following the onset of VSX2 expression at ~ day 30 (Phillips et al., 2014). Similarly, we only observed changes to cell proliferation in our patient vesicles from day 35, strengthening the hypothesis that the onset of these defects coincides with changes to VSX2 expression driving neural retina (NR) formation in the optic cup. Both our data and previous studies show dysregulation of early ocular GRNs due to reduced expression of early ocular genes such as *RAX*, *HES5*, *SIX6*, and *LHX5*. In contrast, *LHX5* was downregulated in our microphthalmia patients but upregulated in *VSX2*-associated microphthalmia (Phillips et al., 2014). Animal studies showed that loss of *LHX1* and *LHX5* impaired OV formation as cells acquired a pigment-like fate rather than NR (Inoue et al., 2013). VSX2 was also shown to bind to an intergenic region adjacent to *LHX5* to regulate its expression in early eye development but not to *LHX1* (Capowski et al., 2016). LHX1 and LHX5 may act as cell fate-determining transcription factors in a larger GRN that is critical in NR formation (Inoue et al., 2013). Our data showed significant loss of *LHX5* expression at both day 20 and 35 but of *LHX1* only at day 35. This is in accordance with previous data that showed LHX5 induces *LHX1* expression, which is required for OV patterning from the forebrain (Inoue et al., 2013). This regulatory relationship where *LHX1* becomes transcriptionally active downstream to LHX5 activity has also been observed in cortical development (Miquelajáuregui et al., 2010). Synthesizing our data and previous work, we can suggest a purported role for LHX5 in early development that when disrupted, may contribute to ocular malformation as clinically observed in our patients. Although the role of VSX2 in optic cup morphogenesis has been widely reported, this study may further support the essential role of LHX5 and LHX1 in human early eye development as VSX2 does not act independently to determine the NR fate of progenitors (Gamm et al., 2019). Alternatively, the pathways involved in early ocular development may be modulated in a mutation-dependent manner which culminates in a common downstream disease pathway. *MITF* expression is upregulated in both datasets, further strengthening the notion that the acquisition of a more RPE-like rather than NR cell fate is a common abnormality in microphthalmia patients and multiple genes may act in this pathway (Phillips et al., 2014).

Although not directly related to early ocular GRNs, mitochondrial disease caused by variants in *HCCS*, *COX7B*, and

Figure 4. Expression of ECM proteins in day 35 optic vesicles

(A) Western blot showing expression levels of COL4A1, NID2, and LUM in WT, P1, and P2 optic vesicles at day 35. β-Actin was used as a loading control for normalization of expression levels.

(B) Quantification of COL4A1, NID2, and LUM expression levels from the western blot.

(C–E) Expression patterns of COL4A1 in WT, P1, and P2 optic vesicles at day 35.

(F–H) Expression patterns of NID2 in WT, P1, and P2 optic vesicles at day 35.

(I–K) Expression patterns of LUM in WT, P1, and P2 optic vesicles at day 35. Experiments were performed with $n = 3$ from one clone per condition.

$p < 0.05$ (*), $p < 0.01$ (**), $p < 0.001$ (***). All results are expressed as mean ± SD.

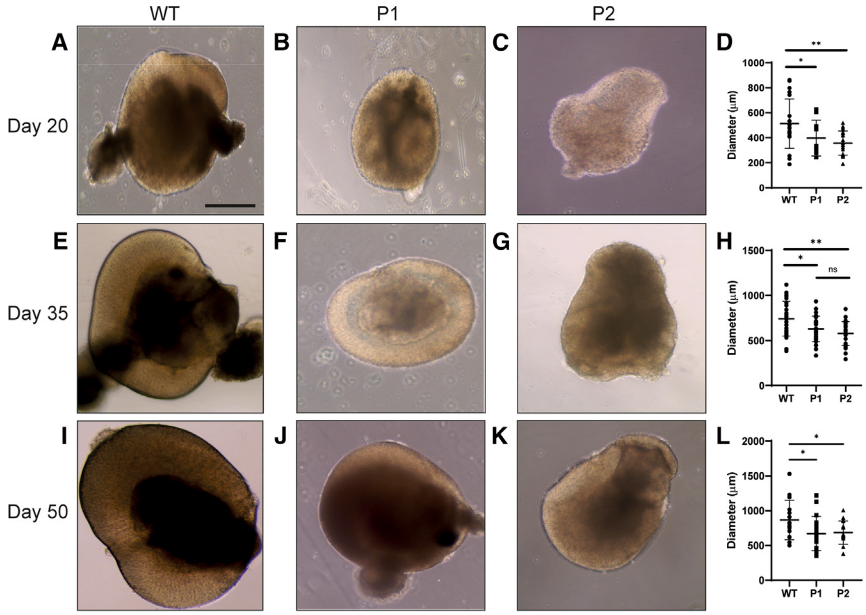

Figure 5. Diameters of WT, P1, and P2 optic vesicles
(A–C) Light microscopy images of WT, P1, and P2 optic vesicles at day 20.
(D) Diameters of WT, P1, and P2 optic vesicles at day 20.
(E–G) Light microscopy images of WT, P1, and P2 optic vesicles at day 35.
(H) Diameters of WT, P1, and P2 optic vesicles at day 35.
(I–K) Light microscopy images of WT, P1, and P2 optic vesicles at day 50.
(L) Diameters of WT, P1, and P2 optic vesicles at day 50. For all conditions, $n = 20$. $p < 0.05$ (*), $p < 0.01$ (**), $p < 0.001$ (***). All results are expressed as mean ± SD.

NDUFB11 has also been previously associated with MAC cases (Indrieri and Franco, 2021; Wimplinger et al., 2007; Eintracht et al., 2020b). Anomalous mitochondrial gene expression in microphthalmia OVs may indicate mitochondrial dysfunction contributes to ocular malformation. Previous studies have suggested mitochondrial dysfunction may underlie some developmental disorders due to mitochondrial involvement in early neuroectodermal differentiation (Zhu et al., 2016; Indrieri and Franco, 2021). This study introduces mitochondrial changes as a possible shared disease mechanism in MAC but further research is required to investigate mitochondrial dysfunction as an underlying shared etiology.

While the disruption of GRNs involved in early eye development has been well characterized, the downstream changes to cellular function that underlie disease pathophysiology are still largely unknown (Eintracht et al., 2020a). GO term enrichment analysis revealed global changes in cell processes such as signal transduction, cell adhesion, cell-cell communication, and differentiation, but further work is required to broaden our understanding of how genetic mutations disrupt these cellular functions in microphthalmia.

Upregulation of ECM components may overly constrict optic cup morphogenesis resulting in ocular malformation

Critical ECM components were upregulated in microphthalmia vesicles at day 20 and 35. The signaling and mechanical properties of the ECM that guide optic cup morphogenesis have been widely described in early eye development (Bryan et al., 2016, 2020; Eamegdool et al., 2020; Oltean et al., 2016; Casey et al., 2021). Research suggests *LUM* variants and polymorphisms are potentially associated with human myopia, playing a role in binding collagen fibrils and constricting their diameter to regulate scleral size (Lyu et al., 2021; Amjadi et al., 2013; Wang et al., 2017). As collagen fibrils have been shown to regulate scleral development, LUM action may control eye globe size during development (Coudrillier et al., 2015). In both *Lum*$^{-/-}$ zebrafish (antisense oligonucleotide morpholino gene knockdown) and stable transgenic mouse myopia models, the loss of ECM components increased axial length and resulted in myopia. The morpholinos were injected into fertilized eggs and a phenotype was first observed at 22 hours post fertilization (hpf) and still observed by 6 dpf (Yeh et al., 2010). By 6 dpf, the morpholino would not be effective in the zebrafish, suggesting that initial knockdown of LUM may have a more long-term impact, for example through autoregulation. Alternatively, the loss of LUM at early developmental stages may disrupt the spatiotemporal GRNs in the early eye, where the loss of a gene at its critical spatiotemporal expression window is irrecoverable and results in irreversible ocular malformation.

Involvement of the retinal basement membrane (RBM) in regulating eye size has been well documented in previous studies, diverting our attention to the composition of RBM ECM proteins (Halfter et al., 2006, 2015). Although only detected in human fetal tissue from 9 weeks post conception, it has been identified in 24 hpf zebrafish and E4 stage chicks, suggesting involvement in early eye

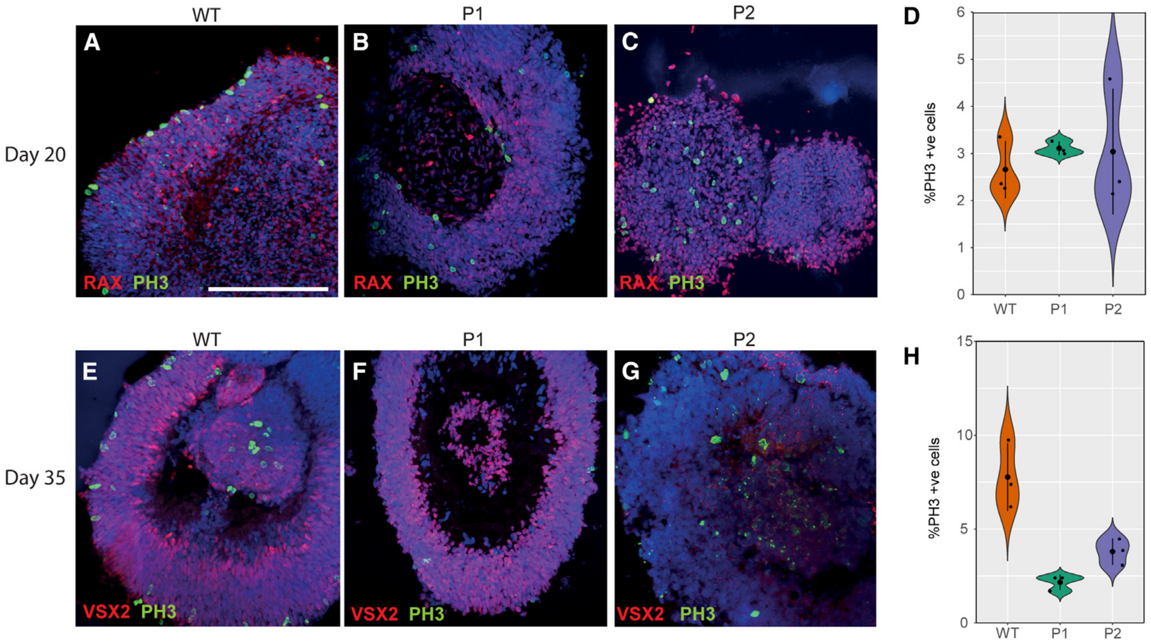

Figure 6. Reduced proliferation in microphthalmia optic vesicles
(A–C) pH3+ cells detected in WT, P1, and P2 optic vesicles at day 20.
(D) Quantification of pH3+ cells in WT, P1, and P2 optic vesicles at day 20.
(E–G) pH3+ cells detected in WT, P1, and P2 optic vesicles at day 35.
(H) Quantification of pH3+ cells in WT, P1, and P2 optic vesicles at day 35. Experiments were performed with $n = 3$ from two clones per condition.
$p < 0.05$ (*), $p < 0.01$ (**), $p < 0.001$ (***). All results are expressed as mean ± SD.

development (Balasubramani et al., 2010; Bryan et al., 2020). NID2 has a critical role in the formation of the RBM, a structure comprising layers of ECM that anchor the developing neuroepithelium of the NR to drive ocular development by the action of physical forces (Miosge et al., 2001; Bryan et al., 2020). As a major component of the RBM, NID2 was also found to have the most interactions with other BM ECM proteins, binding collagen type IV, and laminin to create subcomplexes that aggregate to form the BM critical for eye morphogenesis (Balasubramani et al., 2010). Previous studies showed that the loss of NID2 significantly impaired optic cup morphogenesis as it likely regulates cell movement and RPE flattening in the developing optic cup (Bryan et al., 2020). Loss of nidogen has also shown to cause coloboma in *foxd3;tfap2a* mutants by disrupting epithelial cell migration during morphogenesis (Yoon et al., 2020; Bryan et al., 2020). Furthermore, mutations in *NID1*, an important paralog of *NID2*, have also been directly associated with ocular malformations, suggesting an essential role for these proteins in eye development (Trejo-Reveles et al., 2023). Mass spectrometry also revealed novel interactions between nidogen and LUM in the brain ECM, where altered expression levels of both proteins disrupted brain ECM composition (Rodrigues-Amorim et al., 2022). It is possible that similar interactions exist in the developing eye, such that dysregulated ECM homeostasis results in disrupted ECM organization and function.

COL4A1 and COL4A4 are other components of the RBM that provide tensile strength and play a critical role in oculogenesis (Halfter et al., 2015; Eamegdool et al., 2020). Studies into fetal eye development reported upregulated collagen type IV expression and disrupted expression patterns in microphthalmia eyes, in accordance with our data (Sijilmassi et al., 2021). While associated with human MAC phenotypes, there is a lack of data describing the role of collagen in eye morphogenesis and its interactions (Deml et al., 2014; Matías-Pérez et al., 2018). This study aims to shed further light on the role of the ECM with an emphasis on collagen, LUM, and nidogen in particular. It is suggested that a denser ECM contributes to coloboma as its failed displacement obstructs optic fissure fusion (Chan et al., 2020). This cannot be tested in OVs as they do not contain optic fissure; hence, further work is required in alternative disease models, such as the chick or zebrafish, to characterize the interaction and cooperation of ECM organization and function in the developing eye. Indeed, *nid1* expression was detected in both the chick and zebrafish optic fissure margin (OFM) (Trejo-Reveles

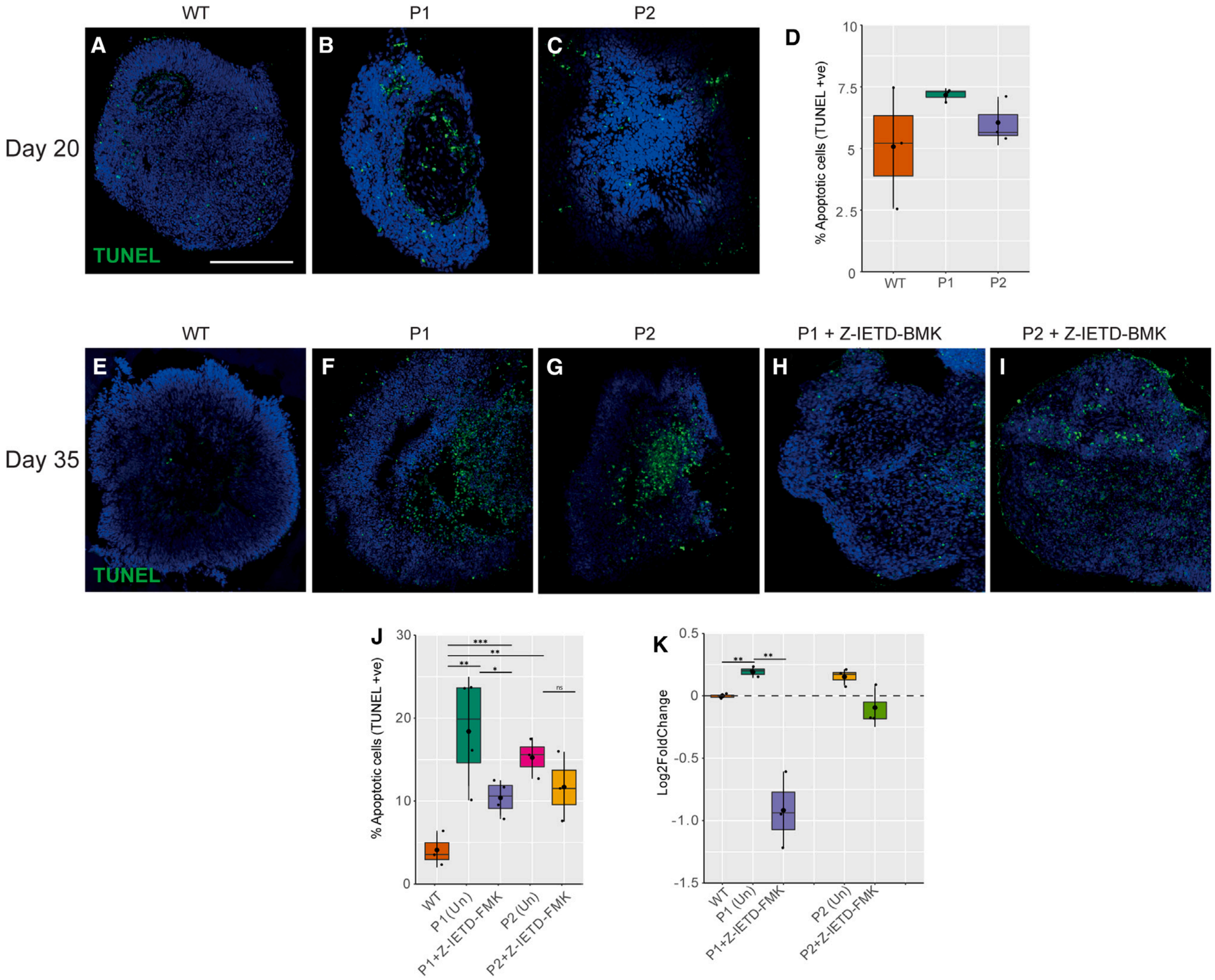

Figure 7. Apoptosis in microphthalmia optic vesicles

(A–C) TUNEL+ cells detected in WT, P1, and P2 optic vesicles at day 20.
(D) Quantification of apoptosis through TUNEL+ cells in WT, P1, and P2 optic vesicles at day 20.
(E–G) TUNEL+ cells detected in WT, P1, and P2 optic vesicles at day 35.
(H and I) TUNEL+ cells detected in Z-IETD-FMK-treated P1 and P2 optic vesicles at day 35.
(J) Quantification of apoptosis through TUNEL+ cells in WT and treated and untreated P1 and P2 optic vesicles at day 35.
(K) Quantification of caspase-8 activity in WT and treated and untreated P1 and P2 optic vesicles by firefly caspase-8 assay. Experiments were performed with $n = 3$ from two clones per condition. $p < 0.05$ (*), $p < 0.01$ (**), $p < 0.001$ (***). All results are expressed as mean ± SD.

et al., 2023; Knickmeyer et al., 2018). The conserved expression in the OFM across species and the coloboma phenotype observed in *nid1* morphant zebrafish suggest an essential role for nidogen in optic fissure closure and further investigation of human orthologs *NID1* and *NID2* as novel candidate coloboma genes (Knickmeyer et al., 2018; Trejo-Reveles et al., 2023).

The upregulation of LUM and other ECM proteins such as NID2 and COL4A1 in microphthalmia patient OVs in contrast to the loss of Lumican in animal models of increased axial length may be indicative of an inversely proportional relationship between the ECM and axial length (Eiraku et al., 2012). Oltean et al. (2016) demonstrated that the ECM is critical to optic cup invagination as it provides mechanical forces that constrain the OV, initiating the invagination process (Oltean et al., 2016). *LUM* has been associated with conferring resistance and structure to the ECM through collagen organization, suggesting overexpression may generate excessive tensile force on the developing eye (Rodrigues-Amorim et al., 2022).

Our data highlight the upregulation of multiple key ECM components; the overexpression of RBM proteins such as NID2 and COL4A1 may create large subcomplexes that aggregate to create an abnormally large BM in microphthalmic patients. This hypothesis is supported from zebrafish models that showed mechanical cues provided by the ECM are critical for organogenesis, suggesting molecular signals and genetic gradients alone are not sufficient to drive development (Monnot et al., 2022).

This study is the first to map the expression patterns of NID2, LUM, and COL4A1 in stem cell-derived OVs and show their association in microphthalmia. The *in vitro* localization of ECM components identified in this study largely mirror their *in vivo* localization, such as NID2 expression was localized to the basal aspect of the OV, similar to basal deposits of nidogen detected in the developing zebrafish neuroretina and RPE or the more ubiquitous expression of COL4A1 in the mouse eye similar to the patterns we observed *in vitro* (Bryan et al., 2020; Sijilmassi et al., 2021). However, due to the complex microenvironment of the developing OV *in vivo* and its interactions with surrounding tissues, the expression patterns of these ECM proteins *in vitro* will not identically mimic those *in vivo*. Further work is required to assess the role of these ECM proteins in both expanded patient cohorts and *in vivo* models of microphthalmia.

Cell proliferation and apoptosis may contribute to the microphthalmia phenotype

Genomic instability through pathogenic variants can contribute to changes in the cell cycle, inhibiting proliferation and increasing apoptosis (Matos-Rodrigues and Martins, 2021; Matos-Rodrigues et al., 2020; French et al., 2013). Overexpression of *Cdkn2b* in *Xenopus* microphthalmia models correlated with reduced cell proliferation due to cell cycle misregulation (Aldiri et al., 2013), and upregulation of *CDKN2A* and *CDKN2B* reduces proliferation in the developing retina (Dimaras et al., 2008). These genes are negative cell cycle regulators that transition cells from the G1 to S phase and are effectors of the TGF-β pathway-mediated cell cycle inhibition that reduces proliferation (Hannon and Beach, 1994; Chaum et al., 2015; Teotia et al., 2017; Xia et al., 2021; Soto et al., 2005). At day 20 and 35, *CDKN2A* and *CDKN2B* were upregulated in both microphthalmia patients, as were TGF-β-related genes at day 20. Although cell proliferation levels did not significantly differ between WT and patient vesicles at day 20, transcriptional irregularities may precede such changes, as *CDKN2A* and *CDKN2B* both target CDK genes that regulate transcription factors active in the G1/S phase transition (Xia et al., 2021). These targets induce positive and negative feedback loops that are temporally spaced to facilitate correct progression of the cell cycle, suggesting a possible delay in effect (Eser et al., 2011; Georgi et al., 2002). Interestingly, reduced expression of *Cdkn2a* in TASP1-deficient mice significantly decreased cranial and ocular malformations, including microphthalmia, suggesting inhibition of these genes may be required for correct retinal progenitor proliferation to drive ocular morphogenesis (Takeda et al., 2015). Our data showed *SIX6* was downregulated alongside other critical ocular genes such as *RAX*, *HES1*, and *PAX6* that temporally coincided with *CDKN2A* and *CDKN2B* upregulation, suggesting these genes may be regulated by similar GRNs that control early ocular gene expression (Gamm et al., 2019; Phillips et al., 2014; Matos-Rodrigues and Martins, 2021).

Apart from regulating cell proliferation, *CDKN2A* and *CDKN2B* also promote p53-dependent apoptosis and cell-cycle arrest in the retina and RPE previously associated with animal models of microphthalmia (Takeda et al., 2015; Chaum et al., 2015; Xia et al., 2021; Hannon and Beach, 1994; Rodrigues et al., 2013). In accordance, our RNA-seq data showed enrichment of the p53 pathway in the microphthalmia patient vesicles. Apoptosis has been described in animal models of microphthalmia, but not in human *in vitro* systems (Wu et al., 2003; Hettmann et al., 2000; Dash et al., 2020; Cheng et al., 2018; Capowski et al., 2016; Phillips et al., 2014). We identified global upregulation of upstream regulators of apoptosis, such as *XAF1*, *IRF1*, and *FZD9*, and caspase-recruiters inducing a caspase cascade, including CASP8, which drives apoptosis (Jeong et al., 2018). FZD9 is a receptor for Wnt ligands in Wnt signaling and its haploinsufficiency has been previously associated with increased apoptosis in neural progenitor cells (Chailangkarn and Muotri, 2017; Chailangkarn et al., 2016). Our data show reduced expression of *FZD9* and *FZD10* and further Wnt pathway misregulation, suggesting Wnt abnormalities may contribute to the increased apoptosis observed in the retinal progenitor cells. Abnormal Wnt signaling has also been identified in other cell death pathways such as necrosis or ferroptosis and the TNF death receptor pathway (Li et al., 2019; Wang et al., 2022; Hiyama et al., 2013), where dysfunctional signaling observed in patient OVs may promote cell death through multiple pathways. Wnt signaling has been shown to be critical to early eye development and is involved in the regulation of cellular functions such as apoptosis, proliferation, differentiation, and tissue specification during morphogenesis (Fuhrmann, 2008; Cheng et al., 2018; Li et al., 2016).

This study is the first to describe upregulated caspase cascades associated with microphthalmia in humans although a purported role has been previously hypothesized (Wimplinger et al., 2007). Caspase effectors of apoptosis were not upregulated in microphthalmia patients in our study. It is possible that apoptosis is effected

through other signaling pathways activated by CASP8 such as the TNF death receptor pathway as reflected in our transcriptomics data (Tummers and Green, 2017). Further evidence indicates this is a common pathway in microphthalmia from the *VSX2*-associated microphthalmia dataset. Although no caspases were upregulated in their dataset, Phillips et al. reported upregulation at day 30 of *TNFRSF19*, *TNFRSF21*, and *TNFRSF10D*, all effectors of the activation of the CASP8-TNF death receptor apoptosis pathway (Phillips et al., 2014; Sakamaki and Satou, 2009). Some discrepancies between datasets may exist as our data measure gene expression levels at day 35 while Phillips et al. measured them at day 30. Our data combined with previous studies of microphthalmia may suggest apoptosis modulated through CASP8 and the TNF death receptor is a shared pathway contributing to ocular malformation, as alternative cell death pathways are likely involved in developmental eye disorders (Gregory-Evans et al., 2011).

Therapeutic interventions reduce apoptosis in microphthalmia patient OVs

This study highlighted the therapeutic potential of inhibiting aberrant cell death. The caspase inhibitor Z-IETD-FMK showed significant reduction in apoptosis in P1; however, future work should involve a larger sample size. Pharmacological inhibition of apoptosis pathways has previously been shown to be effective in zebrafish models *gup* and *pax2.1* models of coloboma (Gregory-Evans et al., 2011). However, the effects on phenotype between these models differed greatly, as the coloboma phenotype was only corrected in the *gup* model (Gregory-Evans et al., 2011). Expanding on this work, Lusk and Kwan (2022) found that *pax2a*, but not *pax2b*, influences cell survival in the optic fissure and *pax2a* mutants displayed increased apoptosis and coloboma (Lusk and Kwan, 2022). Inhibition of apoptosis with anti-apoptotic factor *Bcl-xl* RNA did partially rescue the coloboma phenotype in these mutant zebrafish (Lusk and Kwan, 2022). These studies show apoptosis has a clear role in developmental eye disorders, particularly the MAC phenotype, which can also be targeted for rescue. In this study, apoptosis was only significantly reduced in P1 with a trend reduction in P2. The differing response to the drug may have been due to uptake in OVs as P1 vesicles displayed enhanced structural integrity, lamination, and self-organization of VSX2+ cells compared to P2. A previous study identified an enhanced photoreceptor-specific response to drugs modulating apoptosis, and this effect may be mirrored in retinal progenitor cells (Dorgau et al., 2022). It is also possible that the more severe microphthalmia phenotype (and underlying genetic basis) of P2 requires a higher dose of Z-IETD-FMK to significantly influence apoptosis pathways.

However, it is unlikely to be an effective treatment for patients due to the need for *in utero* administration at a very early stage and the challenge of detecting MAC phenotypes at this critical time point (Smets et al., 2006; Lindsay et al., 2016; Mysore et al., 2019). Curcumin, a natural polyphenol with antiapoptotic activity, has been suggested as a prophylactic therapy for mothers at increased risk i.e., with a positive family history, but further work is required to assess its safety and efficacy in humans. For those who have a genetic diagnosis, family planning advice should be sought as options including preimplantation genetic diagnosis may be applicable (Treff et al., 2020). For microphthalmia, depending on phenotypic severity, a post-natal therapeutic approach which permits continued growth and maturation of the eye with preservation or improvement in residual vision may be more amenable.

Limitations of the study

As ECM genes are upregulated *in vitro* in the absence of the microenvironment of the developing eye and the extrinsic cues that induce structural changes in the OV such as those arising from the surface ectoderm, there is possible overlap between ECM regulation and the GRNs controlling early eye development. This also may explain why ECM overproduction is common between microphthalmia patients with differing genotypes. This hypothesis is supported from previous studies into *VSX2*-associated microphthalmia. In both our patients and *VSX2*-associated microphthalmia, we observed common upregulation of ECM components such as collagen and laminin family proteins, fibronectin, and hyaluronan and proteoglycan-link proteins that play significant roles in eye development (Phillips et al., 2014; Casey et al., 2021; Turksen et al., 1985). The temporal discrepancies in upregulation of ECM genes between microphthalmia patients may suggest mutation-specific downstream effects within a common molecular architecture. Our data support this hypothesis as LUM expression was significantly upregulated at day 20 in P1 but at day 35 in P2. Further work is required to investigate ECM production in microphthalmia patients with variants affecting different cellular processes or that represent nodes in different GRNs to fully understand the common nature of ECM overproduction in microphthalmia. However, it is also possible that the upregulation of MITF observed in microphthalmia patient OVs may promote ECM production due to an imbalance between the presumptive NR and RPE (Eamegdool et al., 2020). Therefore, changes to ECM production may be a secondary event of ocular malformation, not a primary cause driving aberrant eye morphogenesis. This study is limited as it has not investigated the spatial and temporal changes to the ECM *in vivo* to provide further evidence to either hypothesis.

Retinal differentiation of human iPSCs (hiPSCs) is highly variable in efficiency between individual cell lines and differentiation protocols (Capowski et al., 2019; Cowan et al., 2020). Sample sizes are often small due to the intense financial and labor costs in generating retinal organoids as well as inherent inter- and intra-sample variability (Afanasyeva et al., 2021). While some markers may predict retinal differentiation efficiency such as *Meis1* (Wang et al., 2018), a complete picture of the differentiation capacity of each clone of a hiPSC line is only possible following intensive clonal screenings that are time, labor, and cost intensive (Cowan et al., 2020). It is possible that not all variability between hiPSC lines, clones, and differentiation rounds can be controlled for and may influence the data.

The genetic background of each generated cell line will influence its differentiation capacity, whether through heterogeneous gene expression patterns or epigenetic "memory" regulating gene expression (Kim et al., 2010; Lewis and Kats, 2021). Indeed, modeling of human disease using patient-specific organoids revealed large transcriptional heterogeneity between patients resulting in diverse transcriptomic profiles that may alter cell fate decisions *in vitro* (Krieger et al., 2021; Chamling et al., 2021). The creation of isogenic lines eliminates these concerns as all hiPSC lines are generated on the same background, enabling any abnormalities to be specifically associated with the pathogenic variant. The strength of the conclusions made by this study is limited by the lack of isogenic lines. Furthermore, this study would be improved by investigating these shared mechanisms in a larger cohort of genotyped microphthalmia patients.

EXPERIMENTAL PROCEDURES

Resource availability

Lead contact

All relevant data supporting the key findings of this study are available within the article and its supplemental information files. Further information and requests can be made to the corresponding author, Mariya Moosajee (m.moosajee@ucl.ac.uk).

Materials availability

There were no new reagents generated for this study.

Data and code availability

All RNA sequencing data generated in this study have been deposited in the NCBI's Gene Expression Omnibus database (GEO Series Accession Number: GSE265940).

Methods

Ethics and consent

This study complied with all tenets of the Declaration of Helsinki, This study falls under ethics 11/LO/243 NRES study of congenital eye disease under the National Research Ethics Service from Moorfields Eye Hospital NHS Foundation Trust. Written informed consent for publication of the participants' details and/or their images was obtained from the participants.

hiPSC derivation

iPSCs were derived from human dermal fibroblasts obtained from WTs and patients, with informed consent, and characterized for pluripotency markers and absence of chromosomal anomalies, as described in detail in Méjécase et al. (2020), Harding et al. (2021), Méjécase et al. (2020), and Harding et al. (2021).

OV differentiation

Embryoid bodies (EBs) were formed as previously described (Eintracht et al., 2022), and differentiation was performed as outlined (Mellough et al., 2015; Chichagova et al., 2019). 48 h after EB formation (day 2), EBs were plated by gentle pipetting into 60 mm TC-treated culture dishes. Cells were cultured in neural induction media, (DMEM/F12, knockout serum residue [KOSR], 2% B27, 1x non-essential amino acids (NEAA), 1% P/S, 1xGlutaMAX, and 5 ng/mL IGF-1) with decreasing KOSR concentrations: 20% from day 2–7, 15% from day 7–11, and 10% from day 11–18. From day 18, cells were cultured in retinal differentiation media (DMEM/F12, 10% FBS, 2% B27, 1xNEAA, 1xGlutaMAX, 1% P/S, 5 ng/mL IGF-1, 0.1 mM taurine, 40 ng/mL triiodothyronine, and 0.5 μM retinoic acid).

Statistics

Statistical analysis was performed using Excel and Prism (Microsoft, USA; GraphPad, USA). A two-tailed unpaired Student's t test was used for comparison studies for diameter, pH3, and TUNEL quantitation; however, a two-tailed paired Student's t test was used for comparison studies between conditions for western blotting as individual rounds of differentiation are compared. A p value of <0.05 was considered statistically significant. Significance levels were set when $p < 0.05$ (*), $p < 0.01$ (**), $p < 0.001$ (***). All results are expressed as mean ± SD unless specified. All experiments were performed with $n = 3$ replicates grown at separate times in separate dishes.

A sample size of $n = 3$ was chosen as each sample is time, labor, and cost intensive to generate and cannot be equated to cell lines used for high-throughput experiments which are simpler and cheaper to maintain. This is commonly seen in the literature as shown in other studies investigating ocular development and disease, with some only using up to three clonal hiPSC lines per condition (Parfitt et al., 2016).

SUPPLEMENTAL INFORMATION

Supplemental information can be found online at https://doi.org/10.1016/j.stemcr.2024.05.001.

ACKNOWLEDGMENTS

The authors would like to thank the patients and their families for their involvement in this study. The authors would also like to acknowledge their funding sources: Wellcome Trust 205174/Z/16/Z, National Centre for the Replacement, Refinement and Reduction of Animals in Research, NIHR Moorfields Biomedical Research Centre and Moorfields Eye Charity.

AUTHOR CONTRIBUTIONS

Conceptualization, M.M., N.O., and J.E.; methodology, N.O., J.E., and P.H.; software, N.O.; validation, N.O., J.E., and P.H.; formal

analysis, N.O. and J.E.; investigation, N.O., J.E., and P.H.; resources, M.M.; data curation, N.O. and J.E.; visualization, N.O. and J.E.; writing – original draft, J.E.; writing – review and editing, M.M., N.O., J.E., and P.H.; supervision, N.O. and M.M.; project administration, M.M., N.O., and J.E.; funding acquisition, M.M.

DECLARATION OF INTERESTS

The authors declare no competing interests.

Received: September 13, 2023
Revised: April 30, 2024
Accepted: May 2, 2024
Published: May 30, 2024

REFERENCES

Afanasyeva, T.A.V., Corral-Serrano, J.C., Garanto, A., Roepman, R., Cheetham, M.E., and Collin, R.W.J. (2021). A look into retinal organoids: methods, analytical techniques, and applications. Cell. Mol. Life Sci. *78*, 6505–6532.

Aldiri, I., Moore, K.B., Hutcheson, D.A., Zhang, J., and Vetter, M.L. (2013). Polycomb repressive complex prc2 regulates xenopus retina development downstream of wnt/β-catenin signaling. Development *140*, 2867–2878.

Amjadi, S., Mai, K., Mccluskey, P., and Wakefield, D. (2013). The role of lumican in ocular disease. Int. Sch. Res. Notices *2013*, 632302.

Asaoka, Y., Hata, S., Namae, M., Furutani-Seiki, M., and Nishina, H. (2014). The hippo pathway controls a switch between retinal progenitor cell proliferation and photoreceptor cell differentiation in zebrafish. PLoS One *9*, e97365.

Bai, X., Dilworth, D.J., Weng, Y.-C., and Gould, D.B. (2009). Developmental distribution of collagen iv isoforms and relevance to ocular diseases. Matrix Biol. *28*, 194–201.

Balasubramani, M., Schreiber, E.M., Candiello, J., Balasubramani, G.K., Kurtz, J., and Halfter, W. (2010). Molecular interactions in the retinal basement membrane system: a proteomic approach. Matrix Biol. *29*, 471–483.

Basharat, R., Rodenburg, K., Rodríguez-Hidalgo, M., Jarral, A., Ullah, E., Corominas, J., Gilissen, C., Zehra, S.T., Hameed, U., Ansar, M., and De Bruijn, S.E. (2023). Combined single gene testing and genome sequencing as an effective diagnostic approach for anophthalmia and microphthalmia patients. Genes *14*, 1573.

Bryan, C.D., Casey, M.A., Pfeiffer, R.L., Jones, B.W., and Kwan, K.M. (2020). Optic cup morphogenesis requires neural crest-mediated basement membrane assembly. Development *147*, dev181420.

Bryan, C.D., Chien, C.-B., and Kwan, K.M. (2016). Loss of laminin alpha 1 results in multiple structural defects and divergent effects on adhesion during vertebrate optic cup morphogenesis. Dev. Biol. *416*, 324–337.

Capowski, E.E., Samimi, K., Mayerl, S.J., Phillips, M.J., Pinilla, I., Howden, S.E., Saha, J., Jansen, A.D., Edwards, K.L., Jager, L.D., et al. (2019). Reproducibility and staging of 3d human retinal organoids across multiple pluripotent stem cell lines. Development *146*, dev171686.

Capowski, E.E., Wright, L.S., Liang, K., Phillips, M.J., Wallace, K., Petelinsek, A., Hagstrom, A., Pinilla, I., Borys, K., Lien, J., et al. (2016). Regulation of wnt signaling by vsx2 during optic vesicle patterning in human induced pluripotent stem cells. Stem Cell. *34*, 2625–2634.

Carrara, N., Weaver, M., Piedade, W.P., Vöcking, O., and Famulski, J.K. (2019). Temporal characterization of optic fissure basement membrane composition suggests nidogen may be an initial target of remodeling. Dev. Biol. *452*, 43–54.

Casey, M.A., Lusk, S., and Kwan, K.M. (2021). Build me up optic cup: intrinsic and extrinsic mechanisms of vertebrate eye morphogenesis. Dev. Biol. *476*, 128–136.

Chailangkarn, T., and Muotri, A.R. (2017). Modeling williams syndrome with induced pluripotent stem cells. Neurogenesis *4*, e1283187.

Chailangkarn, T., Trujillo, C.A., Freitas, B.C., Hrvoj-Mihic, B., Herai, R.H., Yu, D.X., Brown, T.T., Marchetto, M.C., Bardy, C., Mchenry, L., et al. (2016). A human neurodevelopmental model for williams syndrome. Nature *536*, 338–343.

Chamling, X., Kallman, A., Fang, W., Berlinicke, C.A., Mertz, J.L., Devkota, P., Pantoja, I.E.M., Smith, M.D., Ji, Z., Chang, C., et al. (2021). Single-cell transcriptomic reveals molecular diversity and developmental heterogeneity of human stem cell-derived oligodendrocyte lineage cells. Nat. Commun. *12*, 652.

Chan, B.H.C., Moosajee, M., and Rainger, J. (2020). Closing the gap: mechanisms of epithelial fusion during optic fissure closure. Front. Cell Dev. Biol. *8*, 620774.

Chaum, E., Winborn, C.S., and Bhattacharya, S. (2015). Genomic regulation of senescence and innate immunity signaling in the retinal pigment epithelium. Mamm. Genome *26*, 210–221.

Cheng, X.-N., Shao, M., and Shi, D.-L. (2018). Mutation of frizzled8a delays neural retinal cell differentiation and results in microphthalmia in zebrafish. Int. J. Dev. Biol. *62*, 285–291.

Chichagova, V., Dorgau, B., Felemban, M., Georgiou, M., Armstrong, L., and Lako, M. (2019). Differentiation of retinal organoids from human pluripotent stem cells. Curr. Protoc. Stem Cell Biol. *50*, e95.

Coudrillier, B., Pijanka, J., Jefferys, J., Sorensen, T., Quigley, H.A., Boote, C., and Nguyen, T.D. (2015). Collagen structure and mechanical properties of the human sclera: analysis for the effects of age. J. Biomech. Eng. *137*, 041006.

Cowan, C.S., Renner, M., De Gennaro, M., Gross-Scherf, B., Goldblum, D., Hou, Y., Munz, M., Rodrigues, T.M., Krol, J., Szikra, T., et al. (2020). Cell types of the human retina and its organoids at single-cell resolution. Cell *182*, 1623–1640.e34.

Dash, S., Brastrom, L.K., Patel, S.D., Scott, C.A., Slusarski, D.C., and Lachke, S.A. (2020). The master transcription factor sox2, mutated in anophthalmia/microphthalmia, is post-transcriptionally regulated by the conserved rna-binding protein rbm24 in vertebrate eye development. Hum. Mol. Genet. *29*, 591–604.

Deml, B., Reis, L.M., Maheshwari, M., Griffis, C., Bick, D., and Semina, E.V. (2014). Whole exome analysis identifies dominant col4a1 mutations in patients with complex ocular phenotypes involving microphthalmia. Clin. Genet. *86*, 475–481.

Den Hollander, A.I., Biyanwila, J., Kovach, P., Bardakjian, T., Traboulsi, E.I., Ragge, N.K., Schneider, A., and Malicki, J. (2010). Genetic defects of gdf6 in the zebrafish out of sight mutant and in human eye developmental anomalies. BMC Genet. *11*, 102.

Dimaras, H., Khetan, V., Halliday, W., Orlic, M., Prigoda, N.L., Piovesan, B., Marrano, P., Corson, T.W., Eagle, R.C., Jr., Squire, J.A., and Gallie, B.L. (2008). Loss of rb1 induces non-proliferative retinoma: increasing genomic instability correlates with progression to retinoblastoma. Hum. Mol. Genet. *17*, 1363–1372.

Dorgau, B., Georgiou, M., Chaudhary, A., Moya-Molina, M., Collin, J., Queen, R., Hilgen, G., Davey, T., Hewitt, P., Schmitt, M., et al. (2022). Human retinal organoids provide a suitable tool for toxicological investigations: a comprehensive validation using drugs and compounds affecting the retina. Stem Cells Transl. Med. *11*, 159–177.

Eamegdool, S.S., Sitiwin, E.I., Cioanca, A.V., and Madigan, M.C. (2020). Extracellular matrix and oxidative stress regulate human retinal pigment epithelium growth. Free Radic. Biol. Med. *146*, 357–371.

Eintracht, J., Toms, M., and Moosajee, M. (2020a). The use of induced pluripotent stem cells as a model for developmental eye disorders. Front. Cell. Neurosci. *14*, 265.

Eintracht, J., Corton, M., Fitzpatrick, D., and Moosajee, M. (2020b). Cugc for syndromic microphthalmia including next-generation sequencing-based approaches. Eur. J. Hum. Genet. *28*, 679–690.

Eintracht, J., Harding, P., Lima Cunha, D., and Moosajee, M. (2022). Efficient embryoid-based method to improve generation of optic vesicles from human induced pluripotent stem cells. F1000res. *11*, 324.

Eiraku, M., Adachi, T., and Sasai, Y. (2012). Relaxation-expansion model for self-driven retinal morphogenesis: a hypothesis from the perspective of biosystems dynamics at the multi-cellular level. Bioessays *34*, 17–25.

Eser, U., Falleur-Fettig, M., Johnson, A., and Skotheim, J.M. (2011). Commitment to a cellular transition precedes genome-wide transcriptional change. Mol. Cell *43*, 515–527.

French, C.R., Stach, T.R., March, L.D., Lehmann, O.J., and Waskiewicz, A.J. (2013). Apoptotic and proliferative defects characterize ocular development in a microphthalmic bmp model. Invest. Ophthalmol. Vis. Sci. *54*, 4636–4647.

Fuhrmann, S. (2008). Wnt signaling in eye organogenesis. Organogenesis *4*, 60–67.

Gamm, D.M., Clark, E., Capowski, E.E., and Singh, R. (2019). The role of fgf9 in the production of neural retina and rpe in a pluripotent stem cell model of early human retinal development. Am. J. Ophthalmol. *206*, 113–131.

Georgi, A.B., Stukenberg, P.T., and Kirschner, M.W. (2002). Timing of events in mitosis. Curr. Biol. *12*, 105–114.

Green, E.S., Stubbs, J.L., and Levine, E.M. (2003). Genetic rescue of cell number in a mouse model of microphthalmia:interactions between chx10 and g1-phase cell cycle regulators. Development *130*, 539–552.

Gregory-Evans, C.Y., Moosajee, M., Shan, X., and Gregory-Evans, K. (2011). Gene-specific differential response to anti-apoptotic therapies in zebrafish models of ocular coloboma. Mol. Vis. *17*, 1473–1484.

Guo, Y., Wang, P., Ma, J.H., Cui, Z., Yu, Q., Liu, S., Xue, Y., Zhu, D., Cao, J., Li, Z., et al. (2019). Modeling retinitis pigmentosa: retinal organoids generated from the ipscs of a patient with the ush2a mutation show early developmental abnormalities. Front. Cell. Neurosci. *13*, 361.

Halfter, W., Oertle, P., Monnier, C.A., Camenzind, L., Reyes-Lua, M., Hu, H., Candiello, J., Labilloy, A., Balasubramani, M., Henrich, P.B., and Plodinec, M. (2015). New concepts in basement membrane biology. FEBS J. *282*, 4466–4479.

Halfter, W., Winzen, U., Bishop, P.N., and Eller, A. (2006). Regulation of eye size by the retinal basement membrane and vitreous body. Invest. Ophthalmol. Vis. Sci. *47*, 3586–3594.

Hannon, G.J., and Beach, D. (1994). Pl5ink4b is a potentia| effector of tgf-β-induced cell cycle arrest. Nature *371*, 257–261.

Harding, P., Gore, S., Malka, S., Rajkumar, J., Oluonye, N., and Moosajee, M. (2022). Real-world clinical and molecular management of 50 prospective patients with microphthalmia, anophthalmia and/or ocular coloboma. Br. J. Ophthalmol. *107*, 1925–1935.

Harding, P., Lima Cunha, D., Méjécase, C., Eintracht, J., Toualbi, L., Sarkar, H., and Moosajee, M. (2021). Generation of human ipsc line (ucli013-a) from a patient with microphthalmia and aniridia, carrying a heterozygous missense mutation c.372c>a p.(asn124lys) in pax6. Stem Cell Res. *51*, 102184.

Hettmann, T., Barton, K., and Leiden, J.M. (2000). Microphthalmia due to p53-mediated apoptosis of anterior lens epithelial cells in mice lacking the creb-2 transcription factor. Dev. Biol. *222*, 110–123.

Hiyama, A., Yokoyama, K., Nukaga, T., Sakai, D., and Mochida, J. (2013). A complex interaction between wnt signaling and tnf-α in nucleus pulposus cells. Arthritis Res. Ther. *15*, r189.

Indrieri, A., and Franco, B. (2021). Linear skin defects with multiple congenital anomalies (lsdmca): an unconventional mitochondrial disorder. Genes *12*, 263.

Inoue, J., Ueda, Y., Bando, T., Mito, T., Noji, S., and Ohuchi, H. (2013). The expression of lim-homeobox genes, lhx1 and lhx5, in the forebrain is essential for neural retina differentiation. Dev. Growth Differ. *55*, 668–675.

Jeong, S.-I., Kim, J.-W., Ko, K.-P., Ryu, B.-K., Lee, M.-G., Kim, H.-J., and Chi, S.-G. (2018). Xaf1 forms a positive feedback loop with irf-1 to drive apoptotic stress response and suppress tumorigenesis. Cell Death Dis. *9*, 806.

Kim, J.Y., Park, R., Lee, J.H.J., Shin, J., Nickas, J., Kim, S., and Cho, S.-H. (2016). Yap is essential for retinal progenitor cell cycle progression and rpe cell fate acquisition in the developing mouse eye. Dev. Biol. *419*, 336–347.

Kim, K., Doi, A., Wen, B., Ng, K., Zhao, R., Cahan, P., Kim, J., Aryee, M.J., Ji, H., Ehrlich, L.I.R., et al. (2010). Epigenetic memory in induced pluripotent stem cells. Nature *467*, 285–290.

Knickmeyer, M.D., Mateo, J.L., Eckert, P., Roussa, E., Rahhal, B., Zuniga, A., Krieglstein, K., Wittbrodt, J., and Heermann, S. (2018). Tgfβ-facilitated optic fissure fusion and the role of bone morphogenetic protein antagonism. Open Biol. *8*, 170134.

Krieger, T.G., Le Blanc, S., Jabs, J., Ten, F.W., Ishaque, N., Jechow, K., Debnath, O., Leonhardt, C.-S., Giri, A., Eils, R., et al. (2021). Single-cell analysis of patient-derived pdac organoids reveals cell state heterogeneity and a conserved developmental hierarchy. Nat. Commun. *12*, 5826.

Lee, J.-H., Park, H.S., and Holmes, D.P. (2021). Elastic instabilities govern the morphogenesis of the optic cup. Phys. Rev. Lett. *127*, 138102.

Lewis, A.C., and Kats, L.M. (2021). Non-genetic heterogeneity, altered cell fate and differentiation therapy. EMBO Mol. Med. *13*, e12670.

Li, B., Lee, C., Cadete, M., Zhu, H., Koike, Y., Hock, A., Wu, R.Y., Botts, S.R., Minich, A., Alganabi, M., et al. (2019). Impaired wnt/β-catenin pathway leads to dysfunction of intestinal regeneration during necrotizing enterocolitis. Cell Death Dis. *10*, 743.

Li, J., Wang, S., Anderson, C., Zhao, F., Qin, Y., Wu, D., Wu, X., Liu, J., He, X., Zhao, J., and Zhang, J. (2016). Requirement of smad4 from ocular surface ectoderm for retinal development. PLoS One *11*, e0159639.

Lindsay, S.J., Xu, Y., Lisgo, S.N., Harkin, L.F., Copp, A.J., Gerrelli, D., Clowry, G.J., Talbot, A., Keogh, M.J., Coxhead, J., et al. (2016). Hdbr expression: a unique resource for global and individual gene expression studies during early human brain development. Front. Neuroanat. *10*, 86.

Lusk, S., and Kwan, K.M. (2022). Pax2a, but not pax2b, influences cell survival and periocular mesenchyme localization to facilitate zebrafish optic fissure closure. Dev. Dyn. *251*, 625–644.

Lyu, X.T., Song, Y.Z., and Zhang, F.J. (2021). Regulation of bfgf and tgf-β2 in human scleral fibroblasts by the lumican gene mutation associated with myopia. Zhonghua. Yan Ke Za Zhi. *57*, 277–283.

Matías-Pérez, D., García-Montaño, L.A., Cruz-Aguilar, M., García-Montalvo, I.A., Nava-Valdéz, J., Barragán-Arevalo, T., Villanueva-Mendoza, C., Villarroel, C.E., Guadarrama-Vallejo, C., La Cruz, R.V.-D., et al. (2018). Identification of novel pathogenic variants and novel gene-phenotype correlations in mexican subjects with microphthalmia and/or anophthalmia by next-generation sequencing. J. Hum. Genet. *63*, 1169–1180.

Matos-Rodrigues, G.E., Grigaravicius, P., Lopez, B.S., Hofmann, T.G., Frappart, P.-O., and Martins, R.A.P. (2020). Atrip protects progenitor cells against dna damage *in vivo*. Cell Death Dis. *11*, 923.

Matos-Rodrigues, G.E., and Martins, R.A.P. (2021). An eye in the replication stress response: lessons from tissue-specific studies *in vivo*. Front. Cell Dev. Biol. *9*, 731308.

Méjécase, C., Harding, P., Sarkar, H., Eintracht, J., Lima Cunha, D., Toualbi, L., and Moosajee, M. (2020). Generation of two human control ips cell lines (ucli016-a and ucli017-a) from healthy donors with no known ocular conditions. Stem Cell Res. *49*, 102113.

Mellough, C.B., Collin, J., Khazim, M., White, K., Sernagor, E., Steel, D.H.W., and Lako, M. (2015). Igf-1 signaling plays an important role in the formation of three-dimensional laminated neural retina and other ocular structures from human embryonic stem cells. Stem Cell. *33*, 2416–2430.

Mic, F.A., Molotkov, A., Molotkova, N., and Duester, G. (2004). Raldh2 expression in optic vesicle generates a retinoic acid signal needed for invagination of retina during optic cup formation. Dev. Dyn. *231*, 270–277.

Miosge, N., Holzhausen, S., Zelent, C., Sprysch, P., and Herken, R. (2001). Nidogen-1 and nidogen-2 are found in basement membranes during human embryonic development. Histochem. J. *33*, 523–530.

Miquelajáuregui, A., Varela-Echavarría, A., Ceci, M.L., García-Moreno, F., Ricaño, I., Hoang, K., Frade-Pérez, D., Portera-Cailliau, C., Tamariz, E., De Carlos, J.A., et al. (2010). Lim-homeobox gene lhx5 is required for normal development of cajal-retzius cells. J. Neurosci. *30*, 10551–10562.

Monnot, P., Gangatharan, G., Baraban, M., Pottin, K., Cabrera, M., Bonnet, I., and Breau, M.A. (2022). Intertissue mechanical interactions shape the olfactory circuit in zebrafish. Embo Rep. *23*, e52963.

Mysore, K.R., Shneider, B.L., and Harpavat, S. (2019). Biliary atresia as a disease starting *in utero*: implications for treatment, diagnosis, and pathogenesis. J. Pediatr. Gastroenterol. Nutr. *69*, 396–403.

Oltean, A., Huang, J., Beebe, D.C., and Taber, L.A. (2016). Tissue growth constrained by extracellular matrix drives invagination during optic cup morphogenesis. Biomech. Model. Mechanobiol. *15*, 1405–1421.

Parfitt, D.A., Lane, A., Ramsden, C.M., Carr, A.J.F., Munro, P.M., Jovanovic, K., Schwarz, N., Kanuga, N., Muthiah, M.N., Hull, S., et al. (2016). Identification and correction of mechanisms underlying inherited blindness in human ipsc-derived optic cups. Cell Stem Cell *18*, 769–781.

Patel, A., Hayward, J.D., Tailor, V., Nyanhete, R., Ahlfors, H., Gabriel, C., Jannini, T.B., Abbou-Rayyah, Y., Henderson, R., Nischal, K.K., et al. (2019). The oculome panel test: next-generation sequencing to diagnose a diverse range of genetic developmental eye disorders. Ophthalmology *126*, 888–907.

Phillips, M.J., Perez, E.T., Martin, J.M., Reshel, S.T., Wallace, K.A., Capowski, E.E., Singh, R., Wright, L.S., Clark, E.M., Barney, P.M., et al. (2014). Modeling human retinal development with patient-specific induced pluripotent stem cells reveals multiple roles for visual system homeobox 2. Stem Cell. *32*, 1480–1492.

Plaisancié, J., Ceroni, F., Holt, R., Zazo Seco, C., Calvas, P., Chassaing, N., and Ragge, N.K. (2019). Genetics of anophthalmia and microphthalmia. Part 1: non-syndromic anophthalmia/microphthalmia. Hum. Genet. *138*, 799–830.

Rodrigues-Amorim, D., Rivera-Baltanás, T., Fernández-Palleiro, P., Iglesias-Martínez-Almeida, M., Freiría-Martínez, L., Jarmardo-Rodriguez, C., Del Carmen Vallejo-Curto, M., Álvarez-Ariza, M., López-García, M., De Las Heras, E., et al. (2022). Changes in the brain extracellular matrix composition in schizophrenia: a pathophysiological dysregulation and a potential therapeutic target. Cell. Mol. Neurobiol. *42*, 1921–1932.

Rodrigues, P.M.G., Grigaravicius, P., Remus, M., Cavalheiro, G.R., Gomes, A.L., Rocha-Martins, M., Frappart, L., Reuss, D., Mckinnon, P.J., Von Deimling, A., et al. (2013). Nbn and atm cooperate in a tissue and developmental stage-specific manner to prevent double strand breaks and apoptosis in developing brain and eye. PLoS One *8*, e69209.

Sakamaki, K., and Satou, Y. (2009). Caspases: evolutionary aspects of their functions in vertebrates. J. Fish. Biol. *74*, 727–753.

Serjanov, D., Bachay, G., Hunter, D.D., and Brunken, W.J. (2018). Laminin β2 chain regulates retinal progenitor cell mitotic spindle orientation via dystroglycan. J. Neurosci. *38*, 5996–6010.

Sijilmassi, O., Del Río Sevilla, A., Maldonado Bautista, E., and Barrio Asensio, M.D.C. (2021). Gestational folic acid deficiency alters embryonic eye development: possible role of basement membrane proteins in eye malformations. Nutrition *90*, 111250.

Smets, K.J., Barlow, T., and Vanhaesebrouck, P. (2006). Maternal vitamin a deficiency and neonatal microphthalmia: complications of biliopancreatic diversion? Eur. J. Pediatr. *165*, 502–504.

Song, Y., Zhang, F., Zhao, Y., Sun, M., Tao, J., Liang, Y., Ma, L., Yu, Y., Wang, J., and Hao, J. (2016). Enlargement of the axial length and altered ultrastructural features of the sclera in a mutant lumican transgenic mouse model. PLoS One *11*, e0163165.

Soto, J.L., Cabrera, C.M., Serrano, S., and López-Nevot, M.A. (2005). Mutation analysis of genes that control the g1/s cell cycle in melanoma: tp53, cdkn1a, cdkn2a, and cdkn2b. Bmc Cancer *5*, 36.

Steinfeld, J., Steinfeld, I., Coronato, N., Hampel, M.-L., Layer, P.G., Araki, M., and Vogel-Höpker, A. (2013). Rpe specification in the chick is mediated by surface ectoderm-derived bmp and wnt signalling. Development *140*, 4959–4969.

Takeda, S., Sasagawa, S., Oyama, T., Searleman, A.C., Westergard, T.D., Cheng, E.H., and Hsieh, J.J. (2015). Taspase1-dependent tfiia cleavage coordinates head morphogenesis by limiting cdkn2a locus transcription. J. Clin. Invest. *125*, 1203–1214.

Teotia, P., Van Hook, M.J., Wichman, C.S., Allingham, R.R., Hauser, M.A., and Ahmad, I. (2017). Modeling glaucoma: retinal ganglion cells generated from induced pluripotent stem cells of patients with six6 risk allele show developmental abnormalities. Stem Cell. *35*, 2239–2252.

Treff, N.R., Marin, D., Lello, L., Hsu, S., and Tellier, L.C.A.M. (2020). Preimplantation genetic testing: preimplantation genetic testing for polygenic disease risk. Reproduction *160*, a13–a17.

Trejo-Reveles, V., Owen, N., Ching Chan, B.H., Toms, M., Schoenebeck, J.J., Moosajee, M., and Rainger, J.; Genomics England Research Consortium (2023). Identification of novel coloboma candidate genes through conserved gene expression analyses across four vertebrate species. Biomolecules *13*, 293.

Tummers, B., and Green, D.R. (2017). Caspase-8: regulating life and death. Immunol. Rev. *277*, 76–89.

Turksen, K., Aubin, J.E., Sodek, J., and Kalnins, V.I. (1985). Localization of laminin, type iv collagen, fibronectin, and heparan sulfate proteoglycan in chick retinal pigment epithelium basement membrane during embryonic development. J. Histochem. Cytochem. *33*, 665–671.

Wang, G.-F., Ji, Q.-S., Qi, B., Yu, G.-C., Liu, L., and Zhong, J.-X. (2017). The association of lumican polymorphisms and high myopia in a southern chinese population. Int. J. Ophthalmol. *10*, 1516–1520.

Wang, L., Hiler, D., Xu, B., Aldiri, I., Chen, X., Zhou, X., Griffiths, L., Valentine, M., Shirinifard, A., Sablauer, A., et al. (2018). Retinal cell type dna methylation and histone modifications predict reprogramming efficiency and retinogenesis in 3d organoid cultures. Cell Rep. *22*, 2601–2614.

Wang, Y., Zheng, L., Shang, W., Yang, Z., Li, T., Liu, F., Shao, W., Lv, L., Chai, L., Qu, L., et al. (2022). Wnt/beta-catenin signaling confers ferroptosis resistance by targeting gpx4 in gastric cancer. Cell Death Differ. *29*, 2190–2202.

Williamson, K.A., and Fitzpatrick, D.R. (2014). The genetic architecture of microphthalmia, anophthalmia and coloboma. Eur. J. Med. Genet. *57*, 369–380.

Williamson, K.A., Hall, H.N., Owen, L.J., Livesey, B.J., Hanson, I.M., Adams, G.G.W., Bodek, S., Calvas, P., Castle, B., Clarke, M., et al. (2020). Recurrent heterozygous pax6 missense variants cause severe bilateral microphthalmia via predictable effects on dna–protein interaction. Genet. Med. *22*, 598–609.

Wimplinger, I., Shaw, G.M., and Kutsche, K. (2007). Hccs loss-of-function missense mutation in a female with bilateral microphthalmia and sclerocornea: a novel gene for severe ocular malformations. Mol. Vis. *13*, 1475–1482.

Wu, L.-Y., Li, M., Hinton, D.R., Guo, L., Jiang, S., Wang, J.T., Zeng, A., Xie, J.B., Snead, M., Shuler, C., et al. (2003). Microphthalmia resulting from msx2-induced apoptosis in the optic vesicle. Invest. Ophthalmol. Vis. Sci. *44*, 2404–2412.

Xia, Y., Liu, Y., Yang, C., Simeone, D.M., Sun, T.T., Degraff, D.J., Tang, M.S., Zhang, Y., and Wu, X.R. (2021). Dominant role of cdkn2b/p15ink4b of 9p21.3 tumor suppressor hub in inhibition of cell-cycle and glycolysis. Nat. Commun. *12*, 2047.

Xu, S., Sunderland, M.E., Coles, B.L.K., Kam, A., Holowacz, T., Ashery-Padan, R., Marquardt, T., Mcinnes, R.R., and Van Der Kooy, D. (2007). The proliferation and expansion of retinal stem cells require functional pax6. Dev. Biol. *304*, 713–721.

Yamaguchi, M., Tonou-Fujimori, N., Komori, A., Maeda, R., Nojima, Y., Li, H., Okamoto, H., and Masai, I. (2005). Histone deacetylase 1 regulates retinal neurogenesis in zebrafish by suppressing wnt and notch signaling pathways. Development *132*, 3027–3043.

Yeh, L.-K., Liu, C.-Y., Kao, W.W.-Y., Huang, C.-J., Hu, F.-R., Chien, C.-L., and Wang, I.-J. (2010). Knockdown of zebrafish lumican gene (zlum) causes scleral thinning and increased size of scleral coats. J. Biol. Chem. *285*, 28141–28155.

Yoon, K.H., Fox, S.C., Dicipulo, R., Lehmann, O.J., and Waskiewicz, A.J. (2020). Ocular coloboma: genetic variants reveal a dynamic model of eye development. Am. J. Med. Genet. C Sem. Med. Genet. *184*, 590–610.

Yu, Z., Yu, W., Liu, J., Wu, D., Wang, C., Zhang, J., and Zhao, J. (2018). Lens-specific deletion of the msx2 gene increased apoptosis by enhancing the caspase-3/caspase-8 signaling pathway. J. Int. Med. Res. *46*, 2843–2855.

Zhu, L., Gomez-Duran, A., Saretzki, G., Jin, S., Tilgner, K., Melguizo-Sanchis, D., Anyfantis, G., Al-Aama, J., Vallier, L., Chinnery, P., et al. (2016). The mitochondrial protein chchd2 primes the differentiation potential of human induced pluripotent stem cells to neuroectodermal lineages. J. Cell Biol. *215*, 187–202.

Stem Cell Reports, Volume *19*

Supplemental Information

Disruption of common ocular developmental pathways in patient-derived optic vesicle models of microphthalmia

Jonathan Eintracht, Nicholas Owen, Philippa Harding, and Mariya Moosajee

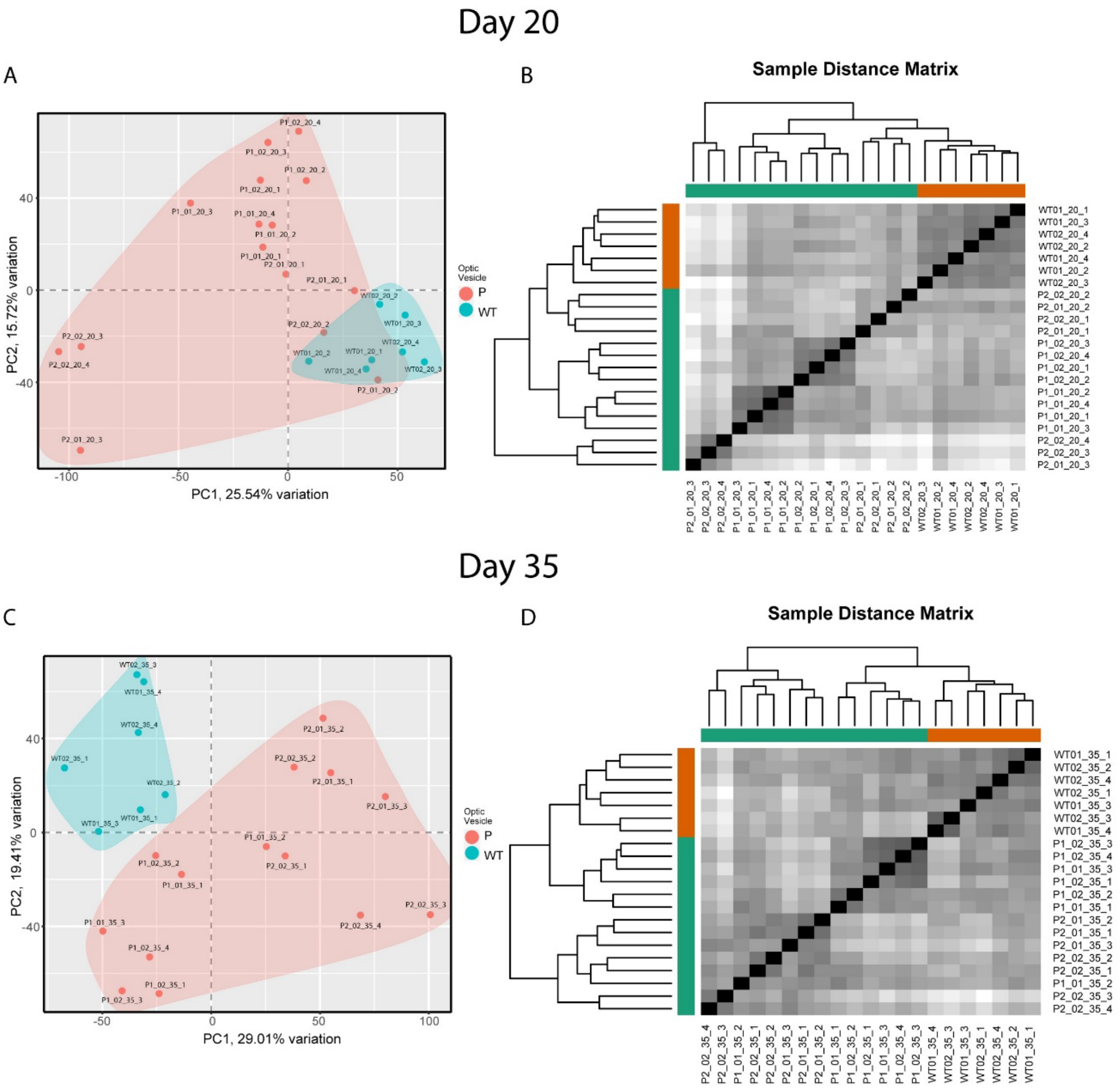

Supplementary Figure 1: Principal Component Analysis (PCA) and Sample Distance Matrix (SDM) analysis of P v WT vesicles at day 20 and day 35. (a-b) PCA and SDM showing separation of both P1 and P2 samples from wild type controls at day 20. Duplicate clones from each patient and wild type iPSC line are represented as either clone 01 or clone 02. (c-d) PCA and SDM showing separation of both P1 and P2 samples from wild type controls at day 35. Duplicate clones from each patient and wild type iPSC line are represented as either clone 01 or clone 02.

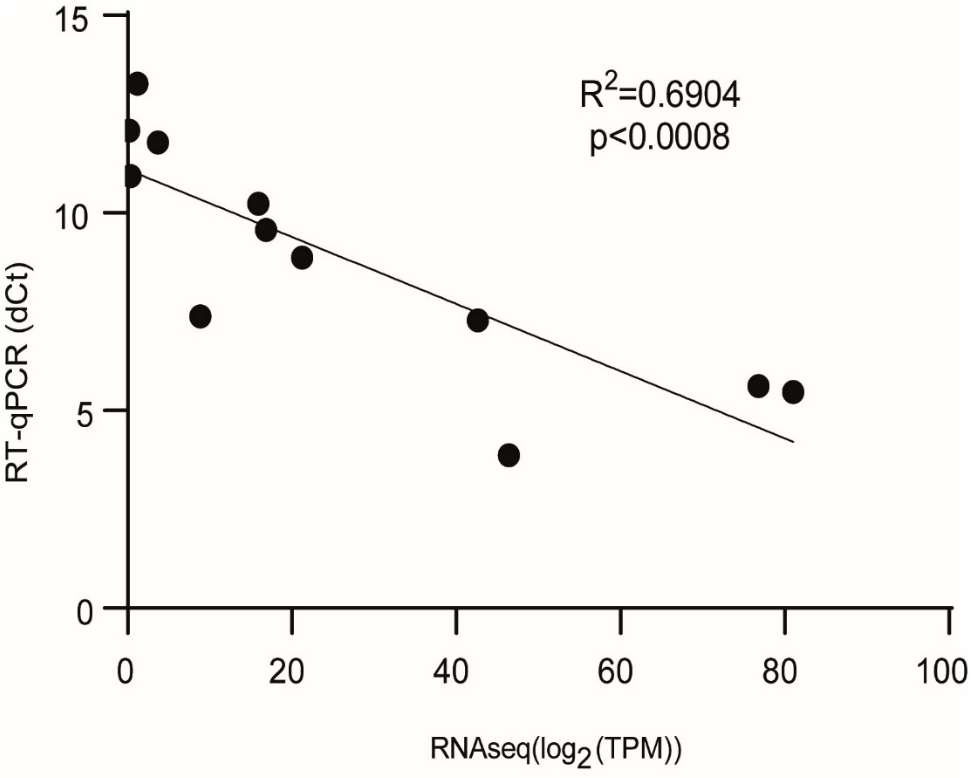

Supplementary Figure 2: Validation of robust control RNA-seq data with TPM data correlating to RT-qPCR gene expression. Scatterplot of optic vesicle sample RNA-seq normalised count data (TPM – transcripts per million) against normalised RT-qPCR ΔC_t values for 6 key ocular development transcription factors (*VSX2, MITF, RAX, PAX6, SOX2, SIX6*) at day 20 and 35 of differentiation with significant correlation (Spearman correlation R^2=0.6904, *p* value < 0.0008)

Table S1: Clinically observed patient phenotypes

	Ocular Phenotype	Non-ocular phenotype
**Patient 1 (P1) 16 year old male**	▪ Bilateral microphthalmia with axial length right eye 19.87 mm and left eye 20.03 mm<br>▪ Bilateral microcornea<br>▪ Bilateral iris and chorioretinal coloboma involving the macular and optic disc<br>▪ Best corrected visual acuity (BCVA) right eye no light perception and left eye 1.00 LogMAR.	N/A
**Patient 2 (P2) 35 year old female**	▪ Unilateral left microphthalmia with axial length right eye 23.1 mm and left eye 20.5 mm<br>▪ Right eye exhibited a complex phenotype of partial iris hypoplasia, microcornea, aphakic (following cataract surgery) with chorioretinal coloboma involving the optic disc<br>▪ Left eye microphthalmia with microcornea, cataract, no posterior view<br>▪ Best corrected visual acuity (BCVA) right eye 1.00 LogMAR and left eye no perception to light.	Type II diabetes mellitus<br>Obesity

Table S6 – Primer Sequences

Primer	Sequence (5'-3')	Product size (bp)
OCT4 RT-qPCR	F - CCCCAGGGCCCCATTTTGGTACC<br>R - ACCTCAGTTTGAATGCATGGGAGAGC	143
SOX2 RT-qPCR	F - TTCACATGTCCCA GCACTACCAGA<br>R - TCACATGTGTGAG AGGGGCAGTGTGC	80
LIN28 RT-qPCR	F - AGCCATATGGTAG CCTCATGTCCGC<br>R - TCAATTCTGTGCCT CCGGGAGCAGGG TAGG	129
c-MYC RT-qPCR	F - GCGAACCCAAGAC CCAGGCCTGCTCC<br>R - CAGGGGGTCTGCT CGCACCGTGATG	143
GAPDH RT-qPCR	F - ACAGTTGCCATGT AGACC<br>R - TTTTTGGTTGAGCA CAGG	95
OCT4 Plasmid RT-qPCR	F - CATTCAAACTGAG GTAAGGG<br>R - TAGCGTAAAAGGA GCAACATAG	124
SOX2 Plasmid RT-qPCR	F - TTCACATGTCCCA GCACTACCAGA<br>R - TTTGTTTGACAGGA GCGACAAT	111
c-MYC Plasmid RT-qPCR	F - GGCTGAGAAGAGG ATGGCTAC<br>R - TTTGTTTGACAGGA GCGACAAT	122
LIN28 Plasmid RT-qPCR	F - AGCCATATGGTAG CCTCATGTCCGC<br>R - TAGCGTAAAAGGA GCAACATAG	251
KLF4 Plasmid RT-qPCR	F - CCACCTCGCCTTA CACATGAAGA<br>R - TAGCGTAAAAGGA GCAACATAG	156
EBNA Plasmid RT-qPCR	F - ATCAGGGCCAAGA CATAGAGATG<br>R - GCCAATGCAACTT GGACGTT	61
PAX6 RT-qPCR	F - GGCCGAACAGACA CAGCCCTCAC<br>R - ATCATAACTCCGC CCATTCACC	165
OTX2 RT-qPCR	F - TAAAAATTGCTAGA GCAGCC<br>R - CATGGGAGGTTAG AAAAAGTC	248
RAX RT-qPCR	F - AGGCGGAAAAATA GAGTTTG<br>R - TACCCCAATATTCA CTCCTC	139
LHX2 RT-qPCR	F - TTTTCTAATGACTC GCAACC<br>R - TTAGTTAGTTGCTC AAAGCC	108
VSX2 RT-qPCR	F - GGCGACACAGGAC AATCTTTA<br>R - TTCCGGCAGCTCC GTTTTC	122
MITF RT-qPCR	F - CAGTACCTTTCTAC CACTTTAG<br>R - CCTCTTTTTCACAG TTGGAG	163
LUM RT-qPCR	F – AAGGATTCAAACCATTTGCC<br>R -TCAATTTCAGCTCATCACAG	199

NR2F1<br>RT-qPCR	F - CCGGCGTGAATTATCCCGTA<br>R - GCTCTTTTTGTTGTGCCGGT	96
CASP8<br>RT-qPCR	F - CTACAGGGTCATGCTCTATC<br>R - ATTTGGAGATTTCCTCTTGC	92
LHX5<br>RT-qPCR	F - TTTCACCTCAACTGTTTCAC<br>R - TACAGGATGACACTGAGTTG	157

Table S7 – Primary and secondary antibodies

Primary Antibodies						
**Antibody**	**Species**	**Supplier**	**Catalogue #**	**RRID**	**Dilution**	**Application**
OCT4	Mouse	Santa Cruz Biotechnology	sc-5279	AB_628051	1:100	IF
SSEA3	Rat	Millipore	MAB4303	AB_177628	1:50	IF
AFP	Mouse	Santa Cruz Biotechnology	sc-51506	AB_626514	1:300	IF
Vimentin	Mouse	Santa Cruz Biotechnology	sc-6260	AB_628437	1:250	IF
PAX6	Rabbit	Covance	PRB-278P	AB_291612	1:100	IF
VSX2 (CHX10)	Mouse	Santa Cruz Biotechnology	sc-365519	AB_10842442	1:200 (IF), 1:1000(WB)	IF/WB
PH3	Rabbit	Cell Signaling Technology	9701	AB_1549592	1:500	IF
COL IV	Rabbit	Abcam	ab6586	AB_305584	1:100 (IF), 1:1000(WB)	IF/WB
LUM	Rabbit	Abcam	ab168348	AB_2920864	1:100 (IF), 1:1000(WB)	IF/WB
NID2	Rabbit	Abcam	ab14513	AB_301292	1:200 (IF), 1:1000(WB)	IF/WB
β-actin	Mouse	Sigma-Aldrich	A2228	AB_476697	1:5000	WB

Secondary Antibodies					
**Antibody**	**Species**	**Supplier**	**Catalogue #**	**RRID**	**Dilution**
Alexa Fluor® 488	Goat anti-Rat	Thermo Fisher Scientific	A-11006	AB_2534074	1:800
Alexa Fluor® 488	Goat anti-Rabbit	Thermo Fisher Scientific	A32731	AB_2633280	1:800
Alexa Fluor® 488	Donkey anti-Goat	Thermo Fisher Scientific	A-11055	AB_2534102	1:800

Alexa Fluor® 647	Donkey anti-Goat	Thermo Fisher Scientific	A-21447	AB_2535864	1:800
Alexa Fluor® 647	Goat anti-Mouse	Thermo Fisher Scientific	A-21235	AB_2535804	1:800
Polyclonal Goat Anti-Mouse Immunoglobulin antibody	Goat Anti-Mouse	Dako, Denmark	P0447	AB_2617137	1:10 000
Polyclonal Goat Anti-Rabbit Immunoglobulin antibody	Goat Anti-Rabbit	Dako, Denmark	P0448	AB_2617138	1:10 000

Supplementary Experimental Procedures

hiPSC derivation and culture

In brief, fibroblasts were derived from skin biopsies after overnight incubation and cultured 1×10^6 fibroblast cells were electroporated (1600 V, 20ms, 3 pulses) with 1 µg each of four episomal plasmids (pCXLE-hSK (Addgene ID# 27078), pCXLE-hUL (Addgene ID# 27080), pCXLE-hOCT3/4-shp53-F (Addgene ID# 27077) and pCXWB-EBNA1 (Addgene ID# 37624)) using the Neon Transfection System (Parfitt et al., 2016). Transfected cells were plated on 0.1% gelatin-coated 100 mm dishes in fibroblast media with 0.5 mM sodium butyrate (Sigma-Aldrich, cat#B5587). After seven days, cells were dissociated with TrypLE Express and 200,000 cells plated into each well of a Matrigel-coated (Corning, USA, cat#354377) 6-well plate in mTeSR Plus (STEMCELL Technologies, Canada, cat#1000276). Rudimentary iPSC colonies were excised from these plates and cultured in isolation. iPSCs were maintained in mTeSR Plus and passaged using ReleSR (STEMCELL Technologies, Canada, cat#05872).(Parfitt et al., 2016). Transfected cells were plated on 0.1% gelatin-coated 100 mm dishes in fibroblast media with 0.5 mM sodium butyrate (Sigma-Aldrich, cat#B5587). After seven days, cells were dissociated with TrypLE Express and 200,000 cells plated into each well of a Matrigel-coated (Corning, USA, cat#354377) 6-well plate in mTeSR Plus (STEMCELL Technologies, Canada, cat#1000276). Rudimentary iPSC colonies were excised from these plates and cultured in isolation. iPSCs were maintained in mTeSR Plus and passaged using ReleSR (STEMCELL Technologies, Canada, cat#05872).

Embryoid body formation

EBs were formed in Aggrewell™ plates, a plate where each well of a 24-well plate is comprised of 1200 microwells, as per manufacturer's instructions (STEMCELL Technologies, Canada, cat#34415). Briefly, iPSCs were washed with PBS and detached with Accumax (ThermoFisher Scientific, USA, cat#00-4666-56) to form a single cell suspension. After 5-8 minutes, mTeSR Plus media was added to each well. Cells were counted using Countess™ II Automated Cell Counter (ThermoFisher Scientific, USA). 3.6x106 cells per well were centrifuged and resuspended in 1mL mTeSR Plus with 10µM Y-27632 and added to one well of. the Aggrewell™ plate (3000 cells per microwell). Mixing with a pipette was required to ensure uniform distribution of cells. The plate was spun at 100g for three minutes and incubated at 37°C. After 24 hours, 1mL media was changed (day 1).

Optic vesicle differentiation

EBs were formed as previously described (Eintracht et al., 2022), and differentiation was performed as outlined (Mellough et al., 2015, Chichagova et al., 2019). 48 hours after EB formation (day 2), EBs were plated by gentle pipetting into 60mm TC-treated culture dishes (Appleton Woods, UK, cat#BF152) and cultured in Neural Induction Media (NIM), (DMEM/F12 (ThermoFisher Scientific, cat#31331028), 20% knock-out serum residue (KOSR) (ThermoFisher Scientific, cat#10828028, 2% B27

(ThermoFisher Scientific, USA, cat#17504001), 1xNon-Essential Amino Acids (NEAA; ThermoFisher Scientific, USA, cat#11140050), 1% P/S, 1xGlutamax (ThermoFisher Scientific, USA, cat#35050061) and 5ng/mL IGF-1 (Sigma-Aldrich, USA, cat#I3769). One well of an Aggrewell™ plate was transferred into six uncoated 60mm round dishes, resulting in a final density of approximately 200 EBs per 60mm culture dish, or 1200 EBs per $3.6x10^6$ cells.

Cells were cultured in NIM with decreasing KOSR concentrations, 20% from day 2-7, 15% from day 7-11 and 10% from day 11-18. From day 18, cells were cultured in Retinal Differentiation Media (DMEM/F12, 10% FBS, 2% B27, 1xNEAA, 1xGlutamax, 1% P/S, 5ng/mL IGF-1, 0.1mM taurine (Sigma-Aldrich, USA, cat#T8691), 40ng/mL triiodothyronine (Sigma-Aldrich, USA, cat#T6397) and 0.5µM retinoic acid (Sigma-Aldrich, USA, cat#R2625) added immediately before use. Cells were cultured in RDM until day 50. OV diameters were measured as described above.

Treatment with caspase-8 inhibitor Z-IETD-FMK

To prepare for cell culture applications, 1mg of caspase-8 inhibitor Z-IETD-FMK was reconstituted in 150µL DMSO to a concentration of stock concentration of 10mM. This was diluted to a working concentration of 40µM in cell culture media based on previous in vitro experiments (Shi et al., 2018, Monari et al., 2008, Yuan et al., 2018). OVs were treated with 40µM Z-IETD-FMK on day 33 and day 34, beginning 48 hours prior to harvest on day 35. The total concentration of DMSO in culture did not exceed 0.2% to prevent cytotoxic effects.

Optic vesicle diameter measurements

To measure OV diameters, the diameters of between 5-20 individual vesicles from brightfield images for each condition were measured using ImageJ (NIH, USA). Initially, diameters were measured in pixels using the ImageJ software by drawing a line across each vesicle and using the 'Measure' function. Lengths in pixels were converted to micrometres based on the manufacturers' data. For the 2x objective, the pixel size was 3.0854µm/pixel; for the 4x objective it was 1.5427µm/pixel and for the 10x objective it was 0.6172 µm/pixel.

RNA isolation, RT-qPCR and RNA-seq analysis

Each individual plate of OVs was collected at either day 0, day 20 or day 35 and RNA extraction was performed using the RNeasy Mini Kit (QIAGEN, Germany, cat#74104). cDNA was synthesized from 1µg using the SuperScript III First Strand cDNA synthesis kit (Invitrogen, USA, cat#18080093) according to manufacturer's instructions. RT-qPCR was performed using 2x SYBR Green Master Mix (ThermoFisher Scientific, USA, cat#4472908) as per manufacturer's instructions on the StepOne Real-Time PCR system (Applied Biosystems, ThermoFisher, UK). Primers used for the qRT-PCR at 200nM are listed below **(Table S6)** and were designed using the Primer-BLAST tool from the National Centre for Biotechnology Information. All transcript levels were measured in triplicate and normalised to

GAPDH, with undetermined C_t values in negative controls where no cDNA was present. The relative expression of each target gene compared to iPSCs at day 0 of differentiation was calculated using the comparative C_t or $2^{-\Delta\Delta Ct}$ method (Schmittgen and Livak, 2008). Statistical comparisons and figures were generated using GraphPad Prism (GraphPad Software, USA). The relative expression of each target gene compared to iPSCs at day 0 of differentiation was calculated using the comparative C_t or $2^{-\Delta\Delta Ct}$ method (Schmittgen and Livak, 2008). Statistical comparisons and figures were generated using GraphPad Prism (GraphPad Software, USA).

RNA-seq library construction was performed following the manufacturer's instructions using TruSeq stranded Total RNA with Ribo Zero Plus rRNA depletion (Illumina cat 20020599). Subsequent libraries were run on a bioanalyzer to verify size and concentration prior to sequencing on the NovaSeq 6000 platform at Novogen, UK. Raw sequence reads were assessed for quality issues and adapter sequences, or other sources of contamination using fastqc (https://www.bioinformatics.babraham.ac.uk/projects/fastqc/). Low quality sequences with PHRED quality scores below 6 were trimmed from reads using trim galore (v0.6.6, https://www.bioinformatics.babraham.ac.uk/projects/trim_galore/). Reads passing these quality filters were aligned using HISAT2 (v2.2.1) to the Ensembl reference genome build GRCh38 and annotation version 102 (obtained Feb 2021) (Kim et al., 2019). Gene count tables were generated for all samples using featurecounts from the subread package (v2.0.0), and differential expression analysis carried out with DESeq2 v1.34 (Love et al., 2014). Genes with an absolute log2 fold change of ≥1 and an FDR ≤ 0.05 were classified as differentially expressed. Further downstream analysis, including gene ontology (GO) over representation analysis, gene set enrichment analysis (GSEA), data visualisation and statistical tests were conducted using R for statistics v4.1.2. Initially P1 and 2 data were grouped for pairwise comparison with WT, subsequent analysis investigated all possible pairwise comparisons. Functional classifications of DEGs was analysed by GO ORA using gProfiler and by Gene Set Enrichment Analysis (GSEA) Bioconductor packages GAGE (Luo et al., 2009) and fgsea (Korotkevich et al., 2021) (Subramanian et al., 2005) using unfiltered ranked gene list. Molecular Signature Databases were obtained from MsigDB (v7.4), including pathways with curated gene sets from online pathway databases, biomedical literature and contributions from domain experts. This collection comprised of KEGG (http://www.genome.jp/kegg/pathway.html), Reactome (http://www.reactome.org/), Gene Ontologies (BP/MF/CC), Canonical Pathways, and WikiPathways.

Western Blotting

Samples were analysed by western blotting as previously described (Eintracht et al., 2021). Briefly, cells were washed with ice-cold PBS and total protein extract was prepared with RIPA buffer (ThermoFisher Scientific, MA, USA, cat#89900) at a ratio of 5×10^6 cells/mL buffer and 1 x Halt™ protease and phosphatase inhibitor cocktail (ThermoFisher Scientific, MA, USA, cat#78440).

Proteins were resolved on 4–15% Mini-PROTEAN® TGX ™ gels (Bio-Rad Inc., CA, USA, cat#4561025) and transferred to an Immun-Blot™ PVDF membrane using a Trans-Blot® SD semi-dry transfer cell (Bio-Rad Inc., CA, USA). Membranes were blocked with 5% non-fat dry milk in 0.1% PBS/T for 1 h. A complete list of primary and secondary antibodies and appropriate dilutions can be found in **Table S7**. Blots were scanned using the ChemiDoc XRS ™ Imaging System (Bio-Rad Inc., CA, USA) and quantitatively analysed using the Fiji/ImageJ software (National Institutes of Health, MD, USA).

Embedding and cryoembedding of vesicles

OVs were fixed in 4% paraformaldehyde (Fisher Scientific, UK, cat# for 10-20 minutes at 4°C, washed three times with PBS and stored overnight at 4°C in 30% sucrose (Sigma-Aldrich, USA, cat#S0389). Vesicles were embedded individually in 1.5cm x 1.5cm x 0.5cm moulds (Fisher Scientific, UK, cat#11670990) containing 800µL 7.5% gelatin/10% sucrose (Sigma-Aldrich, USA cat#G2500) solution and left to solidify at 4°C overnight. Embedded vesicles were excised from their moulds and placed in OCT embedding media (Agar Scientific, UK, cat#AGR1180) prior to snap-freezing in -50°C 2-methylbutane using a small dewar in a fumehood (Sigma-Aldrich, USA, cat#M32631) for three minutes. Frozen blocks were stored at -80°C.

Immunohistochemistry

Vesicles were sectioned using the Leica CM 3050 S cryostat at a thickness of 10µm and slides were left at RT for 1-2 hrs. Slides were washed twice for five minutes in PBS+0.1%Tween®20 (PBS/T) (Sigma-Aldrich, USA, cat#P1379) and permeabilized in PBS/T+0.5% Triton-X (Fisher Scientific, UK, cat#10591461) for one hour at RT with slight agitation. Samples were washed with PBS/T for five minutes and then blocked for one hour at RT in PBS+0.2% gelatin+0.5% Triton-X. Samples were incubated with primary antibodies overnight at 4°C (primary antibodies and dilutions are listed below in **Table S7**). Samples were subsequently washed three times for ten minutes with PBS/T. Samples were then incubated with secondary antibodies for one hour at RT in the dark (secondary antibodies and dilutions are listed below in **Table S7**). Samples were washed again three times for five minutes with PBS/T and DAPI (Sigma Aldrich, UK, cat#D9542) and once for five minutes with PBS. Slides were dipped in 100% ethanol and left to dry at RT. Once dry, coverslips were mounted with ProLong™ Diamond Antifade Mountant (ThermoFisher Scientific, USA, cat#P36961) and left to set overnight at RT in the dark. Slides were imaged using the confocal microscopes ZEISS LSM 700 and LSM 710 (ZEISS Research, Germany) and figures were generated using ImageJ (NCBI, USA) and Adobe Illustrator (Adobe Inc, USA).

TUNEL Assay

Four or five OVs from each condition were fixed, cryoembedded and sectioned as described above. Apopotic cell death was detected using the ApopTag Fluorescein *In Situ* Apoptosis Detection Kit (Merck Life Sciences, UK, cat#S7110) as per manufacturer's instructions. Sections were washed with DAPI and sealed with ProLong™ Diamond Antifade Mountant as described above. Slides were imaged as described above and stacked images collated on ImageJ (NCBI, USA). Levels of apoptotic cell death were quantified using the cell counter plugin on ImageJ (NCBI, USA).

Caspase-8 Colorimetric Assay

OVs from all conditions were collected and snap frozen at day 35. Protein was extracted and caspase-8 activity was measured using the Caspase-8 Colorimetric Assay Kit (R&D Systems, USA, cat#K113-100), as per manufacturer's conditions. Absorbance was measured using the EMax Plate Reader (Molecular Devices, USA). Data analysis was performed by Microsoft Excel (Microsoft, USA) and figures generated using GraphPad Prism (GraphPad Software, USA).

References

CHICHAGOVA, V., DORGAU, B., FELEMBAN, M., GEORGIOU, M., ARMSTRONG, L. & LAKO, M. 2019. Differentiation of Retinal Organoids from Human Pluripotent Stem Cells. *Current Protocols in Stem Cell Biology,* 50**,** e95.

EINTRACHT, J., FORSYTHE, E., MAY-SIMERA, H. & MOOSAJEE, M. 2021. Translational readthrough of ciliopathy genes BBS2 and ALMS1 restores protein, ciliogenesis and function in patient fibroblasts. *EBioMedicine,* 70**,** 103515.

EINTRACHT, J., HARDING, P., CUNHA, D. L. & MOOSAJEE, M. 2022. Efficient embryoid-based method to improve generation of optic vesicles from human induced pluripotent stem cells. *F1000Research,* 11**,** 324.

KIM, D., PAGGI, J. M., PARK, C., BENNETT, C. & SALZBERG, S. L. 2019. Graph-based genome alignment and genotyping with HISAT2 and HISAT-genotype. *Nature biotechnology,* 37**,** 907-915.

KOROTKEVICH, G., SUKHOV, V., BUDIN, N., SHPAK, B., ARTYOMOV, M. N. & SERGUSHICHEV, A. 2021. Fast gene set enrichment analysis. *BioRxiv***,** 060012.

LOVE, M. I., HUBER, W. & ANDERS, S. 2014. Moderated estimation of fold change and dispersion for RNA-seq data with DESeq2. *Genome biology,* 15**,** 1-21.

LUO, W., FRIEDMAN, M. S., SHEDDEN, K., HANKENSON, K. D. & WOOLF, P. J. 2009. GAGE: generally applicable gene set enrichment for pathway analysis. *BMC bioinformatics,* 10**,** 1-17.

MELLOUGH, C. B., COLLIN, J., KHAZIM, M., WHITE, K., SERNAGOR, E., STEEL, D. H. W. & LAKO, M. 2015. IGF-1 Signaling Plays an Important Role in the Formation of Three-Dimensional Laminated Neural Retina and Other Ocular Structures From Human Embryonic Stem Cells. *Stem Cells (Dayton, Ohio),* 33**,** 2416-2430.

MONARI, C., PAGANELLI, F., BISTONI, F., KOZEL, T. R. & VECCHIARELLI, A. 2008. Capsular polysaccharide induction of apoptosis by intrinsic and extrinsic mechanisms. *Cellular microbiology,* 10**,** 2129-2137.

PARFITT, DAVID A., LANE, A., RAMSDEN, CONOR M., CARR, A.-JAYNE F., MUNRO, PETER M., JOVANOVIC, K., SCHWARZ, N., KANUGA, N., MUTHIAH, MANICKAM N., HULL, S., GALLO, J.-M., DA CRUZ, L., MOORE, ANTHONY T., HARDCASTLE, ALISON J., COFFEY, PETER J. &

CHEETHAM, MICHAEL E. 2016. Identification and Correction of Mechanisms Underlying Inherited Blindness in Human iPSC-Derived Optic Cups. *Cell Stem Cell,* 18**,** 769-781.

SCHMITTGEN, T. D. & LIVAK, K. J. 2008. Analyzing real-time PCR data by the comparative C T method. *Nature protocols,* 3**,** 1101.

SHI, G., JIA, P., CHEN, H., BAO, L., FENG, F. & TANG, H. 2018. Necroptosis occurs in osteoblasts during tumor necrosis factor-α stimulation and caspase-8 inhibition. *Brazilian Journal of Medical and Biological Research,* 52.

SUBRAMANIAN, A., TAMAYO, P., MOOTHA, V. K., MUKHERJEE, S., EBERT, B. L., GILLETTE, M. A., PAULOVICH, A., POMEROY, S. L., GOLUB, T. R. & LANDER, E. S. 2005. Gene set enrichment analysis: a knowledge-based approach for interpreting genome-wide expression profiles. *Proceedings of the National Academy of Sciences,* 102**,** 15545-15550.

YUAN, Y., ZHANG, Y., ZHAO, S., CHEN, J., YANG, J., WANG, T., ZOU, H., WANG, Y., GU, J., LIU, X., BIAN, J. & LIU, Z. 2018. Cadmium-induced apoptosis in neuronal cells is mediated by Fas/FasL-mediated mitochondrial apoptotic signaling pathway. *Scientific Reports,* 8**,** 8837.
